# Supplementary material for: Aspects of Self-Management After Solid Organ Transplantation—A Scoping Review
Source: Nurs Rep. 2025 Aug 19;15(8):304. doi: 10.3390/nursrep15080304 (PMC12389569; doi:10.3390/nursrep15080304)
Supplement: Supplementary file 1 [file nursrep-15-00304-s001.zip › Supplementary File S1.pdf]

Supplementary File S1: Full electronic search strategies for all databases and registries

Medline (OVID)

#	**Search term**
1	Self Care/
2	Self-Management/
3	Self-Help Groups/
4	Self Administration/
5	Self Efficacy/
6	Health Literacy/
7	Healthy Lifestyle/
8	Lifestyle/
9	Activities of Daily Living/
10	Adaptation, Psychological/
11	Health Behavior/
12	or/1-11
13	self manag:.ti,ab,kw.
14	self car:.ti,ab,kw.
15	self help:.ti,ab,kw.
16	self regulat:.ti,ab,kw.
17	self monitor:.ti,ab,kw.
18	self efficac:.ti,ab,kw.
19	(symptom adj2 manag:).ti,ab,kw.
20	(life style or lifestyle).ti,ab,kw.
21	(cope or coping).ti,ab,kw.
22	health manag:.ti,ab,kw.
23	health literacy.ti,ab,kw.
24	or/13-23
25	12 or 24
26	Needs Assessment/
27	Perception/
28	Body Image/
29	Patient Satisfaction/
30	Anxiety/
31	Depression/
32	or/26-31

#	Search term
33	((needs or challeng: or concerns or problems or barriers or fear: or anxiety or depression or perspective: or experience: or perception: or doubt: or stressor: or distress: or view: or value: or attitud: or target: or priorit: or goal: or expectation:) adj2 (patients or recipients)).ti,ab,kw.
34	((needs or challeng: or concerns or problems or barriers or fear: or anxiety or depression or perspective: or experience: or perception: or doubt: or stressor: or distress: or view: or value: or attitud: or target: or priorit: or goal: or expectation:) adj4 transplant patients).ti,ab,kw.
35	((needs or challeng: or concerns or problems or barriers or fear: or anxiety or depression or perspective: or experience: or perception: or doubt: or stressor: or distress: or view: or value: or attitud: or target: or priorit: or goal: or expectation:) adj4 transplant recipients).ti,ab,kw.
36	or/33-35
37	32 or 36
38	25 or 37
39	Organ Transplantation/
40	Kidney Transplantation/
41	Lung Transplantation/
42	Liver Transplantation/
43	exp Heart Transplantation/
44	Pancreas Transplantation/
45	[Intestine, Small/tr]
46	Transplant Recipient/
47	or/39-46
48	(SOTx or SOT).ti,ab,kw.
49	(organ adj2 (graft: or transplant: or allograft:)).ti,ab,kw.
50	((heart or heart-lung or cardiac: or cardio: or thorac: or lung: or kidney or renal or liver: or hepatic: or hepato: or pancrea: or small bowel or intest:) adj2 (transplant: or graft: or allograft:)).ti,ab,kw.
51	or/48-50
52	47 or 51
53	38 and 52
54	Animals/ not Humans/
55	53 not 54

Emcare (OVID)

#	Search term
1	Self Care/
2	Self-Management/
3	Self-Help Groups/
4	Self Administration/
5	Self Efficacy/
6	Health Literacy/

#	Search term
7	Healthy Lifestyle/
8	Lifestyle/
9	Activities of Daily Living/
10	Adaptation, Psychological/
11	Health Behavior/
12	or/1-11
13	self manag:.ti,ab,kw.
14	self car:.ti,ab,kw.
15	self help:.ti,ab,kw.
16	self regulat:.ti,ab,kw.
17	self monitor:.ti,ab,kw.
18	self efficac:.ti,ab,kw.
19	(symptom adj2 manag:).ti,ab,kw.
20	(life style or lifestyle).ti,ab,kw.
21	(cope or coping).ti,ab,kw.
22	health manag:.ti,ab,kw.
23	health literacy.ti,ab,kw.
24	or/13-23
25	12 or 24
26	Needs Assessment/
27	Perception/
28	Body Image/
29	Patient Satisfaction/
30	Anxiety/
31	Depression/
32	or/26-31
33	((needs or challeng: or concerns or problems or barriers or fear: or anxiety or depression or perspective: or experience: or perception: or doubt: or stressor: or distress: or view: or value: or attitud: or target: or priorit: or goal: or expectation:) adj2 (patients or recipients)).ti,ab,kw.
34	((needs or challeng: or concerns or problems or barriers or fear: or anxiety or depression or perspective: or experience: or perception: or doubt: or stressor: or distress: or view: or value: or attitud: or target: or priorit: or goal: or expectation:) adj4 transplant patients).ti,ab,kw.
35	((needs or challeng: or concerns or problems or barriers or fear: or anxiety or depression or perspective: or experience: or perception: or doubt: or stressor: or distress: or view: or value: or attitud: or target: or priorit: or goal: or expectation:) adj4 transplant recipients).ti,ab,kw.
36	or/33-35
37	32 or 36
38	25 or 37
39	Organ Transplantation/

#	Search term
40	Kidney Transplantation/
41	Lung Transplantation/
42	Liver Transplantation/
43	exp Heart Transplantation/
44	Pancreas Transplantation/
45	[Intestine, Small/tr]
46	Transplant Recipient/
47	or/39-46
48	(SOTx or SOT).ti,ab,kw.
49	(organ adj2 (graft: or transplant: or allograft:)).ti,ab,kw.
50	((heart or heart-lung or cardiac: or cardio: or thorac: or lung: or kidney or renal or liver: or hepatic: or hepato: or pancrea: or small bowel or intest:) adj2 (transplant: or graft: or allograft:)).ti,ab,kw.
51	or/48-50
52	47 or 51
53	38 and 52
54	Animals/ not Humans/
55	53 not 54

Cochrane Library (Wiley)

#	Search term
1	[mh ^"Self Care"]
2	[mh ^Self-Management]
3	[mh ^"Self-Help Groups"]
4	[mh ^"Self Administration"]
5	[mh ^"Self Efficacy"]
6	[mh ^"Health Literacy"]
7	[mh ^"Healthy Lifestyle"]
8	[mh ^Lifestyle]
9	[mh ^"Activities of Daily Living"]
10	[mh ^"Adaptation, Psychological"]
11	[mh ^"Health Behavior"]
12	#1 OR #2 OR #3 OR #4 OR #5 OR #6 OR #7 OR #8 OR #9 OR #10 OR #11
13	("self" NEXT manag:):ti,ab,kw
14	("self" NEXT car:):ti,ab,kw
15	("self" NEXT help:):ti,ab,kw
16	("self" NEXT regulat:):ti,ab,kw
17	("self" NEXT monitor:):ti,ab,kw
18	("self" NEXT efficac:):ti,ab,kw
19	(symptom:ti,ab,kw NEAR/2 manag::ti,ab,kw)

#	Search term
20	("life style":ti,ab,kw OR lifestyle:ti,ab,kw)
21	(cope:ti,ab,kw OR coping:ti,ab,kw)
22	("health" NEXT manag:):ti,ab,kw
23	"health literacy":ti,ab,kw
24	#13 OR #14 OR #15 OR #16 OR #17 OR #18 OR #19 OR #20 OR #21 OR #22 OR #23
25	#12 OR #24
26	[mh ^"Needs Assessment"]
27	[mh ^Perception]
28	[mh ^"Body Image"]
29	[mh ^"Patient Satisfaction"]
30	[mh ^Anxiety]
31	[mh ^Depression]
32	#26 OR #27 OR #28 OR #29 OR #30 OR #31
33	((needs:ti,ab,kw OR challeng::ti,ab,kw OR concerns:ti,ab,kw OR problems:ti,ab,kw OR barriers:ti,ab,kw OR fear::ti,ab,kw OR anxiety:ti,ab,kw OR depression:ti,ab,kw OR perspective::ti,ab,kw OR experience::ti,ab,kw OR perception::ti,ab,kw OR doubt::ti,ab,kw OR stressor::ti,ab,kw OR distress::ti,ab,kw OR view::ti,ab,kw OR value::ti,ab,kw OR attitud::ti,ab,kw OR target::ti,ab,kw OR priorit::ti,ab,kw OR goal::ti,ab,kw OR expectation::ti,ab,kw) NEAR/2 (patients:ti,ab,kw OR recipients:ti,ab,kw))
34	((needs:ti,ab,kw OR challeng::ti,ab,kw OR concerns:ti,ab,kw OR problems:ti,ab,kw OR barriers:ti,ab,kw OR fear::ti,ab,kw OR anxiety:ti,ab,kw OR depression:ti,ab,kw OR perspective::ti,ab,kw OR experience::ti,ab,kw OR perception::ti,ab,kw OR doubt::ti,ab,kw OR stressor::ti,ab,kw OR distress::ti,ab,kw OR view::ti,ab,kw OR value::ti,ab,kw OR attitud::ti,ab,kw OR target::ti,ab,kw OR priorit::ti,ab,kw OR goal::ti,ab,kw OR expectation::ti,ab,kw) NEAR/4 "transplant patients":ti,ab,kw)
35	((needs:ti,ab,kw OR challeng::ti,ab,kw OR concerns:ti,ab,kw OR problems:ti,ab,kw OR barriers:ti,ab,kw OR fear::ti,ab,kw OR anxiety:ti,ab,kw OR depression:ti,ab,kw OR perspective::ti,ab,kw OR experience::ti,ab,kw OR perception::ti,ab,kw OR doubt::ti,ab,kw OR stressor::ti,ab,kw OR distress::ti,ab,kw OR view::ti,ab,kw OR value::ti,ab,kw OR attitud::ti,ab,kw OR target::ti,ab,kw OR priorit::ti,ab,kw OR goal::ti,ab,kw OR expectation::ti,ab,kw) NEAR/4 "transplant recipients":ti,ab,kw)
36	#33 OR #34 OR #35
37	#32 OR #36
38	#25 OR #37
39	[mh ^"Organ Transplantation"]
40	[mh ^"Kidney Transplantation"]
41	[mh ^"Lung Transplantation"]
42	[mh ^"Liver Transplantation"]
43	[mh "Heart Transplantation"]
44	[mh ^"Pancreas Transplantation"]
45	MeSH descriptor: [Intestine, Small] this term only and with qualifier(s): [transplantation - TR]
46	[mh ^"Transplant Recipient"]

#	Search term
47	#39 OR #40 OR #41 OR #42 OR #43 OR #44 OR #45 OR #46
48	(SOTx:ti,ab,kw OR SOT:ti,ab,kw)
49	(organ:ti,ab,kw NEAR/2 (graft::ti,ab,kw OR transplant::ti,ab,kw OR allograft::ti,ab,kw))
50	((heart:ti,ab,kw OR heart-lung:ti,ab,kw OR cardiac::ti,ab,kw OR cardio::ti,ab,kw OR thorac::ti,ab,kw OR lung::ti,ab,kw OR kidney:ti,ab,kw OR renal:ti,ab,kw OR liver::ti,ab,kw OR hepatic::ti,ab,kw OR hepato::ti,ab,kw OR pancrea::ti,ab,kw OR "small bowel":ti,ab,kw OR intest::ti,ab,kw) NEAR/2 (transplant::ti,ab,kw OR graft::ti,ab,kw OR allograft::ti,ab,kw))
51	#48 OR #49 OR #50
52	#47 OR #51
53	#38 AND #52

Web of Science (Core Collection) (Clarivate)

Science Citation Index Expanded (SCI-EXPANDED), Conference Proceedings Citation Index – Science (CPCI-S), Emerging Sources Citation Index (ESCI)

#	Search term
1	TS="self manag:"
2	TS="self car:"
3	TS="self help:"
4	TS="self regulat:"
5	TS="self monitor:"
6	TS="self efficac:"
7	TS= (symptom NEAR/2 manag:)
8	TS= "life style" OR lifestyle
9	TS="health manag:"
10	TS="psychological adaptation"
11	TS="health behavior" OR "health behaviour"
12	TS="health literacy"
13	TS= cope OR coping
14	#14 #1 OR #2 OR #3 OR #4 OR #5 OR #6 OR #7 OR #8 OR #9 OR #10 OR #11 OR #12 OR #13
15	TS=((needs OR challeng: OR concerns OR problems OR barriers OR fear: OR anxiety OR depression OR perspective: OR experience: OR perception: OR doubt: OR stressor: OR distress: OR view: OR value: OR attitud: OR target: OR priorit: OR goal: OR expectation:) NEAR/2 (patients OR recipients))
16	TS=((needs OR challeng: OR concerns OR problems OR barriers OR fear: OR anxiety OR depression OR perspective: OR experience: OR perception: OR doubt: OR stressor: OR distress: OR view: OR value: OR attitud: OR target: OR priorit: OR goal: OR expectation:) NEAR/4 "transplant patients")
17	TS= ((needs OR challeng: OR concerns OR problems OR barriers OR fear: OR anxiety OR depression OR perspective: OR experience: OR perception: OR doubt: OR stressor: OR distress: OR view: OR value: OR attitud: OR target: OR priorit: OR goal: OR expectation:) NEAR/4 "transplant recipients")
18	#14 OR #15 OR #16 OR #17

#	Search term
19	TS= (SOTx OR SOT)
20	TS= (organ NEAR/2 (graft: OR transplant: OR allograft:))
21	TS=((heart OR heart-lung OR cardiac: OR cardio: OR thorac: OR lung: OR kidney OR renal OR liver: OR hepatic: OR hepato: OR pancrea: OR "small bowel" OR intest:) NEAR/2 (transplant: OR graft: OR allograft:))
22	#19 OR #20 OR #21
23	#18 AND #22
24	ALL=Animals NOT ALL=Humans
25	#23 NOT #24

CINAHL (EBSCO)

Science Citation Index Expanded (SCI-EXPANDED), Conference Proceedings Citation Index – Science (CPCI-S), Emerging Sources Citation Index (ESCI)

#	Search term
S54	S37 AND S53
S53	S45 OR S52
S52	S46 OR S47
S51	S37 AND S50
S50	S45 OR S49
S49	S46 OR S47 OR S48
S48	(((TI heart OR AB heart OR SU heart) OR (TI heart-lung OR AB heart-lung OR SU heart-lung) OR (TI cardiac: OR AB cardiac: OR SU cardiac:) OR (TI cardio: OR AB cardio: OR SU cardio:) OR (TI thorac: OR AB thorac: OR SU thorac:) OR (TI lung: OR AB lung: OR SU lung:) OR (TI kidney OR AB kidney OR SU kidney) OR (TI renal OR AB renal OR SU renal) OR (TI liver: OR AB liver: OR SU liver:) OR (TI hepatic: OR AB hepatic: OR SU hepatic:) OR (TI hepato: OR AB hepato: OR SU hepato:) OR (TI pancrea: OR AB pancrea: OR SU pancrea:) OR (TI "small bowel" OR AB "small bowel" OR SU "small bowel") OR (TI intest: OR AB intest: OR SU intest:)) N2 ((TI transplant: OR AB transplant: OR SU transplant:) OR (TI graft: OR AB graft: OR SU graft:) OR (TI allograft: OR AB allograft: OR SU allograft:)))
S47	((TI organ OR AB organ OR SU organ) N2 ((TI graft: OR AB graft: OR SU graft:) OR (TI transplant: OR AB transplant: OR SU transplant:) OR (TI allograft: OR AB allograft: OR SU allograft:)))
S46	((TI SOTx OR AB SOTx OR SU SOTx) OR (TI SOT OR AB SOT OR SU SOT))
S45	S37 OR S38 OR S39 OR S40 OR S41 OR S42 OR S43 OR S44
S44	(MH "Transplant Recipients") OR (MH "Organ Transplantation")
S43	(MH "Intestine, Small+/TR") OR (MH "Ileum/TR")
S42	(MH "Pancreas Transplantation")
S41	(MH "Heart Transplantation"+)
S40	(MH "Liver Transplantation")
S39	(MH "Lung Transplantation")

#	Search term
S38	(MH "Kidney Transplantation")
S37	S25 OR S36
S36	(S31 OR S35)
S35	S32 OR S33 OR S34
S34	(((TI needs OR AB needs OR SU needs) OR (TI challeng: OR AB challeng: OR SU challeng:) OR (TI concerns OR AB concerns OR SU concerns) OR (TI problems OR AB problems OR SU problems) OR (TI barriers OR AB barriers OR SU barriers) OR (TI fear: OR AB fear: OR SU fear:) OR (TI anxiety OR AB anxiety OR SU anxiety) OR (TI depression OR AB depression OR SU depression) OR (TI perspective: OR AB perspective: OR SU perspective:) OR (TI experience: OR AB experience: OR SU experience:) OR (TI perception: OR AB perception: OR SU perception:) OR (TI doubt: OR AB doubt: OR SU doubt:) OR (TI stressor: OR AB stressor: OR SU stressor:) OR (TI distress: OR AB distress: OR SU distress:) OR (TI view: OR AB view: OR SU view:) OR (TI value: OR AB value: OR SU value:) OR (TI attitud: OR AB attitud: OR SU attitud:) OR (TI target: OR AB target: OR SU target:) OR (TI priorit: OR AB priorit: OR SU priorit:) OR (TI goal: OR AB goal: OR SU goal:) OR (TI expectation: OR AB expectation: OR SU expectation:)) N4 (TI "transplant recipients" OR AB "transplant recipients" OR SU "transplant recipients"))
S33	(((TI needs OR AB needs OR SU needs) OR (TI challeng: OR AB challeng: OR SU challeng:) OR (TI concerns OR AB concerns OR SU concerns) OR (TI problems OR AB problems OR SU problems) OR (TI barriers OR AB barriers OR SU barriers) OR (TI fear: OR AB fear: OR SU fear:) OR (TI anxiety OR AB anxiety OR SU anxiety) OR (TI depression OR AB depression OR SU depression) OR (TI perspective: OR AB perspective: OR SU perspective:) OR (TI experience: OR AB experience: OR SU experience:) OR (TI perception: OR AB perception: OR SU perception:) OR (TI doubt: OR AB doubt: OR SU doubt:) OR (TI stressor: OR AB stressor: OR SU stressor:) OR (TI distress: OR AB distress: OR SU distress:) OR (TI view: OR AB view: OR SU view:) OR (TI value: OR AB value: OR SU value:) OR (TI attitud: OR AB attitud: OR SU attitud:) OR (TI target: OR AB target: OR SU target:) OR (TI priorit: OR AB priorit: OR SU priorit:) OR (TI goal: OR AB goal: OR SU goal:) OR (TI expectation: OR AB expectation: OR SU expectation:)) N4 (TI "transplant patients" OR AB "transplant patients" OR SU "transplant patients"))
S32	(((TI needs OR AB needs OR SU needs) OR (TI challeng: OR AB challeng: OR SU challeng:) OR (TI concerns OR AB concerns OR SU concerns) OR (TI problems OR AB problems OR SU problems) OR (TI barriers OR AB barriers OR SU barriers) OR (TI fear: OR AB fear: OR SU fear:) OR (TI anxiety OR AB anxiety OR SU anxiety) OR (TI depression OR AB depression OR SU depression) OR (TI perspective: OR AB perspective: OR SU perspective:) OR (TI experience: OR AB experience: OR SU experience:) OR (TI perception: OR AB perception: OR SU perception:) OR (TI doubt: OR AB doubt: OR SU doubt:) OR (TI stressor: OR AB stressor: OR SU stressor:) OR (TI distress: OR AB distress: OR SU distress:) OR (TI view: OR AB view: OR SU view:) OR (TI value: OR AB value: OR SU value:) OR (TI attitud: OR AB attitud: OR SU attitud:) OR (TI target: OR AB target: OR SU target:) OR (TI priorit: OR AB priorit: OR SU priorit:) OR (TI goal: OR AB goal: OR SU goal:) OR (TI expectation: OR AB expectation: OR SU expectation:)) N2 ((TI patients OR AB patients OR SU patients) OR (TI recipients OR AB recipients OR SU recipients)))
S31	S26 OR S27 OR S28 OR S29 OR S30
S30	(MH "Depression")
S29	(MH "Anxiety")

#	Search term
S28	(MH "Patient Satisfaction")
S27	(MH "Body Image")
S26	(MH "Needs Assessment")
S25	S12 OR S24
S24	S13 OR S14 OR S15 OR S16 OR S17 OR S18 OR S19 OR S20 OR S21 OR S22 OR S23
S23	(TI "health literacy" OR AB "health literacy" OR SU "health literacy")
S22	(TI "health manag:" OR AB "health manag:" OR SU "health manag:")
S21	((TI cope OR AB cope OR SU cope) OR (TI coping OR AB coping OR SU coping))
S20	((TI "life style" OR AB "life style" OR SU "life style") OR (TI lifestyle OR AB lifestyle OR SU lifestyle))
S19	((TI symptom OR AB symptom OR SU symptom) N2 (TI manag: OR AB manag: OR SU manag:))
S18	(TI "self efficac:" OR AB "self efficac:" OR SU "self efficac:")
S17	(TI "self monitor:" OR AB "self monitor:" OR SU "self monitor:")
S16	(TI "self regulat:" OR AB "self regulat:" OR SU "self regulat:")
S15	(TI "self help:" OR AB "self help:" OR SU "self help:")
S14	(TI "self car:" OR AB "self car:" OR SU "self car:")
S13	(TI "self manag:" OR AB "self manag:" OR SU "self manag:")
S12	S1 OR S2 OR S3 OR S4 OR S5 OR S6 OR S7 OR S8 OR S9 OR S10 OR S11
S11	(MH "Adaptation, Psychological")
S10	(MH "Activities of Daily Living+")
S9	(MH "Health Behavior")
S8	(MH "Life Change Events")
S7	(MH "Life Style")
S6	(MH "Health Literacy")
S5	(MH "Self-Efficacy")
S4	(MH "Self Administration")
S3	(MH "Support Groups")
S2	(MH Self-Management)
S1	(MH "Self Care")

PsycInfo (EBSCO)

#	Search term
S97	S96 NOT (PO Animal NOT PO Human)
S96	S94 AND S95
S95	S78 OR S90
S94	S91 OR S92 OR S93
S93	TI (((heart or heart-lung or cardiac: or cardio: or thorac: or lung: or kidney or renal or liver: or hepatic: or hepato: or pancrea: or small bowel or intest:) n2 (transplant: or graft: or allograft:))) OR AB (((heart or heart-lung or cardiac: or cardio: or thorac: or lung: or kidney or renal or liver: or hepatic: or hepato: or pancrea: or small bowel or intest:) n2 (transplant: or graft: or allograft:))) OR KW (((heart or heart-lung

#	Search term
	or cardiac: or cardio: or thorac: or lung: or kidney or renal or liver: or hepatic: or hepato: or pancrea: or small bowel or intest:) n2 (transplant: or graft: or allograft:)))
S92	TI (organ n2 (graft: or transplant: or allograft:)) OR AB (organ n2 (graft: or transplant: or allograft:)) OR KW (organ n2 (graft: or transplant: or allograft:))
S91	DE "Organ Transplantation"
S90	S85 OR S89
S89	S86 OR S87 OR S88
S88	TI (((needs or challeng: or concerns or problems or barriers or fear: or anxiety or depression or perspective: or experience: or perception: or doubt: or stressor: or distress: or view: or value: or attitud: or target: or priorit: or goal: or expectation:) n4 transplant recipients) OR AB (((needs or challeng: or concerns or problems or barriers or fear: or anxiety or depression or perspective: or experience: or perception: or doubt: or stressor: or distress: or view: or value: or attitud: or target: or priorit: or goal: or expectation:) n4 transplant recipients) OR KW ((((needs or challeng: or concerns or problems or barriers or fear: or anxiety or depression or perspective: or experience: or perception: or doubt: or stressor: or distress: or view: or value: or attitud: or target: or priorit: or goal: or expectation:) n4 transplant recipients)
S87	TI (((needs or challeng: or concerns or problems or barriers or fear: or anxiety or depression or perspective: or experience: or perception: or doubt: or stressor: or distress: or view: or value: or attitud: or target: or priorit: or goal: or expectation:) n4 transplant patients).) OR AB (((needs or challeng: or concerns or problems or barriers or fear: or anxiety or depression or perspective: or experience: or perception: or doubt: or stressor: or distress: or view: or value: or attitud: or target: or priorit: or goal: or expectation:) n4 transplant patients).) OR KW (((needs or challeng: or concerns or problems or barriers or fear: or anxiety or depression or perspective: or experience: or perception: or doubt: or stressor: or distress: or view: or value: or attitud: or target: or priorit: or goal: or expectation:) n4 transplant patients).)
S86	TI (((needs or challeng: or concerns or problems or barriers or fear: or anxiety or depression or perspective: or experience: or perception: or doubt: or stressor: or distress: or view: or value: or attitud: or target: or priorit: or goal: or expectation:) n2 (patients or recipients)).) OR AB (((needs or challeng: or concerns or problems or barriers or fear: or anxiety or depression or perspective: or experience: or perception: or doubt: or stressor: or distress: or view: or value: or attitud: or target: or priorit: or goal: or expectation:) n2 (patients or recipients)) OR KW (((needs or challeng: or concerns or problems or barriers or fear: or anxiety or depression or perspective: or experience: or perception: or doubt: or stressor: or distress: or view: or value: or attitud: or target: or priorit: or goal: or expectation:) n2 (patients or recipients))
S85	S79 OR S80 OR S81 OR S82 OR S83 OR S84
S84	DE "Depression (Emotion)"
S83	DE "Anxiety"
S82	DE "Client Satisfaction"
S81	DE "Body Image"
S80	DE "Self-Perception"
S79	DE "Needs Assessment"
S78	S65 OR S77

#	**Search term**
S77	S66 OR S67 OR S68 OR S69 OR S70 OR S71 OR S72 OR S73 OR S74 OR S75 OR S76
S76	TI "health literacy" OR AB "health literacy" OR KW "health literacy"
S75	TI "health manag:" OR AB "health manag:" OR KW "health manag:"
S74	TI (cope or coping) OR AB (cope or coping) OR KW (cope or coping)
S73	TI ((life style or lifestyle)) OR AB ((life style or lifestyle)) OR KW ((life style or lifestyle))
S72	TI symptom n2 manag: OR AB symptom n2 manag: OR KW symptom n2 manag:
S71	TI "self efficac:" OR AB "self efficac:" OR KW "self efficac:"
S70	TI "self monitor:" OR AB "self monitor:" OR KW "self monitor:"
S69	TI "self regulat:" OR AB "self regulat:" OR KW "self regulat:"
S68	TI "self help:" OR AB "self help:" OR KW "self help:"
S67	TI "self car:" OR AB "self car:" OR KW "self car:"
S66	TI "self manag:" OR AB "self manag:" OR KW "self manag:"
S65	S55 OR S56 OR S57 OR S58 OR S59 OR S60 OR S61 OR S62 OR S63 OR S64
S64	DE "Health Behavior"
S63	DE "Adaptive Behavior"
S62	DE "Activities of Daily Living"
S61	DE "Lifestyle"
S60	DE "Health Literacy"
S59	DE "Self-Efficacy"
S58	DE "Self-Help Techniques"
S57	DE "Support Groups"
S56	DE "Self-Management"
S55	DE "Self-Care"
S54	S37 AND S53
S53	S45 OR S52
S52	S46 OR S47
S51	S37 AND S50
S50	S45 OR S49
S49	S46 OR S47 OR S48
S48	(((TI heart OR AB heart OR SU heart) OR (TI heart-lung OR AB heart-lung OR SU heart-lung) OR (TI cardiac: OR AB cardiac: OR SU cardiac:) OR (TI cardio: OR AB cardio: OR SU cardio:) OR (TI thorac: OR AB thorac: OR SU thorac:) OR (TI lung: OR AB lung: OR SU lung:) OR (TI kidney OR AB kidney OR SU kidney) OR (TI renal OR AB renal OR SU renal) OR (TI liver: OR AB liver: OR SU liver:) OR (TI hepatic: OR AB hepatic: OR SU hepatic:) OR (TI hepato: OR AB hepato: OR SU hepato:) OR (TI pancrea: OR AB pancrea: OR SU pancrea:) OR (TI "small bowel" OR AB "small bowel" OR SU "small bowel") OR (TI intest: OR AB intest: OR SU intest:)) N2 ((TI transplant: OR AB transplant: OR SU transplant:) OR (TI graft: OR AB graft: OR SU graft:) OR (TI allograft: OR AB allograft: OR SU allograft:)))
S47	((TI organ OR AB organ OR SU organ) N2 ((TI graft: OR AB graft: OR SU graft:) OR (TI transplant: OR AB transplant: OR SU transplant:) OR (TI allograft: OR AB allograft: OR SU allograft:)))

#	Search term
S46	((TI SOTx OR AB SOTx OR SU SOTx) OR (TI SOT OR AB SOT OR SU SOT))
S45	S37 OR S38 OR S39 OR S40 OR S41 OR S42 OR S43 OR S44
S44	(MH "Transplant Recipients") OR (MH "Organ Transplantation")
S43	(MH "Intestine, Small+/TR") OR (MH "Ileum/TR")
S42	(MH "Pancreas Transplantation")
S41	(MH "Heart Transplantation"+)
S40	(MH "Liver Transplantation")
S39	(MH "Lung Transplantation")
S38	(MH "Kidney Transplantation")
S37	S25 OR S36
S36	(S31 OR S35)
S35	S32 OR S33 OR S34
S34	(((TI needs OR AB needs OR SU needs) OR (TI challeng: OR AB challeng: OR SU challeng:) OR (TI concerns OR AB concerns OR SU concerns) OR (TI problems OR AB problems OR SU problems) OR (TI barriers OR AB barriers OR SU barriers) OR (TI fear: OR AB fear: OR SU fear:) OR (TI anxiety OR AB anxiety OR SU anxiety) OR (TI depression OR AB depression OR SU depression) OR (TI perspective: OR AB perspective: OR SU perspective:) OR (TI experience: OR AB experience: OR SU experience:) OR (TI perception: OR AB perception: OR SU perception:) OR (TI doubt: OR AB doubt: OR SU doubt:) OR (TI stressor: OR AB stressor: OR SU stressor:) OR (TI distress: OR AB distress: OR SU distress:) OR (TI view: OR AB view: OR SU view:) OR (TI value: OR AB value: OR SU value:) OR (TI attitud: OR AB attitud: OR SU attitud:) OR (TI target: OR AB target: OR SU target:) OR (TI priorit: OR AB priorit: OR SU priorit:) OR (TI goal: OR AB goal: OR SU goal:) OR (TI expectation: OR AB expectation: OR SU expectation:)) N4 (TI "transplant recipients" OR AB "transplant recipients" OR SU "transplant recipients"))
S33	(((TI needs OR AB needs OR SU needs) OR (TI challeng: OR AB challeng: OR SU challeng:) OR (TI concerns OR AB concerns OR SU concerns) OR (TI problems OR AB problems OR SU problems) OR (TI barriers OR AB barriers OR SU barriers) OR (TI fear: OR AB fear: OR SU fear:) OR (TI anxiety OR AB anxiety OR SU anxiety) OR (TI depression OR AB depression OR SU depression) OR (TI perspective: OR AB perspective: OR SU perspective:) OR (TI experience: OR AB experience: OR SU experience:) OR (TI perception: OR AB perception: OR SU perception:) OR (TI doubt: OR AB doubt: OR SU doubt:) OR (TI stressor: OR AB stressor: OR SU stressor:) OR (TI distress: OR AB distress: OR SU distress:) OR (TI view: OR AB view: OR SU view:) OR (TI value: OR AB value: OR SU value:) OR (TI attitud: OR AB attitud: OR SU attitud:) OR (TI target: OR AB target: OR SU target:) OR (TI priorit: OR AB priorit: OR SU priorit:) OR (TI goal: OR AB goal: OR SU goal:) OR (TI expectation: OR AB expectation: OR SU expectation:)) N4 (TI "transplant patients" OR AB "transplant patients" OR SU "transplant patients"))
S32	(((TI needs OR AB needs OR SU needs) OR (TI challeng: OR AB challeng: OR SU challeng:) OR (TI concerns OR AB concerns OR SU concerns) OR (TI problems OR AB problems OR SU problems) OR (TI barriers OR AB barriers OR SU barriers) OR (TI fear: OR AB fear: OR SU fear:) OR (TI anxiety OR AB anxiety OR SU anxiety) OR (TI depression OR AB depression OR SU depression) OR (TI perspective: OR AB perspective: OR SU perspective:) OR (TI experience: OR AB experience: OR SU experience:) OR (TI perception: OR AB perception: OR SU perception:) OR (TI doubt: OR AB doubt:

#	**Search term**
	OR SU doubt:) OR (TI stressor: OR AB stressor: OR SU stressor:) OR (TI distress: OR AB distress: OR SU distress:) OR (TI view: OR AB view: OR SU view:) OR (TI value: OR AB value: OR SU value:) OR (TI attitud: OR AB attitud: OR SU attitud:) OR (TI target: OR AB target: OR SU target:) OR (TI priorit: OR AB priorit: OR SU priorit:) OR (TI goal: OR AB goal: OR SU goal:) OR (TI expectation: OR AB expectation: OR SU expectation:)) N2 ((TI patients OR AB patients OR SU patients) OR (TI recipients OR AB recipients OR SU recipients)))
S31	S26 OR S27 OR S28 OR S29 OR S30
S30	(MH "Depression")
S29	(MH "Anxiety")
S28	(MH "Patient Satisfaction")
S27	(MH "Body Image")
S26	(MH "Needs Assessment")
S25	S12 OR S24
S24	S13 OR S14 OR S15 OR S16 OR S17 OR S18 OR S19 OR S20 OR S21 OR S22 OR S23
S23	(TI "health literacy" OR AB "health literacy" OR SU "health literacy")
S22	(TI "health manag:" OR AB "health manag:" OR SU "health manag:")
S21	((TI cope OR AB cope OR SU cope) OR (TI coping OR AB coping OR SU coping))
S20	((TI "life style" OR AB "life style" OR SU "life style") OR (TI lifestyle OR AB lifestyle OR SU lifestyle))
S19	((TI symptom OR AB symptom OR SU symptom) N2 (TI manag: OR AB manag: OR SU manag:))
S18	(TI "self efficac:" OR AB "self efficac:" OR SU "self efficac:")
S17	(TI "self monitor:" OR AB "self monitor:" OR SU "self monitor:")
S16	(TI "self regulat:" OR AB "self regulat:" OR SU "self regulat:")
S15	(TI "self help:" OR AB "self help:" OR SU "self help:")
S14	(TI "self car:" OR AB "self car:" OR SU "self car:")
S13	(TI "self manag:" OR AB "self manag:" OR SU "self manag:")
S12	S1 OR S2 OR S3 OR S4 OR S5 OR S6 OR S7 OR S8 OR S9 OR S10 OR S11
S11	(MH "Adaptation, Psychological")
S10	(MH "Activities of Daily Living+")
S9	(MH "Health Behavior")
S8	(MH "Life Change Events")
S7	(MH "Life Style")
S6	(MH "Health Literacy")
S5	(MH "Self-Efficacy")
S4	(MH "Self Administration")
S3	(MH "Support Groups")
S2	(MH Self-Management)
S1	(MH "Self Care")

ClinicalTrials.gov

Condition or disease

"Organ Transplantation" OR "Kidney Transplantation" OR "Lung Transplantation" OR "Liver Transplantation " OR "Heart Transplantation" OR "Pancreas Transplantation" OR "Transplant Recipient"

Other terms

"self-management" OR "self-care" OR "self-help"

WHO International Clinical Trials Registry Platform

Advanced Search/Condition

"Organ Transplantation" OR "Kidney Transplantation" OR "Lung Transplantation" OR "Liver Transplantation " OR "Heart Transplantation" OR "Pancreas Transplantation" OR "Transplant Recipient"

German Clinical Trials Register (Deutsches Register klinischer Studien [DRKS])

"Organ Transplantation:" OR "Kidney Transplantation:" OR "Lung Transplantation:" OR "Liver Transplantation:" OR "Heart Transplantation:" OR "Pancreas Transplantation:" OR "Transplant Recipient:"
