# Supplementary material for: Aspects of Self-Management After Solid Organ Transplantation—A Scoping Review
Source: Nurs Rep. 2025 Aug 19;15(8):304. doi: 10.3390/nursrep15080304 (PMC12389569; doi:10.3390/nursrep15080304)
Supplement: Supplementary file 1 [file nursrep-15-00304-s001.zip › Supplementary File S3.pdf]

Supplementary File S3: References for all documents included in the scoping review

The references of the documents included in the scoping review for analysis are listed below. Merged references are indented and listed under the respective main reference.

1. Achille, M. A., Ouellette, A., Fournier, S., Hébert, M.-J., Girardin, C., & Pâquet, M. (2004). Impact of transplant-related stressors and feelings of indebtedness on psychosocial adjustment following kidney transplantation. *Journal of Clinical Psychology in Medical Settings*, *11*(1), 63–73. https://doi.org/10.1023/B:JOCS.0000016271.10786.ee
2. Achille, M. A., Ouellette, A., Fournier, S., Vachon, M., & Hebert, M. J. (2006). Impact of stress, distress and feelings of indebtedness on adherence to immunosuppressants following kidney transplantation. *Clinical Transplantation*, *20*(3), 301–306. https://doi.org/10.1111/j.1399-0012.2005.00478.x
3. Ådahl, S. (2013). When death enables life: Incorporation of organs from deceased donors in Finnish kidney recipients. *Mortality*, *18*(2), 130–150. https://doi.org/10.1080/13576275.2013.785504
4. Adhikari, U. R., Taraphder, A., Das, T., & Hazra, A. (2014). An Exploratory Study to Identify Factors Associated with Noncompliance of Medications and Recommended Lifestyle Behavior After Renal Transplantation-A Pilot Study. *International Journal of Nursing Education*, *6*(1), 73. https://doi.org/10.5958/j.0974-9357.6.1.015
5. Adhikari, U. R., Taraphder, A., Hazra, A., & Das, T. (2018). Compliance of kidney transplant recipients to the recommended lifestyle measures following transplantation. *Indian Journal of Transplantation*, *12*(1), 17–24. https://doi.org/10.4103/ijot.ijot_55_17
6. Adrichem, E. J. van, Dekker, R., Krijnen, W. P., Verschuuren, E. A. M., Dijkstra, P. U., & Schans, C. P. van der. (2018). Physical Activity, Sedentary Time, and Associated Factors in Recipients of Solid-Organ Transplantation. *Physical Therapy*, *98*(8), 646–657. https://doi.org/10.1093/ptj/pzy055
7. Aghakhani, N., Maslakpak, M. H., Jalali, S., & Parizad, N. (2021). Self-Care Education Program as a New Pathway Toward Improving Quality of Life in Kidney Transplant Patients: A Single-Blind, Randomized, Controlled Trial. *Experimental and Clinical Transplantation: Official Journal of the Middle East Society for Organ Transplantation*, *19*(3), 224–230. https://doi.org/10.6002/ect.2020.0044
    - Aghakhani, N. (2018). *The effect of self-care education on the quality of life in renal transplant patients referring to Imam reza hospital in Tabriz* (Clinical trial registration IRCT20160220026662N3). Iranian Registry of Clinical trials (IRCT). https://www.irct.ir/trial/33308
8. Akazawa, C., Nishizono, T., Yamamoto, M., Teraguchi, S., & Hayashi, Y. (2013). Investigation of actual daily lifestyle leading to continuous self-management after living-donor liver transplantation: More than 5 years living with living-donor liver transplantation and emotions of recipients. *Japan Journal of Nursing Science: JJNS*, *10*(1), 79–88. https://doi.org/10.1111/j.1742-7924.2012.00214.x
9. Akin, S., & Harmanci Seren, A. K. (2019). Care of Elderly Renal Recipients. *International Journal of Caring Sciences*, *12*(2), 1272–1278.
10. Akyolcu, N. (2002). Patient education in renal transplantation. *Journal of Renal Care*, *28*(4), 176–179. https://doi.org/10.1111/j.1755-6686.2002.tb00240.x
11. Al-Aradi, A., Phelan, P. J., O'Kelly, P., Khan, A. H., Rahman, M. A., Hanley, A., Ho, C., Kheradmand, F., Hickey, D., Spencer, S., Magee, C., Walshe, J. J., Morgan, N., & Conlon, P. J. (2009). An assessment of the long-term health outcome of renal transplant recipients in Ireland. *Irish Journal of Medical Science*, *178*(4), 407–412. https://doi.org/10.1007/s11845-009-0363-z

12. Almgren, M., Lennerling, A., Lundmark, M., & Forsberg, A. (2017a). Self-efficacy in the context of heart transplantation—A new perspective. *Journal of Clinical Nursing*, *26*(19–20), 3007–3017. https://doi.org/10.1111/jocn.13647
13. Almgren, M., Lennerling, A., Lundmark, M., & Forsberg, A. (2017b). The meaning of being in uncertainty after heart transplantation—An unrevealed source to distress. *European Journal of Cardiovascular Nursing*, *16*(2), 167–174. https://doi.org/10.1177/1474515116648240
14. Almgren, M., Lundqvist, P., Lennerling, A., & Forsberg, A. (2020). Fatigue after heart transplantation—A possible barrier to self-efficacy. *Scandinavian Journal of Caring Sciences*. https://doi.org/10.1111/scs.12951
15. Almgren, M., Lundqvist, P., Lennerling, A., & Forsberg, A. (2021). Self-efficacy, recovery and psychological wellbeing one to five years after heart transplantation: A Swedish cross-sectional study. *European Journal of Cardiovascular Nursing*, *20*(1), 34–39. https://doi.org/10.1177/1474515120927121
16. Amerena, P., & Wallace, P. (2009). Psychological experiences of renal transplant patients: A qualitative analysis. *Counselling and Psychotherapy Research*, *9*(4), 273–279. https://doi.org/10.1080/14733140902935195
17. Anastacio, L. R., Ferreira, L. G., de Sena Ribeiro, H., Lima, A. S., Vilela, E. G., & Toulson Davisson Correia, M. I. (2011). Body composition and overweight of liver transplant recipients. *Transplantation*, *92*(8), 947–951. https://doi.org/10.1097/TP.0b013e31822e0bee
18. Andersen, M. H., Wahl, A. K., Engebretsen, E., & Urstad, K. H. (2019). Implementing a tailored education programme: Renal transplant recipients' experiences. *Journal of Renal Care*, *45*(2), 111–119. https://doi.org/10.1111/jorc.12273
19. Annette, L., Annika, K., & Anna, F. (2019). Non-adherence to immunosuppressant after lung transplantation—A common risk behavior. *The Open Nursing Journal*, *13*(1). https://doi.org/10.2174/1874434601913010108
20. Anupama, S., Abraham, G., Parthasarathy, R., Anupama, P., & Mathew, M. (2020). Malnutrition in kidney transplantation: Our experience and review of literature. *Indian Journal of Transplantation*, *14*(1), 15–18. https://doi.org/10.4103/ijot.ijot_40_19
21. Arakawa, C., Teraguchi, S., Akazawa, C., Nishizono, T., & Yamamoto, M. (2014). Self-management of infection control behavior of adult recipients of living-donor liver transplantation within 5 years after transplantation. *Transplantation Proceedings*, *46*(3), 838–840. https://doi.org/10.1016/j.transproceed.2013.12.044
22. Arruda, G. O. de, & Renovato, R. D. (2012). Drug utilization in renal transplant patients: Medication practices and representations. *Revista Gaúcha de Enfermagem*, *33*, 157–164. https://doi.org/10.1590/S1983-14472012000400020
23. Asavakarn, S., Sirivatanauksorn, Y., Promraj, R., Ruenrom, A., Limsrichamrern, S., Kositamongkol, P., Mahawithitwong, P., Tovikkai, C., & Dumronggittigule, W. (2016). Systematic Pharmaceutical Educational Approach to Enhance Drug Adherence in Liver Transplant Recipients. *Transplantation Proceedings*, *48*(4), 1202–1207. https://doi.org/10.1016/j.transproceed.2015.12.100
24. Avery, R. K., Michaels, M. G., & A. S. T. Infectious Diseases Community of Practice. (2019). Strategies for safe living following solid organ transplantation-Guidelines from the American Society of Transplantation Infectious Diseases Community of Practice. *Clinical Transplantation*, *33*(9), e13519. https://doi.org/10.1111/ctr.13519
25. Baas, L. S., Bell, B., Giesting, R., McGuire, N., & Wagoner, L. E. (2003). Infections in the heart transplant recipient. *Critical Care Nursing Clinics of North America*, *15*(1), 97–108. https://doi.org/10.1016/S0899-5885(02)00035-7
26. Bahruth, A. J. (2004). What every patient should know...pretransplantation and posttransplantation. *Critical Care Nursing Quarterly*, *27*(1), 31–60. https://doi.org/10.1097/00002727-200401000-00004
27. Baines, L. S., & Jindal, R. M. (2002). Loss of the imagined past: An emotional obstacle to medical compliance in kidney transplant recipients. *Progress in Transplantation*, *12*(4), 305–308. https://doi.org/10.7182/prtr.12.4.x7451j81675l7022

28. Baines, L. S., Joseph, J. T., & Jindal, R. M. (2002). Compliance and late acute rejection after kidney transplantation: A psycho-medical perspective. *Clinical Transplantation*, *16*(1), 69–73. https://doi.org/10.1034/j.1399-0012.2002.00111.x
29. Baldoni, L., De Simone, P., Paganelli, R., Traballoni, L., Elisei, M., Bindi, L., Ducci, J., Carrai, P., Bisa, M., Coletti, L., Petruccelli, S., Masetti, M., Padovan, A., Coscetti, F., & Filipponi, F. (2008). The „You Are Not Alone“ care program for liver transplantation. *Transplantation Proceedings*, *40*(6), 1983–1985. https://doi.org/10.1016/j.transproceed.2008.05.066
30. Baran, A. (2013). The role of resiliency in the process of adaptation to life after heart transplantation. *Archives of Psychiatry and Psychotherapy*, *15*(2), 45–52. https://doi.org/10.12740/APP/17372
31. Bardet, J. D., Charpiat, B., Rebillon, M., Bedouch, P., Tourette-Turgis, C., & Allenet, B. (2013). Beliefs in liver transplantation: An exploratory qualitative study based on the self-regulation model in 1 year or more liver-transplanted patients. *International Journal of Clinical Pharmacy*, *35*(5), 913–913.
32. Barnett, A., Campbell, K. L., Mayr, H. L., Keating, S. E., Macdonald, G. A., & Hickman, I. J. (2021). Liver transplant recipients' experiences and perspectives of a telehealth-delivered lifestyle programme: A qualitative study. *Journal of Telemedicine and Telecare*, *27*(9), 590–598. https://doi.org/10.1177/1357633x19900459
33. Barros, L. B. F., Silva, L. F. D., Guedes, M. V. C., & Pessoa, V. (2017). Clinical care of nursing reasoned in Parse: Contribution in the transcendence process of cardiac transplantation. *Revista Gaúcha de Enfermagem*, *38*(2), e60658. https://doi.org/10.1590/1983-1447.2017.02.60658
34. Bass, M., Galley-Reilley, J., Twiss, D. E., & Whitaker, D. (1999). A diversified patient education program for transplant recipients. *ANNA Journal*, *26*(3), 287–292, 343.
35. Basset Seguin, N., Malvehy, J., Nadal, F., Creancier, L., Rauly-Lestienne, I., Beauchamp, R., Hezareh, M., Schmitt, A. M., & Ulianov, L. (2019). Risk behaviour and patient preferences for an improved non-melanoma skin cancer prevention modality for organ-transplanted patients: A European, multi-country, online patient community study. *European Journal of Dermatology*, *29*(5), 518–523. https://doi.org/10.1684/ejd.2019.3639
36. Baumann, L. J., Young, C. J., & Egan, J. J. (1992). Living with a heart transplant: Long-term adjustment. *Transplant International*, *5*(1), 1–8. https://doi.org/10.1007/BF00337181
37. Beck, D., Been-Dahmen, J., Peeters, M., Grijpma, J. W., van der Stege, H., Tielen, M., van Buren, M., Weimar, W., Ista, E., Massey, E., & van Staa, A. (2019). A Nurse-Led Self-Management Support Intervention (ZENN) for Kidney Transplant Recipients Using Intervention Mapping: Protocol for a Mixed-Methods Feasibility Study. *JMIR Research Protocols*, *8*(3), e11856. https://doi.org/10.2196/11856
38. Beckmann, S., Künzler-Heule, P., Biotti, B., & Spirig, R. (2016). Mastering Together the Highs and Lows: Patients' and Caregivers' Perceptions of Self-Management in the Course of Liver Transplantation. *Progress in Transplantation*, *26*(3), 215–223. https://doi.org/10.1177/1526924816654769
39. Beckmann, S., Nikolic, N., Denhaerynck, K., Binet, I., Koller, M., Boely, E., De Geest, S., & Psychosocial Interest Group, S. T. C. S. (2017). Evolution of body weight parameters up to 3 years after solid organ transplantation: The prospective Swiss Transplant Cohort Study. *Clinical Transplantation*, *31*(3). https://doi.org/10.1111/ctr.12896
40. Beekman, L., Berzigotti, A., & Banz, V. (2018). Physical Activity in Liver Transplantation: A Patient's and Physicians' Experience. *Advances in Therapy*, *35*(11), 1729–1734. https://doi.org/10.1007/s12325-018-0797-7
41. Been-Dahmen, J. M. J., Beck, D. K., Peeters, M. A. C., van der Stege, H., Tielen, M., van Buren, M. C., Ista, E., van Staa, A., & Massey, E. K. (2019). Evaluating the feasibility of a nurse-led self-management support intervention for kidney transplant

recipients: A pilot study. *BMC Nephrology*, *20*(1), 143. https://doi.org/10.1186/s12882-019-1300-7
42. Been-Dahmen, J. M. J., Grijpma, J. W., Ista, E., Dwarswaard, J., Maasdam, L., Weimar, W., Van Staa, A., & Massey, E. K. (2018). Self-management challenges and support needs among kidney transplant recipients: A qualitative study. *Journal of Advanced Nursing*, *74*(10), 2393–2405. https://doi.org/10.1111/jan.13730
43. Belaiche, S., Decaudin, B., Dharancy, S., Noel, C., Odou, P., & Hazzan, M. (2017). Factors relevant to medication non-adherence in kidney transplant: A systematic review. *International Journal of Clinical Pharmacy*, *39*(3), 582–593. https://doi.org/10.1007/s11096-017-0436-4
44. Berben, L., Denhaerynck, K., Dobbels, F., Engberg, S., Vanhaecke, J., Crespo-Leiro, M. G., Russell, C. L., De Geest, S., & Bright study consortium. (2015). Building research initiative group: Chronic illness management and adherence in transplantation (BRIGHT) study: Study protocol. *Journal of Advanced Nursing*, *71*(3), 642–654. https://doi.org/10.1111/jan.12519
    - De Geest, S. (2012). *Building Research Initiative Group: Chronic Illness Management and Adherence in Transplantation* (Clinical trial registration NCT01608477). clinicaltrials.gov. https://clinicaltrials.gov/ct2/show/NCT01608477
45. Berben, L., Dobbels, F., Kugler, C., Russell, C. L., & De Geest, S. (2011). Interventions used by health care professionals to enhance medication adherence in transplant patients: A survey of current clinical practice. *Progress in Transplantation*, *21*(4), 322–331. https://doi.org/10.1177/152692481102100412
46. Bertram, A., Pabst, S., Zimmermann, T., Schiffer, M., & de Zwaan, M. (2016). How can you be adherent if you don’t know how? *Transplant International*, *29*(7), 830–832. https://doi.org/10.1111/tri.12784
47. Bissonnette, J., Woodend, K., Davies, B., Stacey, D., & Knoll, G. A. (2013). Evaluation of a collaborative chronic care approach to improve outcomes in kidney transplant recipients. *Clinical Transplantation*, *27*(2), 232–238. https://doi.org/10.1111/ctr.12068
48. Blanchard, W. A. (1998). Teaching an illiterate transplant patient. *ANNA Journal*, *25*(1), 69–70, 76.
49. Blumenthal, J. A., Smith, P. J., Sherwood, A., Mabe, S., Snyder, L., Frankel, C., McKee, D. C., Hamilton, N., Keefe, F. J., Shearer, S., Schwartz, J., & Palmer, S. (2020). Remote Therapy to Improve Outcomes in Lung Transplant Recipients: Design of the INSPIRE-III Randomized Clinical Trial. *Transplantation Direct*, *6*(3), e535. https://doi.org/10.1097/TXD.0000000000000979
    - Duke University. (2019). *Improving Lung Transplant Outcomes With Coping Skills and Physical Activity* (Clinical trial registration NCT04093869). clinicaltrials.gov. https://clinicaltrials.gov/ct2/show/NCT04093869
50. Boaz, A., & Morgan, M. (2014). Working to establish ‘normality’ post-transplant: A qualitative study of kidney transplant patients. *Chronic Illness*, *10*(4), 247–258. https://doi.org/10.1177/1742395313504789
51. Boslooper-Meulenbelt, K., Patijn, O., Battjes-Fries, M. C. E., Haisma, H., Pot, G. K., & Navis, G. J. (2019). Barriers and Facilitators of Fruit and Vegetable Consumption in Renal Transplant Recipients, Family Members and Healthcare Professionals—A Focus Group Study. *Nutrients*, *11*(10), 2427–2427. https://doi.org/10.3390/nu11102427
52. Bosma, O. H., Vermeulen, K. M., Verschuuren, E. A., Erasmus, M. E., & van der Bij, W. (2011). Adherence to immunosuppression in adult lung transplant recipients: Prevalence and risk factors. *Journal of Heart & Lung Transplantation*, *30*(11), 1275–1280. https://doi.org/10.1016/j.healun.2011.05.007
53. Bossenbroek, L., den Ouden, M. E. M., de Greef, M. H. G., Douma, W. R., ten Hacken, N. H. T., & van der Bij, W. (2011). Determinants of Overweight and Obesity in Lung Transplant Recipients. *Respiration*, *82*(1), 28–35. https://doi.org/10.1159/000322833
54. Brito, D. C., Marsicano, E. O., Grincenkov, F. R., Colugnati, F. A., Lucchetti, G., & Sanders-Pinheiro, H. (2016). Stress, coping and adherence to immunosuppressive

medications in kidney transplantation: A comparative study. *Sao Paulo Medical Journal*, *134*(4), 292–299. https://dx.doi.org/10.1590/1516-3180.2015.01071008
55. Brocks, Y., Zittermann, A., Grisse, D., Schmid-Ott, G., Stock-Giesendanner, S., Schulz, U., Brakhage, J., Benkler, A., Gummert, J., & Tigges-Limmer, K. (2017). Adherence of Heart Transplant Recipients to Prescribed Medication and Recommended Lifestyle Habits. *Progress in Transplantation*, *27*(2), 160–166. https://doi.org/10.1177/1526924817699959
56. Browning, R. B., McGillicuddy, J. W., Treiber, F. A., & Taber, D. J. (2016). Kidney transplant recipients' attitudes about using mobile health technology for managing and monitoring medication therapy. *Journal of the American Pharmacists Association*, *56*(4), 450-454.e1. https://doi.org/10.1016/j.japh.2016.03.017
57. Bühler, M. N., Feldmeyer, L., Wüthrich, R. P., French, L. E., Djamei, V., Serra, A. L., & Hofbauer, G. F. (2013). [The educational website Dermaguard to prevent the incidence of skin cancer after transplantation]. *Praxis*, *102*(23), 1415–1420. https://doi.org/10.1024/1661-8157/a001477
58. Buldukoglu, K., Kulakac, O., Kececioglu, N., Alkan, S., Yilmaz, M., & Yucetin, L. (2005). Recipients' perceptions of their transplanted kidneys. *Transplantation*, *80*(4), 471–476. https://doi.org/10.1097/01.tp.0000168149.95310.6e
59. Bulum, T., Prkacin, I., & Duvnjak, L. (2021). New-Onset Diabetes after Kidney Transplantation: Diagnosis, Risk Factors, and Management. *Acta Clinica Croatica*, *60*, 86–95. https://doi.org/10.20471/acc.2021.60.s1.13
60. Bunzel, B. (1995). [Heart transplantation: Psychosocial correlations in the postoperative period]. *Praxis*, *84*(31–32), 866–871.
61. Bunzel, B., Wollenek, G., & Grundbock, A. (1992a). Living with a donor heart: Feelings and attitudes of patients toward the donor and the donor organ. *Journal of Heart & Lung Transplantation*, *11*(6), 1151–1155.
62. Bunzel, B., Wollenek, G., & Grundbock, A. (1992b). Psychosocial problems of donor heart recipients adversely affecting quality of life. *Quality of Life Research*, *1*(5), 307–313. https://doi.org/10.1007/BF00434944
63. Burra, P., Germani, G., Gnoato, F., Lazzaro, S., Russo, F. P., Cillo, U., & Senzolo, M. (2011). Adherence in liver transplant recipients. *Liver Transplantation*, *17*(7), 760–770. https://doi.org/10.1002/lt.22294
64. Butler, J. A., Peveler, R. C., Roderick, P., Smith, P. W., Horne, R., & Mason, J. C. (2004). Modifiable risk factors for non-adherence to immunosuppressants in renal transplant recipients: A cross-sectional study. *Nephrology Dialysis Transplantation*, *19*(12), 3144–3149. https://doi.org/10.1093/ndt/gfh505
65. Cajita, M. I., Denhaerynck, K., Dobbels, F., Berben, L., Russell, C. L., Davidson, P. M., De Geest, S., & BRIGHT study team. (2017). Health literacy in heart transplantation: Prevalence, correlates and associations with health behaviors-Findings from the international BRIGHT study. *The Journal of Heart and Lung Transplantation*, *36*(3), 272–279. https://doi.org/10.1016/j.healun.2016.08.024
66. Calia, R., Lai, C., Aceto, P., Luciani, M., Camardese, G., Lai, S., Fantozzi, C., Pietroni, V., Salerno, M. P., Spagnoletti, G., Pedroso, J. A., Romagnoli, J., & Citterio, F. (2015). Emotional self-efficacy and alexithymia may affect compliance, renal function and quality of life in kidney transplant recipients: Results from a preliminary cross-sectional study. *Physiology & Behavior*, *142*, 152–154. https://doi.org/10.1016/j.physbeh.2015.02.018
67. Cavallini, J., Forsberg, A., & Lennerling, A. (2015). Social function after solid organ transplantation: An integrative review. *Nordic Journal of Nursing Research*, *35*(4), 227–234. https://doi.org/10.1177/0107408315592335
68. Cebeci, F., Cetin, C., Catal, E., & Bayezid, O. (2021). Life experiences of adult heart transplant recipients: A new life, challenges, and coping. *Quality of Life Research*, *30*(6), 1619–1627. https://doi.org/10.1007/s11136-021-02763-y
69. Çetin, Ç., & Cebeci, F. (2018). Effects of Web-Based Educational Intervention on Self-Management in Kidney Recipients. *Experimental and Clinical Transplantation: Official*

Journal of the Middle East Society for Organ Transplantation, *16 Suppl 1*(Suppl 1), 117–118. https://doi.org/10.6002/ect.TOND-TDTD2017.P9
70. Chan, W., Bosch, J. A., Jones, D., McTernan, P. G., Phillips, A. C., & Borrows, R. (2014). Obesity in kidney transplantation. *Journal of Renal Nutrition*, *24*(1), 1–12. https://doi.org/10.1053/j.jrn.2013.09.002
71. Chandler, J. L., Sox, L. R., Gunsolley, J. R., Treiber, F. A., & McGillicuddy, J. W. (2017). Associations Between Medication Nonadherence and Perceived Stress Among Kidney Transplant Recipients. *Progress in Transplantation*, *27*(4), 396–397. https://doi.org/10.1177/1526924817732023
72. Chang, Y. H., Lai, Y. H., Tsai, M. K., & Shun, S. C. (2021). Care Needs for Organ Transplant Recipients Scale: Development and psychometric testing. *Journal of Renal Care*, *47*(2), 123–132. https://doi.org/10.1111/jorc.12345
73. Chen, G., Kendall, P. A., Hillers, V. N., & Medeiros, L. C. (2010). Qualitative studies of the food safety knowledge and perceptions of transplant patients. *Journal of Food Protection*, *73*(2), 327–335. https://doi.org/10.4315/0362-028x-73.2.327
74. Chen, K.-H., Weng, L.-C., & Lee, S. (2010). Stress and stress-related factors of patients after renal transplantation in Taiwan: A cross-sectional study. *Journal of Clinical Nursing*, *19*(17–18), 2539–2547. https://doi.org/10.1111/j.1365-2702.2009.03175.x
75. Chen, X., Zhang, Y., & Yu, J. (2021). Symptom Experience and Related Predictors in Liver Transplantation Recipients. *Asian Nursing Research*, *15*(1), 8–14. https://doi.org/10.1016/j.anr.2020.11.001
76. Chen, Y.-W., Wei, J., Chen, H.-L., Cheng, C.-H., & Hou, I.-C. (2020). Developing a Heart Transplantation Self-Management Support Mobile Health App in Taiwan: Qualitative Study. *JMIR mHealth and uHealth*, *8*(8), e18999. https://doi.org/10.2196/18999
77. Chisholm, M. A. (2002). Issues of adherence to immunosuppressant therapy after solid-organ transplantation. *Drugs*, *62*(4), 567–575. https://doi.org/10.2165/00003495-200262040-00002
78. Chisholm, M. A., Fair, J., & Spivey, C. A. (2007). Health literacy and transplant patients and practitioners. *Public Health*, *121*(10), 800–803. https://doi.org/10.1016/j.puhe.2007.03.001
79. Chisholm, M. A., Lance, C. E., Williamson, G. M., & Mulloy, L. L. (2005). Development and validation of an immunosuppressant therapy adherence barrier instrument. *Nephrology Dialysis Transplantation*, *20*(1), 181–188. https://doi.org/10.1093/ndt/gfh576
80. Chisholm, M. A., Mulloy, L. L., & DiPiro, J. T. (2005). Comparing renal transplant patients’ adherence to free cyclosporine and free tacrolimus immunosuppressant therapy. *Clinical Transplantation*, *19*(1), 77–82. https://doi.org/10.1111/j.1399-0012.2004.00301.x
81. Chisholm, M. A., Mulloy, L. L., Jagadeesan, M., & DiPiro, J. T. (2001). Impact of clinical pharmacy services on renal transplant patients’ compliance with immunosuppressive medications. *Clinical Transplantation*, *15*(5), 330–336. https://doi.org/10.1034/j.1399-0012.2001.150505.x
82. Chisholm, M. A., Vollenweider, L. J., Mulloy, L. L., Jagadeesan, M., Wynn, J. J., Rogers, H. E., Wade, W. E., & DiPiro, J. T. (2000). Renal transplant patient compliance with free immunosuppressive medications. *Transplantation*, *70*(8), 1240–1244. https://doi.org/10.1097/00007890-200010270-00020
83. Chisholm, M. A., Williamson, G. M., Lance, C. E., & Mulloy, L. L. (2007). Predicting adherence to immunosuppressant therapy: A prospective analysis of the theory of planned behaviour. *Nephrology Dialysis Transplantation*, *22*(8), 2339–2348. https://doi.org/10.1093/ndt/gfm149
84. Chisholm-Burns, M. A., Spivey, C. A., Graff Zivin, J., Lee, J. K., Sredzinski, E., & Tolley, E. A. (2013). Improving Outcomes of Renal Transplant Recipients With

Behavioral Adherence Contracts: A Randomized Controlled Trial. *American Journal of Transplantation*, *13*(9), 2364–2373. https://doi.org/10.1111/ajt.12341

- University of Tennessee. (2013). *Behavioral Contract Intervention to Improve Adherence Among Renal Transplant Recipients* (Clinical trial registration NCT01739803). clinicaltrials.gov. https://clinicaltrials.gov/ct2/show/NCT01739803

85. Chisholm-Burns, M. A., Spivey, C. A., & Pickett, L. R. (2018). Health literacy in solid-organ transplantation: A model to improve understanding. *Patient Preference and Adherence*, *12*, 2325–2338. https://doi.org/10.2147/PPA.S183092
86. Chisholm-Burns, M. A., Spivey, C. A., & Wilks, S. E. (2010). Social support and immunosuppressant therapy adherence among adult renal transplant recipients. *Clinical Transplantation*, *24*(3), 312–320. https://doi.org/10.1111/j.1399-0012.2009.01060.x
87. Cifani, L., & Vargo, R. (1990). Teaching strategies for the transplant recipient: A review and future directions. *Focus on Critical Care*, *17*(6), 476–479.
88. Ciminelli, A. M., Dupuis, R., Williams, D., Hollar, K., Fair, J. H., Johnson, M., & Gerber, D. (2000). Patient education role of a pharmacist on a transplant service [1]. *American Journal of Health-System Pharmacy*, *57*(8), 767–768. https://doi.org/10.1093/ajhp/57.8.767
89. Clowers-Webb, H. E., Christenson, L. J., Phillips, P. K., Roenigk, R. K., Nguyen, T. H., Weaver, A. L., & Otley, C. C. (2006). Educational outcomes regarding skin cancer in organ transplant recipients: Randomized intervention of intensive vs standard education. *Archives of Dermatology*, *142*(6), 712–718. https://doi.org/10.1001/archderm.142.6.712
90. Coelho Gonçalves, P. R., Ferreira Reveles, A., Fernandes Martins, H. I., Rodrigues, I. L., & Miranda Rodrigues, S. M. (2016). Adherence to Immunosuppressive Therapy in Kidney Transplant Recipients: Integrative Literature Review. *Revista de Enfermagem Referência*, *4*(8), 121–130. https://doi.org/10.12707/RIV14063
91. Coelho Gonçalves, P. R., Loureiro, L. M., & Domingues Fernandes, M. I. (2019). Sexual function of kidney transplant recipients. *Revista de Enfermagem Referência*, *4*(21), 47–56. https://doi.org/10.12707/RIV19009
92. Coerts, F. B., Gout-Zwart, J. J., Gruppen, E. G., van der Veen, Y., Postma, M. J., & Bakker, S. J. L. (2021). Modelling the Cost-Effectiveness of Implementing a Dietary Intervention in Renal Transplant Recipients. *Nutrients*, *13*(4). https://doi.org/10.3390/nu13041175
93. Constantiner, M., & Cukor, D. (2011). Barriers to Immunosuppressive Medication Adherence in High-Risk Adult Renal Transplant Recipients. *Dialysis & Transplantation*, *40*(2), 60–66. https://doi.org/10.1002/dat.20536
94. Constantiner, M., Rosenthal-Asher, D., Tedla, F., Salifu, M., Cukor, J., Wyka, K., Hartono, C., Serur, D., de Boccardo, G., & Cukor, D. (2018). Differences in Attitudes Toward Immunosuppressant Therapy in a Multi-ethnic Sample of Kidney Transplant Recipients. *Journal of Clinical Psychology in Medical Settings*, *25*(1), 11–19. https://doi.org/10.1007/s10880-017-9524-9
95. Conway, A., Schadewaldt, V., Clark, R., Ski, C., Thompson, D. R., & Doering, L. (2013). The psychological experiences of adult heart transplant recipients: A systematic review and meta-summary of qualitative findings. *Heart & Lung*, *42*(6), 449–455. https://dx.doi.org/10.1016/j.hrtlng.2013.08.003
96. Conway, P., Davis, C., Hartel, T., & Russell, G. (1998). Simultaneous kidney pancreas transplantation: Patient issues and nursing interventions. *ANNA Journal*, *25*(5), 455–478.
97. Cormier, N. R., Gallo-Cruz, S. R., & Beard, R. L. (2017). Navigating the new, transplanted self: How recipients manage the cognitive risks of organ transplantation. *Sociology of Health & Illness*, *39*(8), 1496–1513. https://dx.doi.org/10.1111/1467-9566.12610
98. Costa, B., Moratelli, L., Silva, L. B., Paiva, A. C., Silva, A. N., Carminatti, M., Bastos, M. G., & Sanders-Pinheiro, H. (2014). Body mass index in the first year after kidney

transplantation. *Transplantation Proceedings*, *46*(6), 1750–1752. https://doi.org/10.1016/j.transproceed.2014.05.021
99. Costa-Requena, G., Cantarell, M., Moreso, F. J., Parramon, G., & Seron, D. (2017). Health-related behaviours after 1 year of renal transplantation. *Journal of Health Psychology*, *22*(4), 505–514. https://doi.org/10.1177/1359105315604889
100. Cote, J., Fortin, M. C., Auger, P., Rouleau, G., Dubois, S., Boudreau, N., Vaillant, I., & Gelinas-Lemay, E. (2018). Web-Based Tailored Intervention to Support Optimal Medication Adherence Among Kidney Transplant Recipients: Pilot Parallel-Group Randomized Controlled Trial. *JMIR Formative Research*, *2*(2), e14. https://doi.org/10.2196/formative.9707
101. Côté, J., Fortin, M.-C., Auger, P., Rouleau, G., Dubois, S., Vaillant, I., Gelinas-Lemay, E., & Boudreau, N. (2019). Web-Based Tailored Nursing Intervention to Support Medication Self-management A Qualitative Study of the Experience of Kidney Transplant Recipients. *CIN: Computers, Informatics, Nursing*, *37*(11), 564–572. https://doi.org/10.1097/CIN.0000000000000572
102. Cowen, E. W., & Billingsley, E. M. (1999). Awareness of skin cancer by kidney transplant patients. *Journal of the American Academy of Dermatology*, *40*(5 Pt 1), 697–701. https://doi.org/10.1016/s0190-9622(99)70149-0
103. Crawford, K., Low, J. K., Manias, E., & Williams, A. (2017). Healthcare professionals can assist patients with managing post-kidney transplant expectations. *Research In Social & Administrative Pharmacy*, *13*(6), 1204–1207. https://doi.org/10.1016/j.sapharm.2016.11.013
104. Croft, R. Z. (2017). Experience of directed living donor kidney transplant recipients: A literature review. *Nursing Standard*, *32*(3), 41–49. https://doi.org/10.7748/ns.2017.e10256
105. Cyrino, L. G., Galpern, J., Moore, L., Borgi, L., & Riella, L. V. (2021). A Narrative Review of Dietary Approaches for Kidney Transplant Patients. *Kidney International Reports*, *6*(7), 1764–1774. https://doi.org/10.1016/j.ekir.2021.04.009
106. da Silva Knihs, N., de Padua Lorenconi, B., Erbs Pessoa, J. L., Maria Schuantes Paim, S., Fabio Ramos, S., da Silva Martins, M., Fischer Wachholz, L., Bittencourt, I., Fabiane Sebold, L., Nazareth Amante, L., Sens, S., Lunardi, F., Santos, J. D., & de Aguiar Roza, B. (2020). Health Needs of Patients Undergoing Liver Transplant From the Context of Hospital Discharge. *Transplantation Proceedings*, *52*(5), 1344–1349. https://doi.org/10.1016/j.transproceed.2020.02.022
107. Dabbs, A. D. (2018). *Lung Transplant G0 (LTGO): Improving Self-Management of Exercise After Lung Transplantation* (Clinical trial registration NCT03728257). clinicaltrials.gov. https://clinicaltrials.gov/ct2/show/NCT03728257
108. Dabbs, A. D. V., Hoffman, L. A., Swigart, V., Happ, M. B., Dauber, J. H., McCurry, K. R., & Iacono, A. (2004). Striving for normalcy: Symptoms and the threat of rejection after lung transplantation. *Social Science & Medicine*, *59*(7), 1473–1484. https://doi.org/10.1016/j.socscimed.2004.01.013
109. Dabbs, A., Dew, M. A., Myers, B., Begey, A., Hawkins, R., Ren, D. X., Dunbar-Jacob, J., Oconnell, E., & McCurry, K. R. (2009). Evaluation of a hand-held, computer-based intervention to promote early self-care behaviors after lung transplant. *Clinical Transplantation*, *23*(4), 537–545. https://doi.org/10.1111/j.1399-0012.2009.00992.x
110. Dabbs, A., Kim, Y., Hamdan-Mansour, A., Thibodeau, A., & Mc Curry, K. (2006). Health locus of control after lung transplantation: Implications for managing health. *Journal of Clinical Psychology in Medical Settings*, *13*(4), 381–392. https://doi.org/10.1007/s10880-006-9038-3
111. Dahiya, U. (2018). *A study to assess the effectiveness of nurse led self care management programme for kidney transplant recipients* (Clinical trial registration CTRI/2018/12/016630). Clinical Trials Register - India (CTRI). http://www.who.int/trialsearch/Trial2.aspx?TrialID=CTRI/2018/12/016630
112. Dahiya, U. (2021). Adherence to Self-Care among Kidney Transplant Recipients in a Selected Tertiary Care Hospital. *Nursing Journal of India*, *112*(6), 248–252.

113. Dahiya, U., Prakesh, K., Mahajan, S., & Kumar, N. (2020). Psychological symptoms, quality of life and adherence to immunosuppressive therapy in kidney transplant recipients: A cross-sectional study. *Journal of Kidney Care*, *5*(6), 264–271. https://doi.org/10.12968/jokc.2020.5.6.264
114. Dahl, K. G., Andersen, M. H., Urstad, K. H., Falk, R. S., Engebretsen, E., & Wahl, A. K. (2020). Identifying Core Variables Associated With Health Literacy in Kidney Transplant Recipients. *Progress in Transplantation*, *30*(1), 38–47. https://doi.org/10.1177/1526924819893285
115. Dahl, K. G., & Moen, A. (2017). Daily life after a kidney-pancreas transplantation. *Norwegian Journal of Clinical Nursing / Sykepleien Forskning*, *62656*, 1–19. https://doi.org/10.4220/Sykepleienf.2017.62656
116. Dahl, K. G., Wahl, A. K., Urstad, K. H., Falk, R. S., & Andersen, M. H. (2021). Changes in Health Literacy during the first year following a kidney transplantation: Using the Health Literacy Questionnaire. *Patient Education and Counseling*, *104*(7), 1814–1822. https://doi.org/10.1016/j.pec.2020.12.028
117. Dal Sasso-Mendes, K., Ziviani, L. D., Rossin, F. M., Ribeiro, K. P., Pace, A. E., Ohler, L., de Castro-e-Silva, O., & Galvao, C. M. (2011). Perceived Self-Efficacy Among Liver Transplant Candidates and Recipients. *Gastroenterology Nursing*, *34*(3), 236–241. https://doi.org/10.1097/SGA.0b013e31821b47c3
118. Dalvindt, M., Kisch, A., Nozohoor, S., Lennerling, A., & Forsberg, A. (2020). Chronic pain 1-5 years after heart transplantation-A nationwide cross-sectional cohort study. *Nursing Open*, *7*(4), 1146–1156. https://doi.org/10.1002/nop2.489
119. Dalvindt, M., Nozohoor, S., Kisch, A., Lennerling, A., & Forsberg, A. (2020). Symptom Occurrence and Distress after Heart Transplantation-A Nationwide Cross-Sectional Cohort Study. *International Journal of Environmental Research and Public Health*, *17*(21). https://doi.org/10.3390/ijerph17218052
120. David, V., Feldman, D., Danner-Boucher, I., Rhun, A. L., Guyomarch, B., Ravilly, S., & Marchand, C. (2015). Identifying the educational needs of lung transplant recipients with cystic fibrosis. *Progress in Transplantation*, *25*(1), 18–25. https://doi.org/10.7182/pit2015526
    - Nantes University Hospital. (2015). Assessing of the Educational Needs of Patients Who Received a Lung Transplantation or a Simultaneous Heart-lung Transplantation for Mucoviscidosis (Clinical trial registration NCT01285895). clinicaltrials.gov. https://clinicaltrials.gov/ct2/show/NCT01285895
121. de Boer, S., Klewitz, F., Bauer-Hohmann, M., Schiffer, L., Tegtbur, U., Pape, L., Schiffer, M., de Zwaan, M., & Nohre, M. (2020). Knowledge About Immunosuppressant Medication and Its Correlates in a German Kidney Transplant Population—Results of a KTx360degree Substudy. *Patient Preference and Adherence*, *14*, 1699–1708. https://doi.org/10.2147/PPA.S269201
122. de Brito, D. C. S., de Paula, A. M., Rossi dos Santos Grincenkov, Lucchetti, G., & Sanders-Pinheiro, H. (2015). Analysis of the changes and difficulties arising from kidney transplantation: A qualitative study. *Revista Latino-Americana de Enfermagem (RLAE)*, *23*(3), 419–426. https://doi.org/10.1590/0104-1169.0106.2571
123. De Geest, S., Borgermans, L., Gemoets, H., Abraham, I., Vlaminck, H., Evers, G., & Vanrenterghem, Y. (1995). Incidence, determinants, and consequences of subclinical noncompliance with immunosuppressive therapy in renal transplant recipients. *Transplantation*, *59*(3), 340–347.
124. De Geest, S., Burkhalter, H., Berben, L., Bogert, L. J., Denhaerynck, K., Glass, T. R., Goetzmann, L., Kirsch, M., Kiss, A., Koller, M. T., Piot-Ziegler, C., Schmidt-Trucksass, A., & Psychosocial Interest Group, S. T. C. S. (2013). The Swiss Transplant Cohort Study's framework for assessing lifelong psychosocial factors in solid-organ transplants. *Progress in Transplantation*, *23*(3), 235–246. https://doi.org/10.7182/pit2013250

125. De Geest, S., Dobbels, F., Fluri, C., Paris, W., & Troosters, T. (2005). Adherence to the Therapeutic Regimen in Heart, Lung, and Heart-Lung Transplant Recipients. *Journal of Cardiovascular Nursing*, *20*(5S), S88.
126. De Geest, S., Moons, P., Dobbels, F., Martin, S., & Vanhaecke, J. (2001). Profiles of patients who experienced a late acute rejection due to nonadherence with immunosuppressive therapy. *Journal of Cardiovascular Nursing*, *16*(1), 1–14. https://doi.org/10.1097/00005082-200110000-00002
127. De Geest, S., Schafer-Keller, P., Denhaerynck, K., Thannberger, N., Kofer, S., Bock, A., Surber, C., & Steiger, J. (2006). Supporting medication adherence in renal transplantation (SMART): A pilot RCT to improve adherence to immunosuppressive regimens. *Clinical Transplantation*, *20*(3), 359–368. https://doi.org/10.1111/j.1399-0012.2006.00493.x
128. de Oliveira, P. C., Deta, F., Paglione, H. B., Mucci, S., Schirmer, J., & Roza, B. A. (2019). Adherence to liver transplantation treatment: An integrative review. *Cogitare Enfermagem*, *24*, e58326. https://doi.org/10.5380/ce.v24i0.58326
129. De Pasquale, C., Pistorio, M. L., Corona, D., Sinagra, N., Giaquinta, A., Zerbo, D., Veroux, P., & Veroux, M. (2014). Role of „self-efficacy" in the process of long-term care in kidney transplant recipients. *Transplantation Proceedings*, *46*(7), 2235–2237. https://doi.org/10.1016/j.transproceed.2014.07.035
130. De Pasquale, C., Veroux, M., Fornaro, M., Sinagra, N., Basile, G., Gozzo, C., Santini, R., Costa, A., & Pistorio, M. L. (2016). Psychological perspective of medication adherence in transplantation. *World Journal of Transplantation*, *6*(4), 736–742. https://doi.org/10.5500/wjt.v6.i4.736
131. De Pasquale, C., Veroux, M., Indelicato, L., Sinagra, N., Giaquinta, A., Fornaro, M., Veroux, P., & Pistorio, M. L. (2014). Psychopathological aspects of kidney transplantation: Efficacy of a multidisciplinary team. *World Journal of Transplantation*, *4*(4), 267–275. https://doi.org/10.5500/wjt.v4.i4.267
132. De Smet, S., & Van Craenenbroeck, A. H. (2021). Exercise training in patients after kidney transplantation. *Clinical Kidney Journal*, *14*(Suppl 2), ii15–ii24. https://doi.org/10.1093/ckj/sfab022
133. de Vasconcelos, A. G., Pessoa, V., Menezes, F. W. P., Florencio, R. S., & Frota, M. X. F. (2015). Repercussions on the daily living of post-heart transplantation patients. *Acta Paulista De Enfermagem*, *28*(6), 573–579. https://doi.org/10.1590/1982-0194201500094
134. Demian, M. N., Shapiro, R. J., & Thornton, W. L. (2016). An observational study of health literacy and medication adherence in adult kidney transplant recipients. *Clinical Kidney Journal*, *9*(6), 858–865. https://doi.org/10.1093/ckj/sfw076
135. Demian, M. N., Thornton, A. E., Shapiro, R. J., & Loken Thornton, W. (2021). Negative affect and self-agency's association with immunosuppressant adherence in organ transplant: A meta-analysis. *Health Psychology*, *40*(2), 89–103. https://doi.org/10.1037/hea0001047
136. Demir, B., & Demir, I. (2021). Effects of Illness Perception on Self-Care Agency and Hopelessness Levels in Liver Transplant Patients: A Descriptive Cross-Sectional Study. *Clinical Nursing Research*, *31*(3), 473–480. https://doi.org/10.1177/10547738211036983
137. Denhaerynck, K., Berben, L., Dobbels, F., Russell, C. L., Crespo-Leiro, M. G., Poncelet, A. J., De Geest, S., & Bright study, team. (2018). Multilevel factors are associated with immunosuppressant nonadherence in heart transplant recipients: The international BRIGHT study. *American Journal of Transplantation*, *18*(6), 1447–1460. https://doi.org/10.1111/ajt.14611
138. Denhaerynck, K., Dobbels, F., Cleemput, I., Desmyttere, A., Schäfer-Keller, P., Schaub, S., & Geest, S. D. (2005). Prevalence, consequences, and determinants of nonadherence in adult renal transplant patients: A literature review. *Transplant International*, *18*(10), 1121–1133. https://doi.org/10.1111/j.1432-2277.2005.00176.x

139. Denhaerynck, K., Steiger, J., Bock, A., Schafer-Keller, P., Kofer, S., Thannberger, N., & De Geest, S. (2007). Prevalence and risk factors of non-adherence with immunosuppressive medication in kidney transplant patients. *American Journal of Transplantation*, *7*(1), 108–116. https://doi.org/10.1111/j.1600-6143.2006.01611.x
140. DeVito Dabbs, A., Song, M. K., Myers, B. A., Li, R., Hawkins, R. P., Pilewski, J. M., Bermudez, C. A., Aubrecht, J., Begey, A., Connolly, M., Alrawashdeh, M., & Dew, M. A. (2016). A Randomized Controlled Trial of a Mobile Health Intervention to Promote Self-Management After Lung Transplantation. *American Journal of Transplantation*, *16*(7), 2172–2180. https://doi.org/10.1111/ajt.13701
    - University of Pittsburgh. (2009). *Phase III Trial of Pocket PATH: A Computerized Intervention to Promote Self-Care* (Clinical trial registration NCT00818025). clinicaltrials.gov. https://clinicaltrials.gov/ct2/show/NCT00818025
    - DeVito Dabbs, A., Song, M.-K., Myers, B., Hawkins, R. P., Aubrecht, J., Begey, A., Connolly, M., Li, R., Pilewski, J. M., Bermudez, C. A., & Dew, M. A. (2013). Clinical trials of health information technology interventions intended for patient use: Unique issues and considerations. *Clinical Trials*, *10*(6), 896–906. https://doi.org/10.1177/1740774513493149
141. DeVito Dabbs, A., Terhorst, L., Song, M. K., Shellmer, D. A., Aubrecht, J., Connolly, M., & Dew, M. A. (2013). Quality of recipient-caregiver relationship and psychological distress are correlates of self-care agency after lung transplantation. *Clinical Transplantation*, *27*(1), 113–120. https://doi.org/10.1111/ctr.12017
142. Dew, M. A., DiMartini, A. F., De Vito Dabbs, A., Myaskovsky, L., Steel, J., Unruh, M., Switzer, G. E., Zomak, R., Kormos, R. L., & Greenhouse, J. B. (2007). Rates and Risk Factors for Nonadherence to the Medical Regimen After Adult Solid Organ Transplantation. *Transplantation*, *83*(7), 858–873. https://doi.org/10.1097/01.tp.0000258599.65257.a6
143. Dew, M. A., Goycoolea, J. M., Harris, R. C., Lee, A., Zomak, R., Dunbar-Jacob, J., Rotondi, A., Griffith, B. P., & Kormos, R. L. (2004). An internet-based intervention to improve psychosocial outcomes in heart transplant recipients and family caregivers: Development and evaluation. *Journal of Heart and Lung Transplantation*, *23*(6), 745–758.
144. Dew, M. A., Posluszny, D. M., DiMartini, A. F., Myaskovsky, L., Steel, J. L., & DeVito Dabbs, A. J. (2018). Posttransplant Medical Adherence: What Have We Learned and Can We Do Better? *Current Transplantation Reports*, *5*(2), 174–188. https://doi.org/10.1007/s40472-018-0195-8
145. Dew, M. A., Roth, L. H., Thompson, M. E., Kormos, R. L., & Griffith, B. P. (1996). Medical compliance and its predictors in the first year after heart transplantation. *Journal of Heart & Lung Transplantation*, *15*(6), 631–645.
146. Dew, M. A., Simmons, R. G., Roth, L. H., Schulberg, H. C., Thompson, M. E., Armitage, J. M., & Griffith, B. P. (1994). Psychosocial predictors of vulnerability to distress in the year following heart transplantation. *Psychological Medicine*, *24*(4), 929–945. https://doi.org/10.1017/S0033291700029020
147. Dhar, V. K., Kim, Y., Graff, J. T., Jung, A. D., Garrett, J., Dick, L. E., Harris, J., & Shah, S. A. (2018). Benefit of social media on patient engagement and satisfaction: Results of a 9-month, qualitative pilot study using Facebook. *Surgery*, *163*(3), 565–570. https://doi.org/10.1016/j.surg.2017.09.056
148. Diao, D. Y., & Lee, T. K. (2014). Sun-protective behaviors in populations at high risk for skin cancer. *Psychology Research and Behavior Management*, *7*, 9–18. https://doi.org/10.2147/PRBM.S40457
149. DiBenedetto-Cerrato, A. (2008). The relationship between the sick role and functional status in kidney transplant recipients. *Progress in Transplantation*, 159 p-159 p.
150. Dols, J. D., Chargualaf, K. A., Gordon, A., Pomerleau, T., Mendoza, A., Schwarzbach, C., & Gonzalez, M. (2020). Relationship of Nurse-Led Education Interventions to Liver Transplant Early Readmission. *Progress in Transplantation*, *30*(2), 88–94. https://doi.org/10.1177/1526924820913511

151. Donovan, J. C. H., Rosen, C. F., & Shaw, J. C. (2004). Evaluation of sun-protective practices of organ transplant recipients. *American Journal of Transplantation*, *4*(11), 1852–1858. https://doi.org/10.1111/j.1600-6143.2004.00584.x
152. Dontje, M. L., de Greef, M. H., Krijnen, W. P., Corpeleijn, E., Kok, T., Bakker, S. J., Stolk, R. P., & van der Schans, C. P. (2014). Longitudinal measurement of physical activity following kidney transplantation. *Clinical Transplantation*, *28*(4), 394–402. https://doi.org/10.1111/ctr.12325
153. Dowsett, D. A. (1996). Psychological needs of adult patients following renal transplantation and implications for care. *EDTNA/ERCA Journal*, *22*(2), 2–7.
154. Doyle, I. C., Maldonado, A. Q., Heldenbrand, S., Tichy, E. M., & Trofe-Clark, J. (2016). Nonadherence to therapy after adult solid organ transplantation: A focus on risks and mitigation strategies. *American Journal of Health-System Pharmacy*, *73*(12), 909–920. https://doi.org/10.2146/ajhp150650
155. Drent, G., De Geest, S., Dobbels, F., Kleibeuker, J. H., & Haagsma, E. B. (2009). Symptom experience, nonadherence and quality of life in adult liver transplant recipients. *Netherlands Journal of Medicine*, *67*(5), 161–168.
156. Du, C., Wu, S., Liu, H., Hu, Y., & Li, J. (2018). Correlation of long-term medication behaviour self-efficacy with social support and medication knowledge of kidney transplant recipients. *International Journal of Nursing Sciences*, *5*(4), 352–356. https://doi.org/10.1016/j.ijnss.2018.09.009
157. Dubovsky, S. L., Metzner, J. L., & Warner, R. B. (1979). Problems with internalization of a transplanted liver. *American Journal of Psychiatry*, *136*(8), 1090–1091. https://doi.org/10.1176/ajp.136.8.1090
158. Duettmann, W., Naik, M. G., Schmidt, D., Pfefferkorn, M., Kurz, M., Graf, V., Kreichgauer, A., Hoegl, S., Haenska, M., Gielsdorf, T., Breitenstein, T., Osmanodja, B., Glander, P., Bakker, J., Mayrdorfer, M., Gethmann, C. J., Bachmann, F., Choi, M., Schrezenmeier, E., … Budde, K. (2021). Digital Home-Monitoring of Patients after Kidney Transplantation: The MACCS Platform. *Journal of Visualized Experiments*, *170*(4), 12. https://doi.org/10.3791/61899
159. Duke University. (2020). *Duke Transplant University: A Digital Patient Education Portal* (Clinical trial registration NCT04219839). clinicaltrials.gov. https://clinicaltrials.gov/ct2/show/NCT04219839
160. Dunn, B. L., Teusink, A. C., Taber, D. J., Hemstreet, B. A., Uber, L. A., & Weimert, N. A. (2010). Management of hypertension in renal transplant patients: A comprehensive review of nonpharmacologic and pharmacologic treatment strategies. *Annals of Pharmacotherapy*, *44*(7–8), 1259–1270. https://dx.doi.org/10.1345/aph.1P004
161. Eichenberger, P. M., Haschke, M., Lampert, M. L., & Hersberger, K. E. (2011). Drug-related problems in diabetes and transplant patients: An observational study with home visits. *International Journal of Clinical Pharmacy*, *33*(5), 815–823. https://doi.org/10.1007/s11096-011-9542-x
162. Ellingson, T., Wipke-Tevis, D., Messina, C., & Livesay, T. (1999). The use of over-the-counter medications by transplant recipients: A guideline. *Journal of Transplant Coordination*, *9*(1), 17–24. https://doi.org/10.7182/prtr.1.9.1.487821t0k2015512
163. Engle, D. (2001). Psychosocial aspects of the organ transplant experience: What has been established and what we need for the future. *Journal of Clinical Psychology*, *57*(4), 521–549. https://doi.org/DOI 10.1002/jclp.1027
164. Entwistle, T. R., Green, A. C., Fildes, J. E., & Miura, K. (2018). Adherence to Mediterranean and low-fat diets among heart and lung transplant recipients: A randomized feasibility study. *Nutrition Journal*, *17*(22), 1–7. https://doi.org/10.1186/s12937-018-0337-y
165. Epstein, F. R., Liu, C.-M., & Stevenson, J. M. (2019). Heart transplant recipients prefer a telemental health cognitive-behavioral therapy intervention delivered by telephone. *Telemedicine and e-Health*, *25*(7), 560–568. https://doi.org/10.1089/tmj.2018.0088

166. Erdem, C. (2021). *The Effect of Motivational Interviewing on Self-Management in Adult Kidney Transplant Recipients Versus Routine Care* (Clinical trial registration NCT04956406). clinicaltrials.gov. https://clinicaltrials.gov/ct2/show/NCT04956406
167. Erim, Y., Beckmann, M., Klein, C., Paul, A., & Beckebaum, S. (2013). [Psychosomatic aspects of organ transplantation]. *Psychotherapie, Psychosomatik, Medizinische Psychologie*, *63*(6), 238–246. https://dx.doi.org/10.1055/s-0033-1343191
168. Ertel, A. E., Kaiser, T. E., Abbott, D. E., & Shah, S. A. (2016). Use of video-based education and tele-health home monitoring after liver transplantation: Results of a novel pilot study. *Surgery*, *160*(4), 869–876. https://doi.org/10.1016/j.surg.2016.06.016
169. Eslami, S., Khoshrounejad, F., Golmakani, R., Taherzadeh, Z., Tohidinezhad, F., Mostafavi, S. M., & Ganjali, R. (2021). Effectiveness of IT-based interventions on self-management in adult kidney transplant recipients: A systematic review. *BMC Medical Informatics and Decision Making*, *21*(1), 2. https://doi.org/10.1186/s12911-020-01360-2
170. Ettenger, R., Albrecht, R., Alloway, R., Belen, O., Cavaillé-Coll, M. W., Chisholm-Burns, M. A., Dew, M. A., Fitzsimmons, W. E., Nickerson, P., Thompson, G., & Vaidya, P. (2018). Meeting report: FDA public meeting on patient-focused drug development and medication adherence in solid organ transplant patients. *American Journal of Transplantation*, *18*(3), 564–573. https://doi.org/10.1111/ajt.14635
171. Evald, L., Graarup, J., & Hojskov, I. E. (2020). Diary for self-observation: A self-management tool for recipients of lung transplantation-A pilot study. *Nursing Open*, *7*(6), 1766–1773. https://doi.org/10.1002/nop2.562
172. Evangelista, L. S., Doering, L., & Dracup, K. (2003). Meaning and life purpose: The perspectives of post-transplant women. *Heart & Lung*, *32*(4), 250–257. https://doi.org/10.1016/s0147-9563(03)00042-6
173. Evangelista, L. S., Dracup, K., Doering, L., Moser, D. K., & Kobashigawa, J. (2005). Physical activity patterns in heart transplant women. *Journal of Cardiovascular Nursing*, *20*(5), 334–339. https://doi.org/10.1097/00005082-200509000-00007
174. Fadaizadeh, L., Najafizadeh, K., Shajareh, E., Shafaghi, S., Hosseini, M., & Heydari, G. (2016). Home spirometry: Assessment of patient compliance and satisfaction and its impact on early diagnosis of pulmonary symptoms in post-lung transplantation patients. *Journal of Telemedicine and Telecare*, *22*(2), 127–131. https://dx.doi.org/10.1177/1357633X15587435
175. Famure, O., Caballero, M. N., Li, A., Rivera, L., Phan, N., Ashwin, M., Pei Xuan, C., Adcock, L., Schiff, J., & Kim, S. J. (2019). Perceptions of kidney transplant recipients regarding their post-transplant primary care. *CANNT Journal*, *29*(2), 36–43.
176. Fardeazar, F. E., Solhi, M., Soola, A. H., & Amani, F. (2018). Depressive symptoms and associated factors among kidney transplant recipients. *Electronic Journal of General Medicine*, *15*(6), 2–8. https://doi.org/10.29333/ejgm/93460
177. Farmer, S. A., Grady, K. L., Wang, E., McGee, E. C., Cotts, W. G., & McCarthy, P. M. (2013). Demographic, psychosocial, and behavioral factors associated with survival after heart transplantation. *Annals of Thoracic Surgery*, *95*(3), 876–883. https://doi.org/10.1016/j.athoracsur.2012.11.041
178. Fatigati, A., Alrawashdeh, M., Zaldonis, J., & Dabbs, A. D. (2016). Patterns and Predictors of Sleep Quality Within the First Year After Lung Transplantation. *Progress in Transplantation*, *26*(1), 62–69. https://doi.org/10.1177/1526924816632123
179. Faulk, J. S. (2006). Transplant management: Diabetes. Diabetes control and education in transplant care improves outcomes. *Nephrology News & Issues*, *20*(10), 36–41.
180. Febles, R. M., Mena, N. N., Rodriguez-Rodriguez, A. E., Martin, L. D., Rinne, F. G., Miranda, D. M., Rinne, A. G., Gonzalez, A. A., Tamajon, L. P., Sorensen, C. A., Hernandez, A. R., Dominguez-Rodriguez, A., Baute, M. D. G., Ramirez, A. T., & Porrini, E. (2021). Exercise and Prediabetes after Renal Transplantation (EXPRED): Protocol Description. *Nephron*, *145*(1), 55–62. https://doi.org/10.1159/000511320
181. Firooz, A., Amin-Nejad, R., Bouzari, N., Nafar, M., Firoozan, A., & Mahdavi-Mazdeh, M. (2007). Sun protection in Iranian kidney transplant recipients: Knowledge, attitude and

practice. *Journal of the European Academy of Dermatology & Venereology*, *21*(6), 754–757. https://doi.org/10.1111/j.1468-3083.2006.02059.x
182. Fischer Wachholz, L., Knihs, N. S., Sens, S., Schuantes Paim, S. M., Lima Pestana Magalhães, A., & de Aguiar Roza, B. (2021). Good Practices in Transitional Care: Continuity of care for patients undergoing liver transplantation. *Revista Brasileira de Enfermagem*, *74*(2), e20200746. https://doi.org/10.1590/0034-7167-2020-0746
183. Flattery, M. P., Salyer, J., Maltby, M. C., Joyner, P. L., & Elswick, R. K. (2006). Lifestyle and health status differ over time in long-term heart transplant recipients. *Progress in Transplantation*, *16*(3), 232–238.
184. Fleming, J. N., Taber, D. J., McElligott, J., McGillicuddy, J. W., & Treiber, F. (2017). Mobile Health in Solid Organ Transplant: The Time Is Now. *American Journal of Transplantation*, *17*(9), 2263–2276. https://doi.org/10.1111/ajt.14225
185. Flynn, K., Daiches, A., Malpus, Z., Yonan, N., & Sanchez, M. (2014). „A post-transplant person“: Narratives of heart or lung transplantation and intensive care unit delirium. *Health*, *18*(4), 352–368. https://doi.org/10.1177/1363459313501356
186. Fong, J. V. N., & Moore, L. W. (2018). Nutrition Trends in Kidney Transplant Recipients: The Importance of Dietary Monitoring and Need for Evidence-Based Recommendations. *Frontiers in Medicine*, *5*(Article 302). https://doi.org/10.3389/fmed.2018.00302
187. Forsberg, A., Backman, L., & Moller, A. (2000). Experiencing liver transplantation: A phenomenological approach. *Journal of Advanced Nursing*, *32*(2), 327–334. https://doi.org/10.1046/j.1365-2648.2000.01480.x
188. Forsberg, A., Claeson, M., Dahlman, G. B., & Lennerling, A. (2018). Pain, fatigue and well-being one to five years after lung transplantation—A nationwide cross-sectional study. *Scandinavian Journal of Caring Sciences*, *32*(2), 971–978. https://doi.org/10.1111/scs.12537
189. Forsberg, A., Karlsson, V., Cavallini, J., & Lennerling, A. (2016). The meaning of social adaptation after solid organ transplantation. *Nordic Journal of Nursing Research*, *36*(2), 62–67. https://doi.org/10.1177/0107408315603915
190. Forsberg, A., Lennerling, A., Kisch, A., & Jakobsson, S. (2018). Fatigue 1-5 years after lung transplantation: A multicenter cross-sectional cohort study. *Nordic Journal of Nursing Research*, *39*(2), 86–75. https://doi.org/10.1177/2057158518792711
191. Forsberg, A., Nilsson, M., Jakobsson, S., Lennerling, A., & Kisch, A. (2018). Fear of graft rejection 1-5 years after lung transplantation-A nationwide cohort study. *Nursing Open*, *5*(4), 484–490. https://doi.org/10.1002/nop2.184
192. Frame, S. (2011). Health promotion in long-term transplant patients. *Journal of Renal Nursing*, *3*(4), 162–165. https://doi.org/10.12968/jorn.2011.3.4.162
193. Frame, S., & Cronin, A. (2017). Effectiveness of a review clinic for long-term transplant recipients. *Journal of Kidney Care*, *2*(5), 252–260. https://doi.org/10.12968/jokc.2017.2.5.252
194. Franco, T., Warren, J. J., Menke, K. L., Craft, B. J., Cushing, K. A., Gould, D. A., Heermann, J. A., Rogge, J. A., Schmit-Pokorny, K. A., Williams, L., & Woscyna, G. R. (1996). Developing patient and family education programs for a transplant center. *Patient Education and Counseling*, *27*(1), 113–120. https://doi.org/10.1016/0738-3991(95)00795-4
195. Frank-Bader, M., Beltran, K., & Dojlidko, D. (2011). Improving transplant discharge education using a structured teaching approach. *Progress in Transplantation*, *21*(4), 332–339. https://doi.org/10.7182/prtr.21.4.pp1042023304p710
196. Frazier, P. A., Davis-Ali, S. H., & Dahl, K. E. (1994). Correlates of noncompliance among renal transplant recipients. *Clinical Transplantation*, *8*(6), 550–557.
197. Frey, G. M. (1990). Stressors in renal transplant recipients at six weeks after transplant. *ANNA Journal*, *17*(6), 443–446, 450; discussion 447.
198. Fricchione, G. L. (1989). Psychiatric aspects of renal transplantation. *Australian and New Zealand Journal of Psychiatry*, *23*(3), 407–417. https://doi.org/10.3109/00048678909068299

199. Fridgen, R., & Nelson, S. (1992). Teaching tool for renal transplant recipients using Orem's self-care model. *CANNT Journal*, *2*(3), 18–26.
200. Fuller, L. M., Button, B., Tarrant, B., Battistuzzo, C. R., Braithwaite, M., Snell, G., & Holland, A. E. (2014). Patients' expectations and experiences of rehabilitation following lung transplantation. *Clinical Transplantation*, *28*(2), 252–258. https://doi.org/10.1111/ctr.12306
201. Gabay Gillie, G., & Tarabeih, M. (2020). „A Bridge Over Troubled Water": Nurses' Leadership in Establishing Young Adults' Trust Upon the Transition to Adult Renal-Care—A Dual-Perspective Qualitative Study. *Journal of Pediatric Nursing*, *53*, e41–e48. https://doi.org/10.1016/j.pedn.2020.02.004
202. Ganjali, R., Ghorban Sabbagh, M., Nazemiyan, F., Mamdouhi, F., Badiee Aval, S., Taherzadeh, Z., Heshmati Nabavi, F., Golmakani, R., Tohidinezhad, F., & Eslami, S. (2019). Factors Associated With Adherence To Immunosuppressive Therapy And Barriers In Asian Kidney Transplant Recipients. *Immunotargets & Therapy*, *8*, 53–62. https://doi.org/10.2147/ITT.S212760
203. Ganjali, R., Taherzadeh, Z., Sabbagh, M. G., Nazemiyan, F., Mamdouhi, F., Tabesh, H., Aval, S. B., Golmakani, R., Mostafavi, S. M., & Eslami, S. (2019). Effect of an interactive voice response system on self-management in kidney transplant recipients: Protocol for a randomized controlled trial. *Medicine*, *98*(6), e14291. https://doi.org/10.1097/MD.0000000000014291
    - Ganjali, R. (2018). *Designing and evaluating IVR system to improve self-management in kidney transplant recipients* (Clinical trial registration IRCT20180124038492N1). Iranian Registry of Clinical trials (IRCT). http://www.who.int/trialsearch/Trial2.aspx?TrialID=IRCT20180124038492N1
204. Garcia, M., Bravin, A. M., Garcia, P. D., Contti, M. M., Nga, H. S., Takase, H. M., & de Andrade, L. G. M. (2015). Behavioral measures to reduce non-adherence in renal transplant recipients: A prospective randomized controlled trial. *International Urology and Nephrology*, *47*(11), 1899–1905. https://doi.org/10.1007/s11255-015-1104-z
205. Gelb, S. R., Shapiro, R. J., & Thornton, W. J. (2010). Predicting medication adherence and employment status following kidney transplant: The relative utility of traditional and everyday cognitive approaches. *Neuropsychology*, *24*(4), 514–526. https://dx.doi.org/10.1037/a0018670
206. Gellen, E., Papp, B. G., Janka, E. A., Gall, T., Paragh, G., Emri, G., Nemes, B., & Remenyik, E. (2018). Comparison of pre- and post-transplant sun-safe behavior of kidney transplant recipients: What is needed to improve? *Photodermatology, Photoimmunology & Photomedicine*, *34*(5), 322–329. https://doi.org/10.1111/phpp.12387
207. Gentry, A. C. S., Belza, B., & Simpson, T. (2009). Fitness support group for organ transplant recipients: Self-management, self-efficacy and health status. *Journal of Advanced Nursing*, *65*(11), 2419–2425. https://doi.org/10.1111/j.1365-2648.2009.05107.x
208. Geramita, E. M., DeVito Dabbs, A. J., DiMartini, A. F., Pilewski, J. M., Switzer, G. E., Posluszny, D. M., Myaskovsky, L., & Dew, M. A. (2020). Impact of a Mobile Health Intervention on Long-term Nonadherence After Lung Transplantation: Follow-up After a Randomized Controlled Trial. *Transplantation*, *104*(3), 640–651. https://doi.org/10.1097/tp.0000000000002872
209. Gerity, S. L., Silva, S. G., Reynolds, J. M., Hoffman, B., & Oermann, M. H. (2017). Multimedia Education Reduces Anxiety in Lung Transplant Patients. *Progress in Transplantation*, *28*(1), 83–86. https://doi.org/10.1177/1526924817746910
210. Germani, G., Lazzaro, S., Gnoato, F., Senzolo, M., Borella, V., Rupolo, G., Cillo, U., Rigotti, P., Feltrin, G., Loy, M., Martin, A., Sturniolo, G. C., & Burra, P. (2011). Nonadherent Behaviors After Solid Organ Transplantation. *Transplantation Proceedings*, *43*(1), 318–323. https://doi.org/10.1016/j.transproceed.2010.09.103

211. Ghadami, A., Memarian, R., Mohamadi, E., & Abdoli, S. (2012). Patients' experiences from their received education about the process of kidney transplant: A qualitative study. *Iranian Journal of Nursing and Midwifery Research*, *17*(2), 157–164.
212. Gheith, O. A., El-Saadany, S. A., Abuo Donia, S. A., & Salem, Y. M. (2008). Compliance of kidney transplant patients to the recommended lifestyle behaviours: Single centre experience. *International Journal of Nursing Practice*, *14*(5), 398–407. https://doi.org/10.1111/j.1440-172X.2008.00710.x
    - Gheith, O. A., El-Saadany, S. A., Abuo Donia, S. A., & Salem, Y. M. (2008). Compliance with recommended life style behaviors in kidney transplant recipients: Does it matter in living donor kidney transplant? *Iranian Journal of Kidney Diseases*, *2*(4), 218–226.
213. Ghetti, C. M. (2011). Active music engagement with emotional-approach coping to improve well-being in liver and kidney transplant recipients. *Journal of Music Therapy*, *48*(4), 463-485-undefined. https://doi.org/10.1093/jmt/48.4.463
214. Giacoma, T., Ingersoll, G. L., & Williams, M. (1999). Teaching Video Effect on Renal Transplant Patient Outcomes. *ANNA Journal*, *26*(1), 29–30.
215. Giardini, A., Pierobon, A., Majani, G., Biffa, G., Volpe, B., Sala, A., Gangeri, L., Aurelio, M. T., Ferri, F., Colombo, E. M., Fontana, L., Trabucco, G., Travaglini, C., Zoncheddu, P., Nobili, R., Bellapi, S., Masolo, L. S., & Sacchi, A. M. (2011). Adherence self report assessment in solid-organ pre and post transplant recipients. *Giornale Italiano di Medicina del Lavoro Ed Ergonomia*, *33*(1 Suppl A), A69-76.
216. Gibbons, A., Cinnirella, M., Bayfield, J., Watson, C. J. E., Oniscu, G. C., Draper, H., Tomson, C. R. V., Ravanan, R., Johnson, R. J., Forsythe, J., Dudley, C., Metcalfe, W., Bradley, J. A., & Bradley, C. (2020). Changes in quality of life, health status and other patient-reported outcomes following simultaneous pancreas and kidney transplantation (SPKT): A quantitative and qualitative analysis within a UK-wide programme. *Transplant International*, *33*(10), 1230–1243. https://doi.org/10.1111/tri.13677
217. Gibson, C. A., Gupta, A., Greene, J. L., Lee, J., Mount, R. R., & Sullivan, D. K. (2020). Feasibility and acceptability of a televideo physical activity and nutrition program for recent kidney transplant recipients. *Pilot & Feasibility Studies*, *6*, 126-undefined. https://doi.org/10.1186/s40814-020-00672-4
218. Gire Dahl, K., Engebretsen, E., Andersen, M. H., Urstad, K. H., & Wahl, A. K. (2019). The trigger-information-response model: Exploring health literacy during the first six months following a kidney transplantation. *PLoS One*, *14*(10), e0223533-undefined. https://dx.doi.org/10.1371/journal.pone.0223533
219. Godfrey, C., Maylin, S., & Ross-White, A. (2012). Transforming self- the experience of living with another's heart: A systematic review of qualitative evidence on adult heart transplantation. *JBI Library of Systematic Reviews*, *10*(56). https://doi.org/10.11124/jbisrir-2012-271
220. Goetzmann, L., Moser, K. S., Vetsch, E., Klaghofer, R., Naef, R., Russi, E. W., Buddeberg, C., & Boehler, A. (2007). How does psychological processing relate to compliance behaviour after lung transplantation? A content analytical study. *Psychology, Health & Medicine*, *12*(1), 94–106. https://doi.org/10.1080/13548500600780465
221. Goetzmann, L., Scholz, U., Dux, R., Roellin, M., Boehler, A., Muellhaupt, B., Noll, G., Wuthrich, R. P., & Klaghofer, R. (2012). Attitudes towards transplantation and medication among 121 heart, lung, liver and kidney recipients and their spouses. *Swiss Medical Weekly*, *142*, w13595. https://doi.org/10.4414/smw.2012.13595
222. Goetzmann, L., Wagner-Huber, R., Andenmatten-Barenfaller, M., Gunthard, A., Alfare, C., Buddeberg, C., & Boehler, A. (2006). [Group therapy following lung transplantation—First experiences with a theme-centered „life management group“]. *Psychotherapie, Psychosomatik, Medizinische Psychologie*, *56*(7), 272–275. https://doi.org/10.1055/s-2005-915448
223. Gokoel, S. R. M., Gombert-Handoko, K. B., Zwart, T. C., van der Boog, P. J. M., Moes, D., & de Fijter, J. W. (2020). Medication non-adherence after kidney transplantation: A

critical appraisal and systematic review. *Transplantation Reviews*, *34*(1), 100511-undefined. https://doi.org/10.1016/j.trre.2019.100511

224. Goldade, K., Sidhwani, S., Patel, S., Brendt, L., Vigliaturo, J., Kasiske, B., Ahluwalia, J. S., & Israni, A. K. (2011). Kidney transplant patients' perceptions, beliefs, and barriers related to regular nephrology outpatient visits. *American Journal of Kidney Diseases*, *57*(1), 11–20. https://doi.org/10.1053/j.ajkd.2010.08.023
225. Goldstein, N. L., Snyder, M., Edin, C., Lindgren, B., & Finkelstein, S. M. (1996). Comparison of two teaching strategies: Adherence to a home monitoring program. *Clinical Nursing Research*, *5*(2), 150–166. https://doi.org/10.1177/105477389600500204
226. Golfieri, L., Gitto, S., Vukotic, R., Andreone, P., Marra, F., Morelli, M. C., Cescon, M., & Grandi, S. (2019). Impact of psychosocial status on liver transplant process. *Annals of Hepatology*, *18*(6), 804–809. https://dx.doi.org/10.1016/j.aohep.2019.06.011
227. Gomis-Pastor, M., Mirabet Perez, S., Roig Minguell, E., Brossa Loidi, V., Lopez Lopez, L., Ros Abarca, S., Galvez Tugas, E., Mas-Malagarriga, N., & Mangues Bafalluy, M. A. (2021). Mobile Health to Improve Adherence and Patient Experience in Heart Transplantation Recipients: The mHeart Trial. *Healthcare*, *9*(4), Art. 4. https://doi.org/10.3390/healthcare9040463
228. Gonzales, H. M., Fleming, J. N., Gebregziabher, M., Posadas-Salas, M. A., Su, Z., McGillicuddy, J. W., & Taber, D. J. (2021). Pharmacist-Led Mobile Health Intervention and Transplant Medication Safety: A Randomized Controlled Clinical Trial. *Clinical Journal of The American Society of Nephrology: CJASN*, *16*(5), 776–784. https://doi.org/10.2215/CJN.15911020
    - Fleming, J. N., Treiber, F., McGillicuddy, J., Gebregziabher, M., & Taber, D. J. (2018). Improving Transplant Medication Safety Through a Pharmacist-Empowered, Patient-Centered, mHealth-Based Intervention: TRANSAFE Rx Study Protocol. *JMIR Research Protocols*, *7*(3), e59-undefined. https://doi.org/10.2196/resprot.9078
    - Medical University of South Carolina. (2017). *Improving Transplant Medication Safety Through A Pharmacist-Empowered, Patient-Centered, Mhealth-Based Intervention (TRANSAFE Rx Study)* (Clinical trial registration NCT03247322). clinicaltrials.gov. https://clinicaltrials.gov/ct2/show/NCT03247322
229. Gonzalez, A. L., Perez, R. G., Soto, J. B., & Castillo, R. F. (2020). Study of weight and body mass index on graft loss after transplant over 5 years of evolution. *International Journal of Medical Sciences*, *17*(15), 2306–2311. https://doi.org/10.7150/ijms.47000
230. Gordon, E. J., Gallant, M., Sehgal, A. R., Conti, D., & Siminoff, L. A. (2009). Medication-taking among adult renal transplant recipients: Barriers and strategies. *Transplant International*, *22*(5), 534–545. https://doi.org/10.1111/j.1432-2277.2008.00827.x
231. Gordon, E. J., Prohaska, T. R., Gallant, M. P., Sehgal, A. R., Strogatz, D., Conti, D., & Siminoff, L. A. (2010). Prevalence and determinants of physical activity and fluid intake in kidney transplant recipients. *Clinical Transplantation*, *24*(3), E69-81. https://dx.doi.org/10.1111/j.1399-0012.2009.01154.x
232. Gordon, E. J., Prohaska, T. R., Gallant, M. P., Sehgal, A. R., Strogatz, D., Yucel, R., Conti, D., & Siminoff, L. A. (2009). Longitudinal analysis of physical activity, fluid intake, and graft function among kidney transplant recipients. *Transplant International*, *22*(10), 990–998. https://doi.org/10.1111/j.1432-2277.2009.00917.x
233. Gordon, E. J., Prohaska, T. R., Gallant, M., & Siminoff, L. A. (2009). Self-care strategies and barriers among kidney transplant recipients: A qualitative study. *Chronic Illness*, *5*(2), 75–91. https://doi.org/10.1177/1742395309103558
234. Gordon, E. J., Prohaska, T., Siminoff, L. A., Minich, P. J., & Sehgal, A. R. (2005a). Can focusing on self-care reduce disparities in kidney transplantation outcomes? *American Journal of Kidney Diseases*, *45*(5), 935–940. https://doi.org/10.1053/j.ajkd.2005.02.011

235. Gordon, E. J., Prohaska, T., Siminoff, L. A., Minich, P. J., & Sehgal, A. R. (2005b). Needed: Tailored exercise regimens for kidney transplant recipients. *American Journal of Kidney Diseases*, *45*(4), 769–774. https://doi.org/10.1053/j.ajkd.2005.01.002
236. Gordon, E. J., & Wolf, M. S. (2009). Health literacy skills of kidney transplant recipients. *Progress in Transplantation*, *19*(1), 25–34. https://doi.org/10.1177/152692480901900104
237. Graarup, J., Mogensen, E. L., Missel, M., & Berg, S. K. (2017). Life after a lung transplant: A balance of joy and challenges. *Journal of Clinical Nursing*, *26*(21–22), 3543–3552. https://doi.org/10.1111/jocn.13724
238. Grady, K. L., & Jalowiec, A. (1995). Predictors of compliance with diet 6 months after heart transplantation. *Heart & Lung*, *24*(5), 359–368. https://doi.org/10.1016/s0147-9563(05)80056-1
239. Grady, K. L., Jalowiec, A., & White-Williams, C. (1998). Patient compliance at one year and two years after heart transplantation. *Journal of Heart & Lung Transplantation*, *17*(4), 383–394.
240. Grady, K. L., Wang, E., White-Williams, C., Naftel, D. C., Myers, S., Kirklin, J. K., Rybarczyk, B., Young, J. B., Pelegrin, D., Kobashigawa, J., Higgins, R., & Heroux, A. (2013). Factors associated with stress and coping at 5 and 10 years after heart transplantation. *Journal of Heart & Lung Transplantation*, *32*(4), 437–446. https://dx.doi.org/10.1016/j.healun.2012.12.012
241. Greene, G. M., Merighi, J. R., Voorhes, P., & McCool, M. (2020). A Multisite Study on Using Symptom-Targeted Interventions to Improve Mental Health Outcomes of Solid Organ Transplant Patients. *Progress in Transplantation*, *30*(2), 132–139. https://doi.org/10.1177/1526924820913513
242. Greenstein, S. M., & Siegal, B. (1997). Postrenal transplant health beliefs and ethnicity. The Compliance Study Group. *Transplantation Proceedings*, *29*(8), 3741–3742. https://doi.org/10.1016/s0041-1345(97)01093-2
243. Gremigni, P., Bacchi, F., Turrini, C., Cappelli, G., Albertazzi, A., & Bitti, P. E. (2007). Psychological factors associated with medication adherence following renal transplantation. *Clinical Transplantation*, *21*(6), 710–715. https://doi.org/10.1111/j.1399-0012.2007.00727.x
244. Grijpma, J. W., Tielen, M., van Staa, A. L., Maasdam, L., van Gelder, T., Berger, S. P., Busschbach, J. J., Betjes, M. G. H., Weimar, W., & Massey, E. K. (2016). Kidney transplant patients' attitudes towards self-management support: A Q-methodological study. *Patient Education and Counseling*, *99*(5), 836–843. https://doi.org/10.1016/j.pec.2015.11.018
245. Gross, C. R., Kreitzer, M. J., Russas, V., Treesak, C., Frazier, P. A., & Hertz, M. I. (2004). Mindfulness meditation to reduce symptoms after organ transplant: A pilot study. *Alternative Therapies in Health & Medicine*, *10*(3), 58–66.
    - Kreitzer, M. J., Gross, C. R., Ye, X., Russas, V., & Treesak, C. (2005). Longitudinal Impact of Mindfulness Meditation on Illness Burden in Solid-Organ Transplant Recipients. *Progress in Transplantation*, *15*(2), 166–172. https://doi.org/10.1177/152692480501500210
246. Gross, C. R., Kreitzer, M. J., Thomas, W., Reilly-Spong, M., Cramer-Bornemann, M., Nyman, J. A., Frazier, P., & Ibrahim, H. N. (2010). Mindfulness-based stress reduction for solid organ transplant recipients: A randomized controlled trial. *Alternative Therapies in Health & Medicine*, *16*(5), 30–38.
    - Gross, C. R., Kreitzer, J. M., Reilly-Spong, M., Winbush, N. Y., Schomaker, K. E., & Thomas, W. (2009). Mindfulness meditation training to reduce symptom distress in transplant patients: Rationale, design, and experience with a recycled waitlist. *Clinical Trials*, *6*(1), 76-89. https://doi.org/10.1177/1740774508100982
    - National Institute of Nursing Research (NINR). (2006). *Impact of Mind-Body Interventions Post Organ Transplant* (Clinical trial registration NCT00367809). clinicaltrials.gov. https://clinicaltrials.gov/ct2/show/NCT00367809

247. Grumme, V. S., & Gordon, S. C. (2016). Social Media Use by Transplant Recipients for Support and Healing. *CIN: Computers, Informatics, Nursing*, *34*(12), 570–577. https://doi.org/10.1097/Cin.0000000000000276
248. Guazzelli, C. A. F., Torloni, M. R., Sanches, T. F., Barbieri, M., & Pestana, J. (2008). Contraceptive counseling and use among 197 female kidney transplant recipients. *Transplantation*, *86*(5), 669–672. https://doi.org/10.1097/TP.0b013e3181817e7d
249. Guevara, Y., Gaber, R., Clayman, M. L., Gordon, E. J., Friedewald, J., & Robinson, J. K. (2015). Sun Protection Education for Diverse Audiences: Need for Skin Cancer Pictures. *Journal of Cancer Education*, *30*(1), 187–189. https://doi.org/10.1007/s13187-014-0661-7
250. Guldager, T. B., Hyldgaard, C., Hilberg, O., & Bendstrup, E. (2021). An E-Learning Program Improves Patients' Knowledge After Lung Transplantation. *Telemedicine and e-Health*, *27*(7), 800–806. https://doi.org/10.1089/tmj.2020.0101
251. Gunn, K. M., Ross, X. S., McLoughlin, M., McDonald, S., & Olver, I. (2021). The psychosocial experiences and supportive care preferences of organ transplant recipients and their carers who live in regional Australia. *Australian Journal of Rural Health*, *29*(1), 92–105. https://doi.org/10.1111/ajr.12693
252. Gustaw, T., Schoo, E., Barbalinardo, C., Rodrigues, N., Zameni, Y., Motta, V. N., Mathur, S., & Janaudis-Ferreira, T. (2017). Physical activity in solid organ transplant recipients: Participation, predictors, barriers, and facilitators. *Clinical Transplantation*, *31*(4). https://doi.org/10.1111/ctr.12929
253. Hamama-Raz, Y., Bergman, Y. S., Ben-Ezra, M., Tirosh, Y., Baruch, R., & Nakache, R. (2018). Attachment patterns moderate the relation between coping flexibility and illness acceptance among kidney transplant recipients. *Anxiety, Stress, & Coping*, *31*(5), 571–579. https://doi.org/10.1080/10615806.2018.1498667
254. Hammond, C., Hussaini, T., & Yoshida, E. M. (2021). Medical adherence and liver transplantation: A brief review. *Canadian Liver Journal*, *4*(1), 8–15. https://doi.org/10.3138/canlivj-2020-0016
255. Haney, M. O., Ordin, Y. S., & Arkan, G. (2019). Skin Cancer-Sun Knowledge and Sun Protection Behaviors of Liver Transplant Recipients in Turkey. *Journal of Cancer Education*, *34*(1), 137–144. https://dx.doi.org/10.1007/s13187-017-1279-3
256. Hannover Medical School. (2008). *Empowerment of Lung and Heart-lung Transplant Patients by a Multimodal Resource-activating Behavioural Training Programme and Cardiopulmonary Exercise—A Randomised Controlled Study* (Clinical trial registration NCT00808600). clinicaltrials.gov. https://clinicaltrials.gov/ct2/show/NCT00808600
257. Hannover Medical School. (2009). *Medication Adherence Enhancement in Heart Transplant Recipients: A Randomized Clinical Trial* (Clinical trial registration NCT00843960). clinicaltrials.gov. https://clinicaltrials.gov/ct2/show/NCT00843960
258. Hanssen Textor, L., & Hedrick, J. (2012). The lived experience of peripheral neuropathy after solid organ transplant. *Progress in Transplantation*, *22*(3), 271–279. https://doi.org/10.7182/pit2012703
259. Hap, K., Madziarska, K., Hap, W., & Mazanowska, O. (2014). Phenotypic risk factors for new-onset diabetes mellitus (NODAT) in renal transplant recipients. *Postepy Higieny i Medycyny do Swiadczalnej*, *68*, 1347–1351. https://doi.org/10.5604/17322693.1129186
260. Hardstaff, R., Green, K., & Talbot, D. (2003). Measurement of compliance posttransplantation—The results of a 12-month study using electronic monitoring. *Transplantation Proceedings*, *35*(2), 796–797. https://doi.org/10.1016/S0041-1345(03)00074-5
261. Harris, F. (1996). A new way of life... Renal failure... Transplantation. *Nursing Times*, *92*(8), 52–54.
262. Harrison, J. J., Badr, S., Hamandi, B., & Kim, S. J. (2017). Randomized Controlled Trial of a Computer-Based Education Program in the Home for Solid Organ Transplant Recipients: Impact on Medication Knowledge, Satisfaction, and Adherence. *Transplantation*, *101*(6), 1336–1343. https://doi.org/10.1097/TP.0000000000001279

- Harrison, J. (2013). Impact of an E-learning Tool on Patient Satisfaction, Knowledge and Medication Adherence in Solid Organ Transplantation: A Randomized Controlled Trial (Clinical trial registration NCT01826721). clinicaltrials.gov. https://clinicaltrials.gov/ct2/show/NCT01826721

263. Hart, L. K., Milde, F. K., Zehr, P. S., Cox, D. M., Tarara, D. T., & Fearing, M. O. (1997). Survey of sexual concerns among organ transplant recipients. *Journal of Transplant Coordination*, *7*(2), 82–87. https://doi.org/10.7182/prtr.1.7.2.m53l331744v1932u
264. Hartman, R. I., Green, A. C., & Gordon, L. G. (2018). Sun Protection Among Organ Transplant Recipients After Participation in a Skin Cancer Research Study. *JAMA Dermatology*, *154*(7), 842–844. https://doi.org/10.1001/jamadermatol.2018.1164
265. Haspeslagh, A., De Bondt, K., Kuypers, D., Naesens, M., Breunig, C., & Dobbels, F. (2013). Completeness and Satisfaction with the Education and Information Received by Patients Immediately after Kidney Transplant: A Mixed-Models Study. *Progress in Transplantation*, *23*(1), 12–22. https://doi.org/10.7182/pit2013249
266. Hasse, J. M. (1997). Diet therapy for organ transplantation. A problem-based approach. *Nursing Clinics of North America*, *32*(4), 863–880.
267. Hayward, M. B., Kish, J. P., Frey, G. M., Kirchner, J. M., Carr, L. S., & Wolfe, C. M. (1989). An instrument to identify stressors in renal transplant recipients. *ANNA Journal*, *16*(2), 81–85.
268. Hedayati, P., Shahgholian, N., & Ghadami, A. (2017). Nonadherence Behaviors and Some Related Factors in Kidney Transplant Recipients. *Iranian Journal of Nursing and Midwifery Research*, *22*(2), 97–101. https://doi.org/10.4103/ijnmr.IJNMR_220_15
269. Helmy, R., Duerinckx, N., De Geest, S., Denhaerynck, K., Berben, L., Russell, C. L., Van Cleemput, J., Crespo-Leiro, M. G., Dobbels, F., & Bright Study, T. (2018). The international prevalence and variability of nonadherence to the nonpharmacologic treatment regimen after heart transplantation: Findings from the cross-sectional BRIGHT study. *Clinical Transplantation*, *32*(7). https://doi.org/10.1111/ctr.13280
270. Hermann, H. C., Grimm, A., Klapp, B. F., Neuhaus, R., & Papachristou, C. (2017). Body Experience After Liver Transplantation: A Body Grid Examination. *Journal of Constructivist Psychology*, *30*(3), 295–314. https://doi.org/10.1080/10720537.2016.1227736
271. Heyes, C. M., Schofield, T., Gribble, R., Day, C. A., & Haber, P. S. (2016). Reluctance to Accept Alcohol Treatment by Alcoholic Liver Disease Transplant Patients: A Qualitative Study. *Transplantation Direct*, *2*(10), e104. https://doi.org/10.1097/TXD.0000000000000617
272. Hickman, I. J., Coran, D., Wallen, M. P., Kelly, J., Barnett, A., Gallegos, D., Jarrett, M., Mccoy, S. M., Campbell, K. L., & Macdonald, G. A. (2019). 'Back to Life'-Using knowledge exchange processes to enhance lifestyle interventions for liver transplant recipients: A qualitative study. *Nutrition & Dietetics*, *76*(4), 399–406. https://doi.org/10.1111/1747-0080.12548
273. Hickman, I. J., Hannigan, A. K., Johnston, H. E., Elvin-Walsh, L., Mayr, H. L., Staudacher, H. M., Barnett, A., Stoney, R., Salisbury, C., Jarrett, M., Reeves, M. M., Coombes, J. S., Campbell, K. L., Keating, S. E., & Macdonald, G. A. (2021). Telehealth-delivered, Cardioprotective Diet and Exercise Program for Liver Transplant Recipients: A Randomized Feasibility Study. *Transplantation Direct*, *7*(3). https://doi.org/10.1097/TXD.0000000000001118
274. Hlubocky, J. M., Stuckey, L. J., Schuman, A. D., & Stevenson, J. G. (2012). Evaluation of a transplantation specialty pharmacy program. *American Journal of Health-System Pharmacy*, *69*(4), 340–347. https://doi.org/10.2146/ajhp110350
275. Hochheimer, M., Moreland, M. L., Tuten, M., LaMattina, J., Connelly, M., & Sacco, P. (2019). Insights Into the Experience of Liver Transplant Recipients With Alcoholic Liver Disease: A Descriptive Qualitative Study. *Transplantation Direct*, *5*(12), e506-undefined. https://doi.org/10.1097/TXD.0000000000000951
276. Hoffart, N. (1982). Self-care decision making by renal transplant recipients. *AANNT Journal*, *9*(3), 43–47.

277. Holderried, M., Hoeper, A., Holderried, F., Heyne, N., Nadalin, S., Unger, O., Ernst, C., & Guthoff, M. (2021). Attitude and potential benefits of modern information and communication technology use and telemedicine in cross-sectoral solid organ transplant care. *Scientific Reports*, *11*(1), Art. 1. https://doi.org/10.1038/s41598-021-88447-6
278. Hook, M. L., Heyse, T. J., Pawlak, J. C., & Steckelberg, J. M. (1990). Psychosocial care of the cardiac transplant patient: A nursing diagnosis approach. *Dimensions Of Critical Care Nursing*, *9*(5), 301–309. https://doi.org/10.1097/00003465-199009000-00014
279. Howell, M., Tong, A., Wong, G., Craig, J. C., & Howard, K. (2012). Important outcomes for kidney transplant recipients: A nominal group and qualitative study. *American Journal of Kidney Diseases*, *60*(2), 186–196. https://doi.org/10.1053/j.ajkd.2012.02.339
280. Hrenczuk, M., Bieniak, A., Pazik, J., & Malkowski, P. (2018). Analysis of Health Behaviors in Patients After Liver Transplant. *Transplantation Proceedings*, *50*(10), 3587–3593. https://doi.org/10.1016/j.transproceed.2018.08.061
281. Hsiao, C. Y., Lin, L. W., Su, Y. W., Yeh, S. H., Lee, L. N., & Tsai, F. M. (2016). The Effects of an Empowerment Intervention on Renal Transplant Recipients: A Randomized Controlled Trial. *Journal of Nursing Research*, *24*(3), 201–210. https://doi.org/10.1097/jnr.0000000000000115
282. Hu, L., DeVito Dabbs, A., Dew, M. A., Sereika, S. M., & Lingler, J. H. (2017). Patterns and correlates of adherence to self-monitoring in lung transplant recipients during the first 12 months after discharge from transplant. *Clinical Transplantation*, *31*(8), 10.1111/ctr.13014. https://doi.org/10.1111/ctr.13014
283. Hu, L., Lingler, J. H., DeVito Dabbs, A., Dew, M. A., & Sereika, S. M. (2017). Trajectories of self-care agency and associated factors in lung transplant recipients over the first 12 months following transplantation. *Clinical Transplantation*, *31*(9). https://doi.org/10.1111/ctr.13030
284. Hu, L., Lingler, J. H., Sereika, S. M., Burke, L. E., Malchano, D. K., DeVito Dabbs, A., & Dew, M. A. (2017). Nonadherence to the medical regimen after lung transplantation: A systematic review. *Heart & Lung*, *46*(3), 178–186. https://doi.org/10.1016/j.hrtlng.2017.01.006
285. Hu, R., Gu, B., Tan, Q., Xiao, K., Li, X., Cao, X., Song, T., & Jiang, X. (2020). The effects of a transitional care program on discharge readiness, transitional care quality, health services utilization and satisfaction among Chinese kidney transplant recipients: A randomized controlled trial. *International Journal of Nursing Studies*, *110*, 103700. https://doi.org/10.1016/j.ijnurstu.2020.103700
286. Huang, S., Wang, S., Tai, J. J., & Lou, M. (2008). Unplanned readmission within the most recent postoperative year of heart transplant patients in Taiwan. *Journal of Clinical Nursing*, *17*(17), 2302–2310. https://doi.org/10.1111/j.1365-2702.2007.02268.x
287. Hugon, A., Roustit, M., Lehmann, A., Saint-Raymond, C., Borrel, E., Hilleret, M. N., Malvezzi, P., Bedouch, P., Pansu, P., & Allenet, B. (2014). Influence of intention to adhere, beliefs and satisfaction about medicines on adherence in solid organ transplant recipients. *Transplantation*, *98*(2), 222–228. https://doi.org/10.1097/TP.0000000000000221
288. Huuskes, B. M., Scholes-Robertson, N., Guha, C., Baumgart, A., Wong, G., Kanellis, J., Chadban, S., Barraclough, K. A., Viecelli, A. K., Hawley, C. M., Kerr, P. G., Toby Coates, P., Amir, N., & Tong, A. (2021). Kidney transplant recipient perspectives on telehealth during the COVID-19 pandemic. *Transplant International*, *34*(8), 1517–1529. https://doi.org/10.1111/tri.13934
289. Hwang, H. (1996). Patient and family adjustment to heart transplantation. *Progress in Cardiovascular Nursing*, *11*(2), 16–39.
290. Hwang, Y., Kim, M., & Min, K. (2021). Factors associated with health-related quality of life in kidney transplant recipients in Korea. *PLoS One*, *16*(3), e0247934. https://doi.org/10.1371/journal.pone.0247934

291. Iannacone, M. R., Pandeya, N., Isbel, N., Campbell, S., Fawcett, J., Soyer, H. P., Ferguson, L., Davis, M., Whiteman, D. C., & Green, adèle C. (2015). Sun Protection Behavior in Organ Transplant Recipients in Queensland, Australia. *Dermatology*, *231*(4), 360–366. https://doi.org/10.1159/000439428
292. Ichimaru, N., Kawamura, M., Nakazawa, S., Kato, T., Abe, T., Kaimori, J. Y., Imamura, R., Moriyama, T., & Nonomura, N. (2019). Adherence to Dietary Recommendations Measured by Smartphone-based Recipe Nutrition Calculator in Kidney Transplant Patients. *Transplantation Proceedings*, *51*(5), 1362–1364. https://doi.org/10.1016/j.transproceed.2019.03.021
293. Inspector, Y., Kutz, I., & David, D. (2004). Another person's heart: Magical and rational thinking in the psychological adaptation to heart transplantation. *Israel Journal of Psychiatry & Related Sciences*, *41*(3), 161–173.
294. Ivarsson, B., Ekmehag, B., & Sjöberg, T. (2013). Patients' experiences of information and support during the first six months after heart or lung transplantation. *European Journal of Cardiovascular Nursing*, *12*(4), 400–406. https://doi.org/10.1177/1474515112466155
295. Jagielska, A., Jankowski, K., OkrĘGlicka, K., Skupin, P., KozŁOwska, A., Durlik, M., Pruszczyk, P., Nitsch-Osuch, A., & Tronina, O. (2017). Lifestyle risk factors of non-communicable diseases among patients after liver transplantation—A pilot study. *Family Medicine & Primary Care Review*, *19*(3), 214–220. https://doi.org/10.5114/fmpcr.2017.69277
296. Jain, A., Humar, A., Lien, D., Weinkauf, J., & Kumar, D. (2015). Strategies for safe living among lung transplant recipients: A single-center survey. *Transplant Infectious Disease: An Official Journal of the Transplantation Society*, *17*(2), 185–191. https://doi.org/10.1111/tid.12354
297. Jamieson, N. J., Hanson, C. S., Josephson, M. A., Gordon, E. J., Craig, J. C., Halleck, F., Budde, K., & Tong, A. (2016). Motivations, Challenges, and Attitudes to Self-management in Kidney Transplant Recipients: A Systematic Review of Qualitative Studies. *American Journal of Kidney Diseases*, *67*(3), 461–478. https://doi.org/10.1053/j.ajkd.2015.07.030
298. Jeng, C., Chu, F. L., & Tsao, L. I. (2002). Empowering: The experiences of exercise among heart transplantation patients in Taiwan. *Journal of Advanced Nursing*, *40*(5), 560–567. https://doi.org/10.1046/j.1365-2648.2002.02414.x
299. Jeong, H. W., Song, C. E., & An, M. (2021). Feasibility and preliminary effects of a theory-based self-management program for kidney transplant recipients: A pilot study. *PLoS One*, *16*(6), e0248947. https://doi.org/10.1371/journal.pone.0248947
300. Jesse, M. T., Rubinstein, E., Eshelman, A., Wee, C., Tankasala, M., Li, J., & Abouljoud, M. (2016). Lifestyle and Self-Management by Those Who Live It: Patients Engaging Patients in a Chronic Disease Model. *The Permanente Journal*, *20*(3), 45–50. https://doi.org/10.7812/TPP/15-207
301. Jezior, D., Krajewska, M., Madziarska, K., Regulska-Ilow, B., Ilow, R., Janczak, D., Patrzalek, D., & Klinger, M. (2007). Weight reduction in renal transplant recipients program: The first successes. *Transplantation Proceedings*, *39*(9), 2769–2771. https://doi.org/10.1016/j.transproceed.2007.08.055
302. Jiang, Y., Sereika, S. M., Dabbs, A. D., Handler, S. M., & Schlenk, E. A. (2016a). Acceptance and Use of Mobile Technology for Health Self-Monitoring in Lung Transplant Recipients during the First Year Post-Transplantation. *Applied Clinical Informatics*, *7*(2), 430–445. https://doi.org/10.4338/ACI-2015-12-RA-0170
303. Jiang, Y., Sereika, S. M., Dabbs, A. D., Handler, S. M., & Schlenk, E. A. (2016b). Using mobile health technology to deliver decision support for self-monitoring after lung transplantation. *International Journal of Medical Informatics*, *94*, 164–171. https://doi.org/10.1016/j.ijmedinf.2016.07.012
304. Jiayun, X., Adeboyejo, O., Wagley, E., Aubrecht, J., Mi-Kyung, S., Thiry, L., & DeVito Dabbs, A. (2012). Daily burdens of recipients and family caregivers after lung

transplant. *Progress in Transplantation*, *22*(1), 41–48. https://doi.org/10.7182/pit2012815
305. Jindal, R. M., Joseph, J. T., Morris, M. C., Santella, R. N., & Baines, L. S. (2003). Noncompliance after kidney transplantation: A systematic review. *Transplantation Proceedings*, *35*(8), 2868–2872. https://doi.org/10.1016/j.transproceed.2003.10.052
306. Johann, B., & Erim, Y. (2001). [Psychosomatic medicine and transplant patients—What is known and what is needed]. *Psychotherapie, Psychosomatik, Medizinische Psychologie*, *51*(12), 438–446. https://doi.org/10.1055/s-2001-19403
307. John, L. (2006). Transplant diary. *Australian Nursing Journal*, *13*(9), 38–39.
308. Johnson, L. C., & Goldstein, N. L. (1992). A comparison of two patient teaching methods: One-on-one versus group instruction. *Journal of Transplant Coordination*, *3*(SUPPL. 1), 9-14.
309. Jones, J., Damery, S., Allen, K., Nicholas, J., Baharani, J., & Combes, G. (2020). „You have got a foreign body in there“: Renal transplantation, unexpected mild-to-moderate distress and patients' support needs: A qualitative study. *BMJ Open*, *10*(3), e035627-undefined. https://dx.doi.org/10.1136/bmjopen-2019-035627
310. Jones, L. S., & Serper, M. (2020). Medication non-adherence among liver transplant recipients. *Current Hepatology Reports*, *19*(4), 327–336. https://doi.org/10.1007/s11901-020-00545-7
311. Jonsen, E., Athlin, E., & Suhr, O. (1998). Familial amyloidotic patients' experience of the disease and of liver transplantation. *Journal of Advanced Nursing*, *27*(1), 52–58. https://doi.org/10.1046/j.1365-2648.1998.00503.x
312. Jordakieva, G., Grabovac, I., Steiner, M., Winnicki, W., Zitta, S., Stefanac, S., Brooks, M., Sunder-Plassmann, G., Rosenkranz, A. R., & Godnic-Cvar, J. (2020). Employment Status and Associations with Workability, Quality of Life and Mental Health after Kidney Transplantation in Austria. *International Journal of Environmental Research and Public Health*, *17*(4). https://doi.org/10.3390/ijerph17041254
313. Ju, A., Josephson, M. A., Butt, Z., Jowsey-Gregoire, S., Tan, J., Taylor, Q., Fowler, K., Dobbels, F., Caskey, F., Jha, V., Locke, J., Knoll, G., Ahn, C., Hanson, C. S., Sautenet, B., Manera, K., Craig, J. C., Howell, M., Rutherford, C., … S. Ong- Tx Life Participation Works. (2019). Establishing a Core Outcome Measure for Life Participation: A Standardized Outcomes in Nephrology-kidney Transplantation Consensus Workshop Report. *Transplantation*, *103*(6), 1199–1205. https://doi.org/10.1097/tp.0000000000002476
314. Ju, M. K., Son, S., & Kim, S. (2016). Adjustment Experience of Kidney Transplantation Recipients in Korea. *Transplantation Proceedings*, *48*(7), 2434–2436. https://doi.org/10.1016/j.transproceed.2016.02.098
315. Jung, H. Y., Jeon, Y., Seong, S. J., Seo, J. J., Choi, J. Y., Cho, J. H., Park, S. H., Kim, C. D., Yoon, Y. R., Yoon, S. H., Lee, J. S., & Kim, Y. L. (2020). ICT-based adherence monitoring in kidney transplant recipients: A randomized controlled trial. *BMC Medical Informatics and Decision Making*, *20*(1), 105. http://dx.doi.org/10.1186/s12911-020-01146-6
316. Kaba, E., & Shanley, E. (1997). Identification of coping strategies used by heart transplant recipients. *British Journal of Nursing*, *6*(15), 858–862. https://doi.org/10.12968/bjon.1997.6.15.858
317. Kaba, E., Thompson, D., Burnard, P., Edwards, D., & Theodosopoulou, E. (2005). Somebody else's heart inside me: A descriptive study of psychological problems after a heart transplantation. *Issues in Mental Health Nursing*, *26*(6), 611–625. https://doi.org/10.1080/01612840590959452
318. Kaba, E., Thompson, D. R., & Burnard, P. (2000). Coping after heart transplantation: A descriptive study of heart transplant recipients' methods of coping. *Journal of Advanced Nursing*, *32*(4), 930–936. https://doi.org/10.1046/j.1365-2648.2000.t01-1-01558.x
319. Kadowaki, M., Saito, M., Amada, N., Haga, I., Nakamura, A., & Tokodai, K. (2014). Medication compliance in renal transplant patients during the Great East Japan

Earthquake. *Transplantation Proceedings*, *46*(2), 610–612. https://dx.doi.org/10.1016/j.transproceed.2013.11.039

320. Kamran, F., & Ghazal, S. (2017). The Journey From Illness to Wellness: An Exploration of Sociocultural Issues After Renal Transplantation in Pakistan. *Journal of Behavioural Sciences*, *27*(1), 127–147.
321. Karepalli, V. K., Brown, M., Sajiv, C., Fernandes, D., Thomas, S., George, P., Nayar, S., & Pawar, B. (2021). Barriers and challenges of returning patients back to community after renal transplantation in Central Australia. *Internal Medicine Journal*, *51*(9), 1479–1484. https://doi.org/10.1111/imj.15196
322. Kasbia, G. S., Farragher, J., Kim, S. J., Famure, O., & Jassal, S. V. (2014). A cross-sectional study examining the functional independence of elderly individuals with a functioning kidney transplant. *Transplantation*, *98*(8), 864–870. https://doi.org/10.1097/TP.0000000000000126
323. Kauffels, A., Schmalz, G., Kollmar, O., Slotta, J. E., Weig, M., Groß, U., Bader, O., & Ziebolz, D. (2017). Oral findings and dental behaviour before and after liver transplantation – a single-centre cross-sectional study. *International Dental Journal*, *67*(4), 244–251. https://doi.org/10.1111/idj.12290
324. Kauffman, H. M., Woodle, E. S., Cole, E. H., Paykin, C., & National Kidney, F. (2008). Transplant recipient's knowledge of posttransplant malignancy risk: Implications for educational programs. *Transplantation*, *85*(7), 928–933. https://dx.doi.org/10.1097/TP.0b013e31816a105b
325. Keidar, R., Katz, P., & Nakache, R. (2001). Resuming life after transplantation: Development of a support group for patients and their families. *Transplantation Proceedings*, *33*(1–2), 1911–1912. https://doi.org/10.1016/S0041-1345(00)02711-1
    - Kiedar, R., Katz, P., & Nakache, R. (2001). „Living again": Heterogeneous support group for transplant patients and their families. *Transplantation Proceedings*, *33*(6), 2930–2931. https://doi.org/10.1016/S0041-1345(01)02255-2
326. Kenawy, A. S., Gheith, O., Al-Otaibi, T., Othman, N., Abo Atya, H., Al-Otaibi, M., & Nagy, M. S. (2019). Medication compliance and lifestyle adherence in renal transplant recipients in Kuwait. *Patient Preference and Adherence*, *13*, 1477–1486. https://doi.org/10.2147/PPA.S209212
327. Khezerloo, S., Mahmoudi, H., Nia, H. S., & Vafadar, Z. (2019). Predictors of Self-Management among Kidney Transplant Recipients. *Urology Journal*, *16*(4), 366–370. https://doi.org/10.22037/uj.v0i0.5061
328. Khezerloo, S., Mahmoudi, H., & Vafadar, Z. (2019). Evaluating the Psychometric Properties of the Persian Self-Management Scale for Kidney Transplant Recipients. *Urology Journal*, *16*(2), 186–192.
329. Khorassani, F., Tellier, S., & Tsapepas, D. (2019). Pharmacist's Role in Improving Medication Adherence in Transplant Recipients With Comorbid Psychiatric Disorders. *Journal of Pharmacy Practice*, *32*(5), 568–578. https://doi.org/10.1177/0897190018764074
330. Kiley, D. J., Lam, C. S., & Pollak, R. (1993). A study of treatment compliance following kidney transplantation. *Transplantation*, *55*(1), 51–56. https://doi.org/10.1097/00007890-199301000-00010
331. Kim, J., Kim, K., & Jang, I. (2019). Symptom Experience, Self-Care Adherence, and Quality of Life Among Heart Transplant Recipients in South Korea. *Clinical Nursing Research*, *28*(2), 182–201. https://doi.org/10.1177/1054773817740531
332. Kim, S., Ju, M. K., Son, S., Jun, S., Lee, S. Y., & Han, C. S. (2020). Development of video-based educational materials for kidney-transplant patients. *PLoS One*, *15*(8), e0236750. https://doi.org/10.1371/journal.pone.0236750
333. King, C., Dan, W., & Johnstone, S. (2006). Are your patients ready? Empowering patients to create their best possible post-transplant experience. *Nephrology News & Issues*, *20*(10), 54–61.
334. King's College Hospital NHS Trust. (2019). *ExeRTiOn2- The Weight Gain Prevention Exercise in Renal Transplant Online Study. A Feasibility Randomised Controlled Trial.*

(Clinical trial registration NCT03996551). clinicaltrials.gov. https://clinicaltrials.gov/ct2/show/NCT03996551

335. Klaassen, G., Zelle, D. M., Navis, G. J., Dijkema, D., Bemelman, F. J., Bakker, S. J. L., & Corpeleijn, E. (2017). Lifestyle intervention to improve quality of life and prevent weight gain after renal transplantation: Design of the Active Care after Transplantation (ACT) randomized controlled trial. *BMC Nephrology*, *18*. https://doi.org/10.1186/s12882-017-0709-0
    - Corpeleijn, E. (2018). *ACtive Care After Transplantation, a Lifestyle Intervention in Renal Transplant Recipients* (Clinical trial registration NCT01047410). clinicaltrials.gov. https://clinicaltrials.gov/ct2/show/NCT01047410
336. Klein, A., & Kramer, I. (2007). [Influence of a pharmaceutical care program on liver transplant patients' compliance with immunosuppressive therapy: A prospective, randomized, controlled trial]. *Krankenhauspharmazie*, *28*(12), 458–468.
337. Klein, J., Tran, S. N., Mentha-Dugerdil, A., Giostra, E., Majno, P., Morard, I., Berney, T., Dendauw, P., Morel, P., Mentha, G., Iselin, C. E., & Toso, C. (2013). Assessment of sexual function and conjugal satisfaction prior to and after liver transplantation. *Annals of Transplantation*, *18*, 136–145. https://doi.org/10.12659/AOT.883860
338. Klewitz, F., Nöhre, M., Bauer-Hohmann, M., Tegtbur, U., Schiffer, L., Pape, L., Schiffer, M., & de Zwaan, M. (2019). Information Needs of Patients About Immunosuppressive Medication in a German Kidney Transplant Sample: Prevalence and Correlates. *Frontiers in Psychiatry*, *10*, 444. https://doi.org/10.3389/fpsyt.2019.00444
339. Knihs, N. D. S., Sens, S., da Silva, A. M., Wachholz, L. F., Paim, S. M. S., & Magalhaes, A. L. P. (2020). Care transition for liver transplanted patients during the covid-19 pandemic. *Texto & Contexto Enfermagem*, *29*, e20200191. https://doi.org/10.1590/1980-265X-TCE-2020-0191
340. Ko, D., & Bratzke, L. C. (2020). Cognitive Function in Liver Transplant Recipients Who Survived More Than 6 Months. *Progress in Transplantation*, *30*(4), 335–341. https://doi.org/10.1177/1526924820958144
341. Ko, D., Bratzke, L. C., Muehrer, R. J., & Brown, R. L. (2019). Self-management in liver transplantation. *Applied Nursing Research*, *45*, 30–38. https://doi.org/10.1016/j.apnr.2018.11.002
342. Ko, D., Lee, I., & Muehrer, R. J. (2016). Informational needs of liver transplant recipients during a two-year posttransplant period. *Chronic Illness*, *12*(1), 29–40. https://doi.org/10.1177/1742395315601415
343. Ko, D., Muehrer, R. J., & Bratzke, L. C. (2018). Self-Management in Liver Transplant Recipients: A Narrative Review. *Progress in Transplantation*, *28*(2), 100–115. https://doi.org/10.1177/1526924818765814
344. Kobayashi, S., Tsutsui, J., Okabe, S., Hideki, I., Akaho, R., & Nishimura, K. (2020). Medication nonadherence after kidney transplantation: An internet-based survey in Japan. *Psychology, Health & Medicine*, *25*(1), 91–101. https://doi.org/10.1080/13548506.2019.1622745
345. Kobus, G., Malyszko, J., Malyszko, J. S., Puza, E., Bachorzewska-Gajewska, H., & Mysliwiec, M. (2011). Compliance with lifestyle recommendations in kidney allograft recipients. *Transplantation Proceedings*, *43*(8), 2930–2934. https://doi.org/10.1016/j.transproceed.2011.08.031
346. Koller, A., Denhaerynck, K., Moons, P., Steiger, J., Bock, A., & De Geest, S. (2010). Distress associated with adverse effects of immunosuppressive medication in kidney transplant recipients. *Progress in Transplantation*, *20*(1), 40–46. https://doi.org/10.7182/prtr.20.1.1817728501066246
347. Köllner, V., & Archonti, C. (2003). Psychotherapeutische Interventionen vor und nach Organtransplantation. *Verhaltenstherapie*, *13*(1), 47–60. https://doi.org/10.1159/000070499
348. Kong, I. L., & Molassiotis, A. (1999). Quality of life, coping and concerns in Chinese patients after renal transplantation. *International Journal of Nursing Studies*, *36*(4), 313–322. https://doi.org/10.1016/S0020-7489(99)00025-5

349. Konstam, V. (1995). Anger: A neglected group treatment issue with cardiac transplantation recipients and their families. *Journal for Specialists in Group Work*, *20*(3), 189–194. https://doi.org/10.1080/01933929508411343
350. Korb-Savoldelli, V., Sabatier, B., Gillaizeau, F., Guillemain, R., Prognon, P., Begue, D., & Durieux, P. (2010). Non-adherence with drug treatment after heart or lung transplantation in adults: A systematic review. *Patient Education and Counseling*, *81*(2), 148–154. https://doi.org/10.1016/j.pec.2010.04.013
351. Korukcu, O., Boran, O. F., Gungor, O., Boran, M., Bakacak, Z., Bozan, M. B., Calisir, F., Guzel, F. B., & Kutludemirkol, M. (2020). An Underestimated Human Need After Renal Transplantation: Sexuality. *Sexuality and Disability*, *38*(4), 699–714. https://doi.org/10.1007/s11195-020-09647-y
352. Kotarska, K., Wunsch, E., Raszeja-Wyszomirska, J., Kempinska-Podhorodecka, A., Wojcicki, M., & Milkiewicz, P. (2015). Leisure time physical activity and health-related behaviours after liver transplantation: A prospective, single-centre study. *Przeglad Gastroenterologiczny*, *10*(2), 100–104. https://doi.org/10.5114/pg.2015.49002
353. Krajewski, C., & Sucato, G. (2014). Reproductive health care after transplantation. *Best Practice & Research Clinical Obstetrics & Gynaecology*, *28*(8), 1222–1234. https://doi.org/10.1016/j.bpobgyn.2014.09.002
354. Kristensen, I. V., Birkelund, R., Henriksen, J., & Norlyk, A. (2021). Living in limbo while one's identity is changing: Patients' existential experiences 6 months after a kidney transplantation with a living donor. *Journal of Advanced Nursing*, *77*(3), 1403–1410. https://doi.org/10.1111/jan.14683
355. Kugler, C., Fischer, S., Gottlieb, J., Tegtbur, U., Welte, T., Goerler, H., Simon, A., Haverich, A., & Strueber, M. (2007). Symptom experience after lung transplantation: Impact on quality of life and adherence. *Clinical Transplantation*, *21*(5), 590–596. https://doi.org/10.1111/j.1399-0012.2007.00693.x
356. Kugler, C., Fuehner, T., Dierich, M., DeWall, C., Haverich, A., Simon, A., Welte, T., & Gottlieb, J. (2009). Effect of adherence to home spirometry on bronchiolitis obliterans and graft survival after lung transplantation. *Transplantation*, *88*(1), 129–134. https://dx.doi.org/10.1097/TP.0b013e3181aad129
357. Kugler, C., Geyer, S., Gottlieb, J., Simon, A., Haverich, A., & Dracup, K. (2009). Symptom experience after solid organ transplantation. *Journal of Psychosomatic Research*, *66*(2), 101–110. https://doi.org/10.1016/j.jpsychores.2008.07.017
358. Kugler, C., Gottlieb, J., Dierich, M., Haverich, A., Strueber, M., Welte, T., & Simon, A. (2010). Significance of patient self-monitoring for long-term outcomes after lung transplantation. *Clinical Transplantation*, *24*(5), 709–716. https://doi.org/10.1111/j.1399-0012.2009.01197.x
359. Kuhn, W. F., Davis, M. H., & Lippmann, S. B. (1988). Emotional adjustment to cardiac transplantation. *General Hospital Psychiatry*, *10*(2), 108–113. https://doi.org/10.1016/0163-8343(88)90095-3
360. Kumnig, M., Beck, T., Hofer, S., Konig, P., Schneeberger, S., Weisenbacher, A., Bunzel, B., Schusler, G., & Rumpold, G. (2013). [Preoperative evaluation of the body concept of donors and recipients of living kidney donations]. *Zeitschrift für Psychosomatische Medizin und Psychotherapie*, *59*(1), 3–12. https://doi.org/10.13109/zptm.2013.59.1.3
361. Kurz, J. M. (2018). Pregnancy after Solid Organ Transplantation. *American Journal of Maternal Child Nursing*, *43*(2), 89–96. https://doi.org/10.1097/NMC.0000000000000403
362. Kuwaiti, S., Ghadami, A., & Yousefi, H. (2017). Effects of the self-management program on the quality of life among kidney-transplant patients in Isfahan's Hazrat Abolfazl Health and Medical Charity in 2015. *Annals of Tropical Medicine and Public Health*, *10*(6), 1607–1612. https://doi.org/10.4103/Atmph.Atmph_543_17
    - Kuwaiti, S. (2016). *Self-management program impact on the quality of life in patients with kidney transplant* (Clinical Trial Registration IRCT2016062828457N4). Iranian Registry of Clinical Trials. https://en.irct.ir/trial/23096?revision=23096

363. Laederach-Hofmann, K., Bunzel, B., Freundorfer, E., & Schubert, M. T. (2002). [Changes in partner relationship after organ transplantation: Comparison between heart, liver, and kidney transplantation]. *Psychotherapie, Psychosomatik, Medizinische Psychologie*, *52*(1), 5–15. https://doi.org/10.1055/s-2002-19663
364. Langenbach, V. M., Stippel, D., Beckurts, K. T., Geisen, J., & Köhle, K. (2004). [How do patients experience their body after simultaneous pancreas-kidney transplantation?]. *Zeitschrift für Psychosomatische Medizin und Psychotherapie*, *50*(1), 86–102. https://doi.org/10.13109/zptm.2004.50.1.86
365. Langer, D. (2021). Addressing the changing rehabilitation needs of patients undergoing thoracic surgery. *Chronic Respiratory Disease*, *18*, 1479973121994783-undefined. https://doi.org/10.1177/1479973121994783
366. Langer, D., Gosselink, R., Pitta, F., Burtin, C., Verleden, G., Dupont, L., Decramer, M., & Troosters, T. (2009). Physical activity in daily life 1 year after lung transplantation. *Journal of Heart & Lung Transplantation*, *28*(6), 572–578. https://doi.org/10.1016/j.healun.2009.03.007
367. Lanuza, D. M., McCabe, M., Norton-Rosko, M., Corliss, J. W., & Garrity, E. (1999). Symptom experiences of lung transplant recipients: Comparisons across gender, pretransplantation diagnosis, and type of transplantation. *Heart & Lung*, *28*(6), 429–437. https://doi.org/10.1016/s0147-9563(99)70032-4
368. Latos, M., Barabas, K., Lazar, G., Szederkenyi, E., Szenohradszky, P., Marofka, F., & Csabai, M. (2012). Mental representations of the new organ and posttransplant patients' anxiety as related to kidney function. *Transplantation Proceedings*, *44*(7), 2143–2146. https://doi.org/10.1016/j.transproceed.2012.07.097
369. Látos, M., Lázár, G., Ondrik, Z., Szederkényi, E., Hódi, Z., Horváth, Z., & Csabai, M. (2021). Positive psychology intervention to improve recovery after renal transplantation: A randomized controlled trial. *Journal of Contemporary Psychotherapy: On the Cutting Edge of Modern Developments in Psychotherapy*. https://doi.org/10.1007/s10879-021-09515-6
370. Lee, H., Shin, B. C., & Seo, J. M. (2020). Effectiveness of eHealth interventions for improving medication adherence of organ transplant patients: A systematic review and meta-analysis. *PLoS One*, *15*(11). https://doi.org/10.1371/journal.pone.0241857
371. Lee, J. H., Kang, S.-M., Kim, Y. Ah., & Chu, S. H. (2021). Clinical outcomes of a nurse-led post-discharge education program for heart-transplant recipients: A retrospective cohort study. *Applied Nursing Research*, *59*, 151427. https://doi.org/10.1016/j.apnr.2021.151427
372. Lee, S.-Y., Lu, L.-C., Tung, H.-H., Wei, C.-W., Wu, Y.-C., & Liang, S.-Y. (2020). Exploring the Experience of Returning to Society in Heart Transplant Recipients. *Aging Medicine and Healthcare*, *11*(1), 27–34. https://doi.org/10.33879/AMH.2020.035-1901.001
373. Lennerling, A., Kisch, A. M., & Forsberg, A. (2018). Health Literacy Among Swedish Lung Transplant Recipients 1 to 5 Years After Transplantation. *Progress in Transplantation*, *28*(4), 338–342. https://doi.org/10.1177/1526924818800043
374. Lennerling, A., Petersson, I., Andersson, U.-M., & Forsberg, A. (2021). Health Literacy among patients with end-stage kidney disease and kidney transplant recipients. *Scandinavian Journal of Caring Sciences*, *35*(2), 485–491. https://doi.org/10.1111/scs.12860
375. Leung, V. K. Y., Dobbinson, S. J., Goodman, D. J., Kanellis, J., & Chong, A. H. (2018). Skin cancer history, sun-related attitudes, behaviour and sunburn among renal transplant recipients versus general population. *Australasian Journal of Dermatology*, *59*(2), e106–e113. https://dx.doi.org/10.1111/ajd.12591
376. Li, L., Ma, Z., & Wang, W. (2020). Influence of transitional care on the self-care ability of kidney transplant recipients after discharge. *Annals of Palliative Medicine*, *9*(4), 1958–1964. https://doi.org/10.21037/apm-20-1120
377. Li-Chueh, W., Yu-Tzu, D., Yi-Wen, W., Hsiu-Li, H., & Yang-Jen, C. (2008). Effects of self-efficacy, self-care behaviours on depressive symptom of Taiwanese kidney

transplant recipients. *Journal of Clinical Nursing*, *17*(13), 1786–1794. https://doi.org/10.1111/j.1365-2702.2007.02035.x
378. Lieb, M., Schiffer, M., & Erim, Y. (2020). Optimization of Electronically Monitored Non-Adherence in Highly Adherent Renal Transplant Recipients by Reducing the Dosing Frequency—A Prospective Single-Center Observational Study. *Patient Preference and Adherence*, *14*, 1389–1401. https://doi.org/10.2147/PPA.S258131
379. Lieb, M., Weyand, M., Seidl, M., & Erim, Y. (2020). Prospective single-centre clinical observational study on electronically monitored medication non-adherence, its psychosocial risk factors and lifestyle behaviours after heart transplantation: A study protocol. *BMJ Open*, *10*(10), e038637. https://dx.doi.org/10.1136/bmjopen-2020-038637
    - Universitätsklinikum Erlangen. (2020). Eine prospektive monozentrische Studie zur elektronisch gemessenen medikamentösen Non-Adhärenz, Psychosozialen Risikofaktoren und Lebensstilen nach einer Herztransplantation (Clinical trial registration DRKS00020496). German Clinical Trials Register [DRKS]. https://www.drks.de/drks_web/navigate.do?navigationId=trial.HTML&TRIAL_ID=DRKS00020496
380. Lieber, S. R., Kim, H. P., Baldelli, L., Nash, R., Teal, R., Magee, G., Loiselle, M. M., Desai, C. S., Lee, S. C., Singal, A. G., Marrero, J. A., Barritt, A. S. th, & Evon, D. M. (2021). What Survivorship Means to Liver Transplant Recipients: Qualitative Groundwork for a Survivorship Conceptual Model. *Liver Transplantation*, *27*(10), 1454–1467. https://dx.doi.org/10.1002/lt.26088
381. Lin, C. S., Wang, S. S., Chang, C. L., & Shih, F. J. (2010). Dark-recovery experiences, coping strategies, and needs of adult heart transplant recipients in Taiwan. *Transplantation Proceedings*, *42*(3), 940–942. https://doi.org/10.1016/j.transproceed.2010.03.021
382. Lin, I. H., Wong, T. C., Nien, S. W., Chou, Y. T., Chiang, Y. J., Wang, H. H., & Yang, S. H. (2019). Dietary Compliance Among Renal Transplant Recipients: A Single-Center Study in Taiwan. *Transplantation Proceedings*, *51*(5), 1325–1330. https://doi.org/10.1016/j.transproceed.2019.02.026
383. Lin, S.-Y., Fetzer, S. J., Lee, P.-C., & Chen, C.-H. (2011). Predicting adherence to health care recommendations using health promotion behaviours in kidney transplant recipients within 1-5 years post-transplant. *Journal of Clinical Nursing*, *20*(23–24), 3313–3321. https://doi.org/10.1111/j.1365-2702.2011.03757.x
384. Lindberg, C., Almgren, M., Lennerling, A., & Forsberg, A. (2020). The Meaning of Surviving Three Years after a Heart Transplant—A Transition from Uncertainty to Acceptance through Adaptation. *International Journal of Environmental Research and Public Health*, *17*(15), Art. 15. https://doi.org/10.3390/ijerph17155434
385. Lindqvist, R., Carlsson, M., & Sjödén, P. (2004). Coping strategies of people with kidney transplants. *Journal of Advanced Nursing*, *45*(1), 47–52. https://doi.org/10.1046/j.1365-2648.2003.02859.x
386. Lindup, M., van den Bogaart, L., Golshayan, D., Aubert, J. D., Vionnet, J., Regamey, J., Pascual, M., Manuel, O., & Mombelli, M. (2020). Real-life food-safety behavior and incidence of foodborne infections in solid organ transplant recipients. *American Journal of Transplantation*, *20*(5), 1424–1430. https://doi.org/10.1111/ajt.15725
387. Lisson, G. L., Rodrigue, J. R., Reed, A. I., & Nelson, D. R. (2005). A brief psychological intervention to improve adherence following transplantation. *Annals of Transplantation*, *10*(1), 52–57.
388. Liu, J., Zhu, X., Yan, J., Gong, L., Wu, X., Liu, M., & Mao, P. (2021). Association Between Regulatory Emotional Self-Efficacy and Immunosuppressive Medication Adherence in Renal Transplant Recipients:Does Medication Belief Act as a Mediator? *Frontiers in Pharmacology*, *12*. https://doi.org/10.3389/fphar.2021.559368
389. Liu, M., Sun, Q., Cui, L., Liu, J., Liu, L., Wu, X., Wang, Z., Zhou, X., Xie, J., & Cheng, A. S. (2021). Fear of Movement and Physical Self-Efficacy Partially Mediate the Association Between Fatigue and Physical Activity Among Kidney Transplant

Recipients. *Clinical Nursing Research*, *30*(7), 950–959. https://dx.doi.org/10.1177/1054773821990263
390. Loescher, L. J., Hansen, C., Hepworth, J. T., Quale, L., & Sligh, J. (2013). A Preliminary Study of a Video Intervention to Inform Solid Organ Transplant Recipients About Skin Cancer. *Transplantation Proceedings*, *45*(9), 3187–3189. https://doi.org/10.1016/j.transproceed.2012.09.118
391. Loghmain-Adham, M. (2003). Medication noncompliance in patients with chronic disease: Issues in dialysis and renal transplantation. *American Journal of Managed Care*, *9*(2), 155–171.
392. Lorenz, E. C., Egginton, J. S., Stegall, M. D., Cheville, A. L., Heilman, R. L., Nair, S. S., Mai, M. L., & Eton, D. T. (2019). Patient experience after kidney transplant: A conceptual framework of treatment burden. *Journal of Patient-Reported Outcomes*, *3*(8), 1–9. https://doi.org/10.1186/s41687-019-0095-4
393. Low, E. S. L., Gow, P. J., Testro, A., & Sinclair, M. (2021). Low participation in preventative health measures in a cohort of liver transplant recipients: A cross-sectional analysis. *Clinical Transplantation*, *35*(5). https://doi.org/10.1111/ctr.14257
394. Low, J. K., Crawford, K., Manias, E., & Williams, A. (2016). A compilation of consumers' stories: The development of a video to enhance medication adherence in newly transplanted kidney recipients. *Journal of Advanced Nursing*, *72*(4), 813–824. https://doi.org/10.1111/jan.12886
395. Low, J. K., Crawford, K., Manias, E., & Williams, A. (2017). Stressors and coping resources of Australian kidney transplant recipients related to medication taking: A qualitative study. *Journal of Clinical Nursing*, *26*(11–12), 1495–1507. https://dx.doi.org/10.1111/jocn.13435
396. Low, J. K., Williams, A., Manias, E., & Crawford, K. (2015). Interventions to improve medication adherence in adult kidney transplant recipients: A systematic review. *Nephrology Dialysis Transplantation*, *30*(5), 752–761. https://doi.org/10.1093/ndt/gfu204
397. Lucey, M. R. (2017). Impact of behaviors (smoking, treatment adherence, exercise, alcohol) on allograft function and outcomes. *Liver Transplantation*, *23*(S1), S89–S91. https://dx.doi.org/10.1002/lt.24861
398. Luk, W. S. (2004). The HRQoL of renal transplant patients. *Journal of Clinical Nursing*, *13*(2), 201–209. https://doi.org/10.1046/j.1365-2702.2003.00867.x
399. Lundmark, M., Erlandsson, L.-K., Lennerling, A., Almgren, M., & Forsberg, A. (2016). Health transition after lung transplantation – a grounded theory study. *Journal of Clinical Nursing*, *25*(15–16), 2285–2294. https://doi.org/10.1111/jocn.13269
400. Lundmark, M., Lennerling, A., Almgren, M., & Forsberg, A. (2016). Recovery after lung transplantation from a patient perspective—Proposing a new framework. *Journal of Advanced Nursing*, *72*(12), 3113–3124. https://dx.doi.org/10.1111/jan.13058
401. Lundmark, M., Lennerling, A., Almgren, M., & Forsberg, A. (2019). Recovery, symptoms, and well-being one to five years after lung transplantation—A multi-centre study. *Scandinavian Journal of Caring Sciences*, *33*(1), 176–184. https://dx.doi.org/10.1111/scs.12618
402. Lundmark, M., Lennerling, A., & Forsberg, A. (2019). Developing a Grounded Theory on Adaptation After Lung Transplantation From Intermediate-Term Patient Experiences. *Progress in Transplantation*, *29*(2), 135–143. https://doi.org/10.1177/1526924819835823
403. Maasdam, L., Timman, R., Cadogan, M., Tielen, M., van Buren, M. C., Weimar, W., & Massey, E. K. (2022). Exploring health literacy and self-management after kidney transplantation: A prospective cohort study. *Patient Education and Counseling*, *105*(2), 440–446. https://doi.org/10.1016/j.pec.2021.05.013
404. Madziarska, K., Hap, K., Mazanowska, O., & Sutkowska, E. (2021). Comprehensive lifestyle modification as complementary therapy to prevent and manage post-transplant diabetes mellitus. *Postepy Higieny I Medycyny Doswiadczalnej*, *75*, 238–245. https://doi.org/10.5604/01.3001.0014.8311

405. Mangold, K. (2016). Utilization of the Simulation Environment to Practice Teach-Back With Kidney Transplant Patients. *Clinical Simulation in Nursing*, *12*(12), 532–538. https://doi.org/10.1016/j.ecns.2016.08.004
406. Mansell, H., Rosaasen, N., West-Thielke, P., Wichart, J., Daley, C., Mainra, R., Shoker, A., Liu, J., & Blackburn, D. (2019). Randomised controlled trial of a video intervention and behaviour contract to improve medication adherence after renal transplantation: The VECTOR study protocol. *BMJ Open*, *9*(3), e025495-undefined. https://dx.doi.org/10.1136/bmjopen-2018-025495
    - Mansell, H. (2018). *Improving Adherence in Kidney Recipients: A Randomized Controlled Trial of a Post-transplant Education Intervention* (Clinical trial registration NCT03540121). clinicaltrials.gov. https://clinicaltrials.gov/ct2/show/NCT03540121
407. Marcelino, C. A. G., Díaz, L. J. R., & da Cruz, D. M. (2015). The effectiveness of interventions in managing treatment adherence in adult heart transplant patients: A systematic review. *JBI Database of Systematic Reviews and Implementation Reports*, *13*(9), 279–308. https://doi.org/10.11124/jbisrir-2015-2288
408. Marcinkowski, A., Ziebolz, D., Kleibrink, B. E., Weinreich, G., Kamler, M., Teschler, H., & Sommerwerck, U. (2018). Deficits in oral health behavior and oral health status in patients after lung transplantation. *The Clinical Respiratory Journal*, *12*(2), 721–730. https://doi.org/10.1111/crj.12585
409. Maroney, K., Curtis, L. M., Opsasnick, L., Smith, K. D., Eifler, M. R., Moore, A., Wedd, J., Wolf, M. S., & Patzer, R. E. (2021). EHealth literacy and web-based patient portal usage among kidney and liver transplant recipients. *Clinical Transplantation*, *35*(2), e14184. https://doi.org/10.1111/ctr.14184
410. Marsicano-Souza, E. O., Colugnati, F., Geest, S. D., & Sanders-Pinheiro, H. (2021). Nonadherence to immunosuppressives and treatment in kidney transplant: ADHERE BRAZIL Study. *Revista de Saude Publica*, *55*, 33–33. https://doi.org/10.11606/s1518-8787.2021055002894
411. Martell, J., Rice, E. I., Crooks, N. K., Ko, D., & Muehrer, R. J. (2015). What are patients saying about sex after a kidney or simultaneous kidney/pancreas transplant? *Progress in Transplantation*, *25*(3), 251–256. https://doi.org/10.7182/pit2015912
412. Martin, J. C., Hathaway, D. K., Egidi, M. F., & Gaber, A. O. (2001). Lifestyle behaviors affect cardiovascular risk status in men 1 year after kidney transplantation. *Clinical Transplantation*, *15 Suppl 6*, 41–45. https://doi.org/10.1034/j.1399-0012.2001.00007.x
413. Masajtis-Zagajewska, A., Muras, K., & Nowicki, M. (2019). Effects of a Structured Physical Activity Program on Habitual Physical Activity and Body Composition in Patients With Chronic Kidney Disease and in Kidney Transplant Recipients. *Experimental and Clinical Transplantation*, *17*(2), 155–164. https://doi.org/10.6002/ect.2017.0305
414. Massey, E. K., Meys, K., Kerner, R., Weimar, W., Roodnat, J., & Cransberg, K. (2015). Young Adult Kidney Transplant Recipients: Nonadherent and Happy. *Transplantation*, *99*(8), e89-96. https://dx.doi.org/10.1097/TP.0000000000000639
415. Massey, E. K., Tielen, M., Laging, M., Beck, D. K., Khemai, R., van Gelder, T., & Weimar, W. (2013). The role of goal cognitions, illness perceptions and treatment beliefs in self-reported adherence after kidney transplantation: A cohort study. *Journal of Psychosomatic Research*, *75*(3), 229–234. https://doi.org/10.1016/j.jpsychores.2013.07.006
416. Massey, E. K., Tielen, M., Laging, M., Timman, R., Beck, D. K., Khemai, R., van Gelder, T., & Weimar, W. (2015). Discrepancies between beliefs and behavior: A prospective study into immunosuppressive medication adherence after kidney transplantation. *Transplantation*, *99*(2), 375–380. https://doi.org/10.1097/TP.0000000000000608
417. Massierer, D., Sapir-Pichhadze, R., Bouchard, V., Dasgupta, K., Fernandez, N., Costa, D. da, Ahmed, S., Fortin, M.-C., Langevin, R., Mayo, N., & Janaudis-Ferreira, T. (2019). Web-Based Self-Management Guide for Kidney Transplant Recipients (The Getting on With Your Life With a Transplanted Kidney Study): Protocol for

Development and Preliminary Testing. *JMIR Research Protocols*, *8*(6), e13420. https://doi.org/10.2196/13420
418. Matteson-Kome, M. L., Ruppar, T., & Russell, C. (2016). Attainment of the Elusive: Attributions for Long-term Success in Kidney Transplantation. *Progress in Transplantation*, *26*(2), 162–171. https://doi.org/10.1177/1526924816640665
419. Matthees, B. J., Anantachoti, P., Kreitzer, M. J., Savik, K., Hertz, M. I., & Gross, C. R. (2001). Use of complementary therapies, adherence, and quality of life in lung transplant recipients. *Heart & Lung*, *30*(4), 258–268. https://doi.org/10.1067/mhl.2001.116135
420. Maurer, G., & Abriola, D. (1994). Pregnancy following renal transplant. *The Journal of Perinatal & Neonatal Nursing*, *8*(1), 28–36. https://doi.org/10.1097/00005237-199406000-00005
421. Mauthner, O., De Luca, E., Poole, J., Gewarges, M., Abbey, S. E., Shildrick, M., & Ross, H. (2012). Preparation and Support of Patients through the Transplant Process: Understanding the Recipients' Perspectives. *Nursing Research and Practice*, *2012*, e547312. https://doi.org/10.1155/2012/547312
422. Mazzoni, D., Cicognani, E., Mosconi, G., Totti, V., Roi, G. S., Trerotola, M., & Nanni Costa, A. (2014). Sport activity and health-related quality of life after kidney transplantation. *Transplantation Proceedings*, *46*(7), 2231-2234-undefined. https://doi.org/10.1016/j.transproceed.2014.07.049
423. McGillicuddy, J. W., Chandler, J. L., Sox, L. R., & Taber, D. J. (2020). Exploratory Analysis of the Impact of an mHealth Medication Adherence Intervention on Tacrolimus Trough Concentration Variability: Post Hoc Results of a Randomized Controlled Trial. *Annals of Pharmacotherapy*, *54*(12), 1185–1193. https://doi.org/10.1177/1060028020931806
    - McGillicuddy, J., Chandler, J., Sox, L., Mueller, M., Nemeth, L., Baliga, P., & Treiber, F. (2019). „Smartphone Medication Adherence Saves Kidneys“ for Kidney Transplantation Recipients: Protocol for a Randomized Controlled Trial. *JMIR Research Protocols*, *8*(6), e13351. https://dx.doi.org/10.2196/13351
    - Medical University of South Carolina. (2016). *Smart Phone Medication Adherence Saves Kidneys—SMASK* (Clinical trial registration NCT02827695). clinicaltrials.gov. https://clinicaltrials.gov/ct2/show/NCT02827695
424. McGillicuddy, J. W., Taber, D. J., Mueller, M., Patel, S., Baliga, P. K., Chavin, K. D., Sox, L., Favela, A. P., Brunner-Jackson, B. M., & Treiber, F. A. (2015). Sustainability of improvements in medication adherence through a mobile health intervention. *Progress in Transplantation*, *25*(3), 217–223. https://doi.org/10.7182/pit2015975
    - McGillicuddy, J. W., Gregoski, M. J., Weiland, A. K., Rock, R. A., Brunner-Jackson, B. M., Patel, S. K., Thomas, B. S., Taber, D. J., Chavin, K. D., Baliga, P. K., & Treiber, F. A. (2013). Mobile Health Medication Adherence and Blood Pressure Control in Renal Transplant Recipients: A Proof-of-Concept Randomized Controlled Trial. *JMIR research protocols*, *2*(2), e32. https://doi.org/10.2196/resprot.2633
425. McKay, S. C., Lembach, H., Hann, A., Okoth, K., Anderton, J., Nirantharakumar, K., Magill, L., Torlinska, B., Armstrong, M., Mascaro, J., Inston, N., Pinkney, T., Ranasinghe, A., Borrows, R., Ferguson, J., Isaac, J., Calvert, M., Perera, T., & Hartog, H. (2021). Health-related quality of life, uncertainty and coping strategies in solid organ transplant recipients during shielding for the COVID-19 pandemic. *Transplant International*, *11*, 11-undefined. https://dx.doi.org/10.1111/tri.14010
426. McKie, P., Webzell, I., Tavabie, O., Loewenthal, D., & Heaton, N. (2020). An exploratory study of the experiences of deceased-donor liver transplant recipients and their need for psychotherapeutic support. *Journal of Clinical Nursing*, *29*(15–16), 2991–2998. https://doi.org/10.1111/jocn.15309
427. McPake, D., & Burnapp, L. (2009). Caring for patients after kidney transplantation. *Nursing Standard*, *23*(19), 49–57; quiz 58. https://doi.org/10.7748/ns2009.01.23.19.49.c6744

428. Medical University of Graz, & Schemmer, P. (2020). *Improving ImmunoSuppression Adherence After Liver or Kidney Transplantation -a Randomized Controlled Single Centre Trial* (Clinical trial registration NCT04207125). clinicaltrials.gov. https://clinicaltrials.gov/ct2/show/NCT04207125
429. Medizinische Hochschule Hannover. (2019). *Verhaltens- und ernährungsmedizinische Intervention zur Gewichtsreduktion bei erwachsenen nierentransplantierten Patienten mit Übergewicht oder Adipositas* (Clinical trial registration DRKS00017226). German Clinical Trials Register [DRKS]. https://www.drks.de/drks_web/navigate.do?navigationId=trial.HTML&TRIAL_ID=DRKS00017226
430. Mersal, F. A. (2014). Effect of Patient Education on Coping, Quality of Life, Knowledge and Self Efficacy of Kidney Recipient Patients. *American Journal of Nursing Science*, *3*(5). https://doi.org/10.11648/j.ajns.20140305.14
431. Migliozzi, D. R., Zullo, A. R., Collins, C., & Elsaid, K. A. (2015). Achieving blood pressure control among renal transplant recipients by integrating electronic health technology and clinical pharmacy services. *American Journal of Health-System Pharmacy*, *72*(22), 1987–1992. https://doi.org/10.2146/ajhp140810
432. Mihalis, E. L., Wysong, A., Boscardin, W. J., Tang, J. Y., Chren, M. M., & Arron, S. T. (2013). Factors affecting sunscreen use and sun avoidance in a U.S. national sample of organ transplant recipients. *British Journal of Dermatology*, *168*(2), 346–353. https://doi.org/10.1111/j.1365-2133.2012.11213.x
433. Milaniak, I., Wilczek Ruzyczka, E., & Przybylowski, P. (2021). Mediating effect of coping strategies on the relation between social support and depressive symptoms among patients after cardiac transplantation. *Psychiatria Polska*, *55*(2), 331–344. https://doi.org/10.12740/PP/118055
434. Milaniak, I., Wilczek-Ruzyczka, E., Wierzbicki, K., Sadowski, J., Kapelak, B., & Przybylowski, P. (2016). Role of Personal Resources in Depression and Stress in Heart Transplant Recipients. *Transplantation Proceedings*, *48*(5), 1761–1766. https://doi.org/10.1016/j.transproceed.2016.01.080
435. Moayed, M. S., Ebadi, A., Khodaveisi, M., Toosi, M. N., Soltanian, A. R., & Khatiban, M. (2018). Factors influencing health self-management in adherence to care and treatment among the recipients of liver transplantation. *Patient Preference and Adherence*, *12*, 2425–2436. https://doi.org/10.2147/PPA.S180341
436. Moayed, M. S., Khatiban, M., Toosi, M. N., Khodaveisi, M., Soltanian, A. R., & Ebadi, A. (2019). Barriers to Adherence to Medical Care Programs in Liver Transplant Recipients: A Qualitative Study. *International Journal of Organ Transplantation Medicine*, *10*(3), 115–126.
437. Mohamed, M., Soliman, K., Pullalarevu, R., Kamel, M., Srinivas, T., Taber, D., & Posadas Salas, M. A. (2021). Non-Adherence to Appointments is a Strong Predictor of Medication Non-Adherence and Outcomes in Kidney Transplant Recipients. *American Journal of the Medical Sciences*, *362*(4), 381–386. https://doi.org/10.1016/j.amjms.2021.05.011
438. Mohney, K. (2018). Learning to Live Again: The Role of Education in Heart Transplant Recipients. *Critical Care Nursing Quarterly*, *41*(4), 389–393. https://doi.org/10.1097/CNQ.0000000000000225
439. Mollazadeh, F., & Hemmati Maslakpak, M. (2018). The Effect of Teach-Back Training on Self Management in Kidney Transplant Recipients: A Clinical Trial. *International Journal of Community Based Nursing and Midwifery*, *6*(2), 146–155.
    - Maslakpak, M. H. (2017). *Effect of education with teach–back method on self-care among renal transplant recipients* (Clinical trial registration IRCT2016122817059N10). Iranian Registry of Clinical trials (IRCT). http://www.who.int/trialsearch/Trial2.aspx?TrialID=IRCT2016122817059N10
440. Monemian, S., Abedi, H., & Naji, S. A. (2015). Life experiences in heart transplant recipients. *Journal of Education and Health Promotion*, *4*(1), 18. https://doi.org/10.4103/2277-9531.154037

441. Morales, J. M., Varo, E., & Lazaro, P. (2012). Immunosuppressant treatment adherence, barriers to adherence and quality of life in renal and liver transplant recipients in Spain. *Clinical Transplantation*, *26*(2), 369–376. https://doi.org/10.1111/j.1399-0012.2011.01544.x
442. Morlion, B., Verbandt, Y., Paiva, M., Estenne, M., Michils, A., Sandron, P., Bawin, C., & Assis-Arantes, P. (1999). A telemanagement system for home follow-up of respiratory patients. *IEEE Engineering in Medicine and Biology Magazine*, *18*(4), 71–79. https://doi.org/10.1109/51.775491
443. Moro, J. A., Almenar, L., Martnez-Dolz, L., Aguero, J., Sanchez-Lazaro, I., Iglesias, P., Igual, V., & Salvador, A. (2008). Support program for heart transplant patients: Initial experience. *Transplantation Proceedings*, *40*(9), 3039–3040. https://doi.org/10.1016/j.transproceed.2008.08.101
444. Moscarelli, L., Sofi, F., Mascherini, G., Bini, V., Ingletto, C., Mandoli, M., Galanti, G., & Stefani, L. (2018). Metabolic Profile and Myocardial Performance of Renal Transplant Recipients Participating in Unsupervised Physical Exercise as a Prescription Program. *Journal of Functional Morphology & Kinesiology*, *3*(3), 11-undefined. https://doi.org/10.3390/jfmk3030046
445. Mouelhi, Y., Alessandrini, M., Pauly, V., Dussol, B., & Gentile, S. (2017). Internet and social network users' profiles in Renal Transplant Recipients in France. *BMC Nephrology*, *18*, 1–8. https://doi.org/10.1186/s12882-017-0670-y
446. Muduma, G., Shupo, F. C., Dam, S., Hawken, N. A., Aballea, S., Odeyemi, I., & Toumi, M. (2016). Patient survey to identify reasons for non-adherence and elicitation of quality of life concepts associated with immunosuppressant therapy in kidney transplant recipients. *Patient Preference and Adherence*, *10*, 27–36. https://doi.org/10.2147/PPA.S96086
447. Muehrer, R. J., Keller, M. L., Powwattana, A., & Pornchaikate, A. (2006). Sexuality Among Women Recipients of a Pancreas and Kidney Transplant. *Western Journal of Nursing Research*, *28*(2), 137–150. https://doi.org/10.1177/0193945905283372
448. Muehrer, R. J., Lanuza, D. M., Brown, R. L., & Djamali, A. (2014). Sexual concerns among kidney transplant recipients. *Clinical Transplantation*, *28*(11), 1294–1302. https://doi.org/10.1111/ctr.12454
449. Murray, K. R., Foroutan, F., Amadio, J. M., Posada, J. D., Kozuszko, S., Duhamel, J., Tsang, K., Farkouh, M. E., McDonald, M., Billia, F., Barber, E., Hershman, S. G., Bhat, M., Tinckam, K. J., Ross, H. J., McIntosh, C., & Moayedi, Y. (2021). Remote Mobile Outpatient Monitoring in Transplant (Reboot) 2.0: Protocol for a Randomized Controlled Trial. *JMIR Research Protocols*, *10*(10), e26816. https://doi.org/10.2196/26816
    - Ross, H. (2021). *REmote moBile Outpatient mOnitoring in Transplant (Reboot) 2.0* (Clinical trial registration NCT04721288). clinicaltrials.gov. https://clinicaltrials.gov/ct2/show/NCT04721288
450. Myaskovsky, L., Jesse, M. T., Kuntz, K., Leino, A. D., Peipert, J. D., Russell, C. L., Spivey, C. A., Sulejmani, N., & Dew, M. A. (2018). Report from the American Society of Transplantation Psychosocial Community of Practice Adherence Task Force: Real-world options for promoting adherence in adult recipients. *Clinical Transplantation*, *32*(9), e13353. https://doi.org/10.1111/ctr.13353
451. Myers, J., & Pellino, T. A. (2009). Developing New Ways to Address Learning Needs of Adult Abdominal Organ Transplant Recipients. *Progress in Transplantation*, *19*(2), 160–166. https://doi.org/10.1177/152692480901900210
452. Nadel, C., & Clark, J. J. (1986). Psychosocial adjustment after renal retransplants. *General Hospital Psychiatry*, *8*(1), 41–48. https://doi.org/10.1016/0163-8343(86)90063-0
453. Nagib, A. M., Elsayed Matter, Y., Gheith, O. A., Refaie, A. F., Othman, N. F., & Al-Otaibi, T. (2019). Diabetic Nephropathy Following Posttransplant Diabetes Mellitus. *Experimental and Clinical Transplantation*, *17*(2), 138–146. https://doi.org/10.6002/ect.2018.0157

454. Nashan, D., Radny, P., Kosters, N. C., & Nashan, B. (2007). [Skin tumors in organ-transplant recipients]. *Hautarzt*, *58*(1), 48-+. https://doi.org/10.1007/s00105-006-1159-y
455. Ndemera, H., & Bhengu, B. (2018). Perceptions of healthcare professionals regarding self-management by kidney transplant recipients in South Africa: A qualitative study. *International Journal of Africa Nursing Sciences*, *9*, 120–128. https://doi.org/10.1016/j.ijans.2018.10.003
456. Nerini, E., Bruno, F., Citterio, F., & Schena, F. P. (2016). Nonadherence to immunosuppressive therapy in kidney transplant recipients: Can technology help? *Journal of Nephrology*, *29*(5), 627–636. https://doi.org/10.1007/s40620-016-0273-x
457. Neves da Mota, L. A., Sousa Cruz, M. A., & Oliveira Costa, C. A. (2016). Therapeutic self-management—Development of a flowchart to support decision-making: Qualitative study. *Revista de Enfermagem Referência*, *4*(11), 71–78. https://doi.org/10.12707/RIV16056
458. Nevins, T. E., Robiner, W. N., & Thomas, W. (2014). Predictive Patterns of Early Medication Adherence in Renal Transplantation. *Transplantation*, *98*(8), 878–884. https://doi.org/10.1097/tp.0000000000000148
459. Newton, S. E. (1999). Promoting adherence to transplant medication regimens: A review of behavioral analysis. *Journal of Transplant Coordination*, *9*(1), 13–16. https://doi.org/10.1177/090591999900900102
460. Newton, S. E. (2007). Alcohol relapse and its relationship to the lived experience of adult liver transplant recipients. *Gastroenterology Nursing*, *30*(1), 37–42. https://doi.org/10.1097/00001610-200701000-00004
461. Neyhart, C. D. (2008). Education of Patients Pre and Post-Transplant: Improving Outcomes by Overcoming the Barriers. *Nephrology Nursing Journal*, *35*(4), 409–410.
462. Nicholls, P. H. (1990). Cultural considerations in teaching the Saudi Arabian renal transplant recipient. *ANNA Journal*, *17*(5), 377–380.
463. Nickel, R., Egle, U. T., Wunsch, A., Lohse, A. W., & Otto, G. (2002). [Coping in patients after liver transplantation with regard to their membership in a self-group]. *Zeitschrift Fur Gastroenterologie*, *40*(5), 285–290. https://doi.org/10.1055/s-2002-30117
464. Nielsen, C., Agerskov, H., Bistrup, C., & Clemensen, J. (2019). „The hospital and everyday life are two worlds“: Patients’ and healthcare professionals’ experiences and perspectives on collaboration in the kidney transplantation process. *Nursing Open*, *6*(4), 1491–1500. https://doi.org/10.1002/nop2.349
465. Nielsen, C., Agerskov, H., Bistrup, C., & Clemensen, J. (2020a). Evaluation of a telehealth solution developed to improve follow-up after kidney Transplantation. *Journal of Clinical Nursing*, *29*(7–8), 1053–1063. https://doi.org/10.1111/jocn.15178
466. Nielsen, C., Agerskov, H., Bistrup, C., & Clemensen, J. (2020b). User involvement in the development of a telehealth solution to improve the kidney transplantation process: A participatory design study. *Health Informatics Journal*, *26*(2), 1237–1252. https://doi.org/10.1177/1460458219876188
467. Nielsen, C., Clemensen, J., Bistrup, C., & Agerskov, H. (2019). Balancing everyday life-Patients’ experiences before, during and four months after kidney transplantation. *Nursing Open*, *6*(2), 443–452. https://doi.org/10.1002/nop2.225
468. Nikoogoftar, M., & Shahini, Z. (2021). The effects of resilience training on self-efficacy, empowerment, and social adjustment of renal transplant patients. *Journal of Nursing and Midwifery Sciences*, *8*(3), 137–144. http://dx.doi.org/10.4103/jnms.jnms_173_20
469. Nilsson, M., Forsberg, A., Lennerling, A., & Persson, L.-O. (2013). Coping in relation to perceived threat of the risk of graft rejection and Health- Related Quality of Life of organ transplant recipients. *Scandinavian Journal of Caring Sciences*, *27*(4), 935–944. https://doi.org/10.1111/scs.12007
470. Nöhre, M., Erim, Y., Vitinius, F., Klewitz, F., Schiffer, M., & de Zwaan, M. (2018). [Adherence to immunosuppressive medication following organ transplantation]. *Psychotherapie, Psychosomatik, Medizinische Psychologie*, *68*(5), 185–194. https://doi.org/10.1055/s-0044-100404

471. Northumbria University. (2019). *Efficacy of Physical Activity Tele-coaching to Optimise Daily Physical Activity Levels in Lung Transplant Recipients* (Clinical trial registration NCT03873597). clinicaltrials.gov. https://clinicaltrials.gov/ct2/show/NCT03873597
472. Nour, N., Heck, C., & Ross, H. (2015). Factors Related to Participation in Paid Work After Organ Transplantation: Perceptions of Kidney Transplant Recipients. *Journal of Occupational Rehabilitation*, *25*(1), 38–51. https://doi.org/10.1007/s10926-014-9519-4
473. Nowicka, M., Gorska, M., Nowicka, Z., Edyko, K., Gozdzik, M., & Kurnatowska, I. (2021). Adherence to Pharmacotherapy and Lifestyle Recommendations Among Hemodialyzed Patients and Kidney Transplant Recipients. *Journal of Renal Nutrition*, *31*(5), 503–511. https://doi.org/10.1053/j.jrn.2020.12.006
474. O'Brien, T. (2016). An Integrative Literature Review of Physical Activity Recommendations for Adult Renal Transplant Recipients. *Progress in Transplantation*, *26*(4), 381–385. https://doi.org/10.1177/1526924816664079
475. O'Brien, T., & Rosenthal, A. (2020). Preferred Features in Mobile Health Applications for Kidney Transplant Recipients: A Qualitative Approach. *Nephrology Nursing Journal*, *47*(6), 529–536. https://doi.org/10.37526/1526-744X.2020.47.6.529
476. O'Brien, T., Russell, C. L., Tan, A., Washington, M., & Hathaway, D. (2018). An Exploratory Correlational Study in the Use of Mobile Technology Among Adult Kidney Transplant Recipients. *Progress in Transplantation*, *28*(4), 368–375. https://doi.org/10.1177/1526924818800051
477. O'Carroll, R. E., McGregor, L. M., Swanson, V., Masterton, G., & Hayes, P. C. (2006). Adherence to medication after liver transplantation in Scotland: A pilot study. *Liver Transplantation*, *12*(12), 1862–1868. https://doi.org/10.1002/lt.20828
478. O'Connor, E. M., Koufaki, P., Mercer, T. H., Lindup, H., Nugent, E., Goldsmith, D., Macdougall, I. C., & Greenwood, S. A. (2017). Long-term pulse wave velocity outcomes with aerobic and resistance training in kidney transplant recipients—A pilot randomised controlled trial. *PLoS One*, *12*(2), e0171063-undefined. https://doi.org/10.1371/journal.pone.0171063
479. O'Grady, J. G., Asderakis, A., Bradley, R., Burnapp, L., McPake, D. M., Perrin, M., Russell, S., Watson, A. R., Watson, C. J., Wray, J., & Wilson, L. C. (2010). Multidisciplinary insights into optimizing adherence after solid organ transplantation. *Transplantation*, *89*(5), 627–632. https://doi.org/10.1097/TP.0b013e3181ca87b0
480. O'Kane, M. J. (2020). Patient self-testing in chronic disease management. *Journal of Laboratory Medicine*, *44*(2), 81–87. https://doi.org/10.1515/labmed-2019-0175
481. O'Moore, B. (1999). Regular exercise: Vitally important for the transplant recipient. *Advances in Renal Replacement Therapy*, *6*(2), 187–188. https://doi.org/10.1016/s1073-4449(99)70050-6
482. Ong, S. C., & Rhee, C. M. (2021). Novel management of diabetes in kidney transplantation. *Current Opinion in Nephrology & Hypertension*, *30*(1), 5–13. https://doi.org/10.1097/MNH.0000000000000665
483. Orazio, L., Armstrong, K., Banks, M., Johnson, D., Isbeli, N., & Hickman, I. (2007). Central obesity is common in renal transplant recipients and is associated with increased prevalence of cardiovascular risk factors. *Nutrition & Dietetics*, *64*(3), 200–206. https://doi.org/10.1111/j.1747-0080.2007.00151.x
484. Orazio, L. K., Isbel, N. M., Armstrong, K. A., Tarnarskyj, J., Johnson, D. W., Hale, R. E., Kaisar, M., Banks, M. D., & Hickman, I. J. (2011). Evaluation of dietetic advice for modification of cardiovascular disease risk factors in renal transplant recipients. *Journal of Renal Nutrition*, *21*(6), 462–471. https://doi.org/10.1053/j.jrn.2010.12.002
485. Ordin, Y. S., & Karayurt, O. (2016). Effects of a Support Group Intervention on Physical, Psychological, and Social Adaptation of Liver Transplant Recipients. *Experimental and Clinical Transplantation*, *14*(3), 329–337. https://doi.org/10.6002/ect.2014.0220
486. Ordin, Y. S., Karayurt, O., & Wellard, S. (2013). Investigation of adaptation after liver transplantation using Roy's Adaptation Model. *Nursing & Health Sciences*, *15*(1), 31–38. https://doi.org/10.1111/j.1442-2018.2012.00715.x

487. Orlandi, G., Sofi, F., Moscarelli, L., Cirami, L., Mancini, S., & Stefani, L. (2020). Exercise Prescription in Renal Transplant Recipients: From Sports Medicine Toward Multidisciplinary Aspects: A Pilot Study. *Journal of Functional Morphology & Kinesiology*, *5*(1), 30-undefined. https://doi.org/10.3390/jfmk5010010
488. Orr, A., Orr, D., Willis, S., Holmes, M., & Britton, P. (2007). Patient perceptions of factors influencing adherence to medication following kidney transplant. *Psychology, Health & Medicine*, *12*(4), 509–517. https://doi.org/10.1080/13548500701294556
489. Orr, A., Willis, S., Holmes, M., Britton, P., & Orr, D. (2007). Living with a kidney transplant: A qualitative investigation of quality of life. *Journal of Health Psychology*, *12*(4), 653–662. https://doi.org/10.1177/1359105307078172
490. Othman, N., Gheith, O., Al-Otaibi, T., Abdou, H., Halim, M. A., Mahmoud, T., Nair, P., Yagan, J., Maher, A., Dahab, M., & Yahya, A. (2019). Role of Diabetes Education Program in Controlling Posttransplant Diabetes in a Recent Renal Transplant Bodybuilder: Case Report and Review of the Literature. *Experimental and Clinical Transplantation*, *17*(Suppl 1), 169–171. https://doi.org/10.6002/ect.MESOT2018.P46
491. Othman, N., Gheith, O., Al-Otaibi, T., Mahmoud, T., Al-Refaei, F., Mahmoud, F., Abduo, H., Nampoory, N., Halim, M. A., & Najeb, A. (2019). Assessment of Diabetes Knowledge Among Renal Transplant Recipients With Posttransplant Diabetes Mellitus: Kuwait Experience. *Experimental and Clinical Transplantation*, *17*(Suppl 1), 277–285. https://doi.org/10.6002/ect.MESOT2018.P126
    - Othman, N. (2017). „Outcome of Diabetes Education Among Renal Transplant Recipients With New Onset Diabetes After Transplantation (NODAT)“ (NODAT-ES) (Clinical trial registration NCT04030013). clinicaltrials.gov. https://clinicaltrials.gov/ct2/show/NCT04030013
492. Otwell, J. A., & Leidigh, J. C. (1988). Teaching renal transplant patients with steroid-induced diabetes mellitus. *ANNA Journal*, *15*(5), 295–304.
493. Overman, J. A., Cox, D. L., Buchl, L. L., Campion, J. K., Raihle, P. C., & Sloan, T. R. (1989). Role of the nurse in the multidisciplinary team approach to care of liver transplant patients. *Mayo Clinic Proceedings*, *64*(6), 690–698. https://doi.org/10.1016/s0025-6196(12)65349-x
494. Painter, P. (1999). Exercise after renal transplantation. *Advances in Renal Replacement Therapy*, *6*(2), 159–164. https://doi.org/10.1016/S1073-4449(99)70034-8
495. Palardy, L. G., & March, A. L. (2011). The circle of caring model: Medication adherence in cardiac transplant patients. *Nursing Science Quarterly*, *24*(2), 120–125. https://dx.doi.org/10.1177/0894318411399463
496. Pangarakis, S. J., Harrington, K., Lindquist, R., Peden-McAlpine, C., & Finkelstein, S. (2008). Electronic feedback messages for home spirometry lung transplant recipients. *Heart & Lung*, *37*(4), 299–307. https://doi.org/10.1016/j.hrtlng.2007.07.001
497. Panyi, N., & Erim, Y. (2017). [Manualized psychotherapy for the optimization of immunosuppressant adherence following kidney transplantation: Results of a feasibility study]. *Zeitschrift für Psychosomatische Medizin und Psychotherapie*, *63*(2), 189–201. https://doi.org/10.13109/zptm.2017.63.2.189
498. Patel, P. H., Bibee, K., Lim, G., Malik, S. M., Wu, C., & Pugliano-Mauro, M. (2017). Evaluating Retention of Skin Cancer Education in Kidney Transplant Recipients Reveals a Window of Opportunity for Re-education. *Transplantation Proceedings*, *49*(6), 1318–1324. https://doi.org/10.1016/j.transproceed.2017.03.079
499. Patel, S. S., Siddiqui, M. B., Chadrakumaran, A., Faridnia, M., Lin, F.-P., Hernandez Roman, J., Carbone, S., Laurenzo, J., Clinton, J., Kirkman, D., Wolver, S., Celi, F., Bhati, C., & Siddiqui, M. S. (2020). Office-Based Weight Loss Counseling Is Ineffective in Liver Transplant Recipients. *Digestive Diseases & Sciences*, *65*(2), 639–646. https://doi.org/10.1007/s10620-019-05800-6
500. Paterson, T. S. E., O'Rourke, N., Shapiro, R. J., & Loken Thornton, W. (2018). Medication adherence in renal transplant recipients: A latent variable model of psychosocial and neurocognitive predictors. *PloS One*, *13*(9), e0204219. https://doi.org/10.1371/journal.pone.0204219

501. Patzer, R. E., Serper, M., Reese, P. P., Przytula, K., Koval, R., Ladner, D. P., Levitsky, J. M., Abecassis, M. M., & Wolf, M. S. (2016). Medication understanding, non-adherence, and clinical outcomes among adult kidney transplant recipients. *Clinical Transplantation*, *30*(10), 1294–1305. https://doi.org/10.1111/ctr.12821
502. Paul, R., Hynes, M., & Lew, S. Q. (2019). Nutrition in kidney transplant recipients to prevent downstream health issues. *Journal of Kidney Care*, *4*(5), 252–260. https://doi.org/10.12968/jokc.2019.4.5.252
503. Payne, G. M., & Harrison, B. (1984). Reducing stress in renal patients and their families: A nurse-managed patient support group... Transplant support group. *Journal of Nephrology Nursing*, *1*(3), 138–140.
504. Pehlivan, S., Vatansever, N., Arslan, I., Yildiz, A., & Ersoy, A. (2020). Level of Daily Life Activities and Learning Needs in Renal Transplant Patients. *Experimental and Clinical Transplantation*, *18*(4), 498–504. https://doi.org/10.6002/ect.2018.0151
505. Pen-Chen, K., Mei Chang, Y., Ming-Kuen, L., & Hsueh Erh, L. (2017). Renal Transplant Recipients: The Factors Related to Immunosuppressive Medication Adherence Based on the Health Belief Model. *Journal of Nursing Research*, *25*(5), 392–397. https://doi.org/10.1097/jnr.0000000000000181
506. Perez, C., Busch, A., Oyama, K., & Jackson, N. (2007). Collaboration to improve education after liver transplant: The experience at Portland Veterans Affairs Medical Center. *Progress in Transplantation*, *17*(4), 302–309.
507. Perez, R. (1993). Managing nutrition problems in transplant patients. *Nutrition in Clinical Practice*, *8*(1), 28–32. https://doi.org/10.1177/011542659300800128
508. Pérez-San-Gregorio, M. Á., Martín-Rodríguez, A., Borda-Mas, M., Avargues-Navarro, M. L., Pérez-Bernal, J., & Gómez-Bravo, Á. (2017). Coping strategies in liver transplant recipients and caregivers according to patient posttraumatic growth. *Frontiers in Psychology*, *8*(Article 18). https://doi.org/10.3389/fpsyg.2017.00018
509. Peters, T. G., Spinola, K. N., West, J. C., Aeder, M. I., Danovitch, G. M., Klintmalm, G. B., Gorman, K. J., Gordon, J. A., Kincaid, C. H., & First, M. R. (2004). Differences in patient and transplant professional perceptions of immunosuppression-induced cosmetic side effects. *Transplantation*, *78*(4), 537–543. https://doi.org/10.1097/01.tp.0000139253.30998.57
510. Petitt, E. K., & Wingo, N. P. (2018). Sun protective behavior use in organ transplantrecipients current practices in a high-risk population. *Journal of the Dermatology Nurses' Association*, *10*(2), 97–112. http://dx.doi.org/10.1097/JDN.0000000000000386
511. Petre, O. A., Craciun, I. C., & Baban, A. (2021). The experiences of living with a transplanted kidney from a deceased donor. *Journal of Renal Care*, *47*(1), 58–67. https://dx.doi.org/10.1111/jorc.12349
512. Peyrovi, H., Raiesdana, N., & Mehrdad, N. (2014). Living with a heart transplant: A phenomenological study. *Progress in Transplantation*, *24*(3), 234–241. https://doi.org/10.7182/pit2014966
513. Pfeifer, P. M., Ruschel, P. P., & Bordignon, S. (2013). Coping strategies after heart transplantation: Psychological implications. *Revista Brasileira de Cirurgia Cardiovascular: Orgao Oficial da Sociedade Brasileira de Cirurgia Cardiovascular*, *28*(1), 61–68. https://doi.org/10.5935/1678-9741.20130010
514. Pinter, J., Hanson, C. S., Chapman, J. R., Wong, G., Craig, J. C., Schell, J. O., & Tong, A. (2017). Perspectives of Older Kidney Transplant Recipients on Kidney Transplantation. *Clinical Journal of The American Society of Nephrology: CJASN*, *12*(3), 443–453. https://doi.org/10.2215/CJN.05890616
515. Pinter, J., Hanson, C. S., Craig, J. C., Chapman, J. R., Budde, K., Halleck, F., & Tong, A. (2016). 'I feel stronger and younger all the time'-perspectives of elderly kidney transplant recipients: Thematic synthesis of qualitative research. *Nephrology Dialysis Transplantation*, *31*(9), 1531–1540. https://doi.org/10.1093/ndt/gfv463
516. Pisanti, R., Lombardo, C., Luszczynska, A., Poli, L., Bennardi, L., Giordanengo, L., Berloco, P. B., & Violani, C. (2017). Appraisal of transplant-related stressors, coping

strategies, and psychosocial adjustment following kidney transplantation. *Stress & Health*, *33*(4), 437–447. https://doi.org/10.1002/smi.2727
517. Pisanti, R., Poli, L., Lombardo, C., Bennardi, L., Giordanengo, L., Berloco, P. B., & Violani, C. (2014). The role of transplant-related stressors and social support in the development of anxiety among renal transplant recipients: The direct and buffering effects. *Psychology, Health & Medicine*, *19*(6), 650–655. https://dx.doi.org/10.1080/13548506.2014.882514
518. Plank, L. D., & Russell, K. (2015). Nutrition in liver transplantation: Too little or too much? *Current Opinion in Clinical Nutrition & Metabolic Care*, *18*(5), 501–507. https://doi.org/10.1097/MCO.0000000000000205
519. Pomey, M.-P., Gallego, F. B., Affdal, A., & Fortin, M.-C. (2021). Peer Mentoring as an Avenue to Explore in Kidney Transplantation: Kidney Transplant Recipients' Perspectives on Peer Mentoring. *Transplantation Direct*, *7*(3), e672. https://doi.org/10.1097/TXD.0000000000001130
520. Ponticelli, C., & Graziani, G. (2012). Education and counseling of renal transplant recipients. *Journal of Nephrology*, *25*(6), 879–889. https://doi.org/10.5301/jn.5000227
521. Poole, J., Ward, J., DeLuca, E., Shildrick, M., Abbey, S., Mauthner, O., & Ross, H. (2016). Grief and loss for patients before and after heart transplant. *Heart & Lung*, *45*(3), 193–198. https://doi.org/10.1016/j.hrtlng.2016.01.006
522. Prendergast, M. B., & Gaston, R. S. (2010). Optimizing medication adherence: An ongoing opportunity to improve outcomes after kidney transplantation. *Clinical Journal of The American Society of Nephrology: CJASN*, *5*(7), 1305–1311. https://doi.org/10.2215/CJN.07241009
523. Prieto, L. R., Miller, D. S., Gayowski, T., & Marino, I. R. (1997). Multicultural issues in organ transplantation: The influence of patients' cultural perspectives on compliance with treatment. *Clinical Transplantation*, *11*(6), 529–535.
524. Rainer, J. P., Thompson, C. H., & Lambros, H. (2010). Psychological and psychosocial aspects of the solid organ transplant experience—A practice review. *Psychotherapy: Theory, Research, Practice, Training*, *47*(3), 403–412. https://dx.doi.org/10.1037/a0021167
525. Raiz, L., Davies, E. A., & Ferguson, R. M. (2002). Sexual functioning following renal transplantation. *Health & Social Work*, *28*(4), 264–272. https://doi.org/10.1093/hsw/28.4.264
526. Raiz, L. R., Kilty, K. M., Henry, M. L., & Ferguson, R. M. (1999). Medication compliance following renal transplantation. *Transplantation*, *68*(1), 51–55. https://doi.org/10.1097/00007890-199907150-00010
527. Ranahan, M., Von Visger, J., & Kayler, L. K. (2020). Describing barriers and facilitators for medication adherence and self-management among kidney transplant recipients using the information-motivation-behavioral skills model. *Clinical Transplantation*, *34*(6), e13862. https://doi.org/10.1111/ctr.13862
528. Randolph, S., & Scholz, K. (1999). Self-care guidelines: Finding a common ground. *Journal of Transplant Coordination*, *9*(3), 156–160. https://doi.org/10.7182/prtr.1.9.3.13xk103386xl3242
529. Reali, L., Zuliani, E., Gabutti, L., Schönholzer, C., & Marone, C. (2009). Poor oral hygiene enhances gingival overgrowth caused by calcineurin inhibitors. *Journal of Clinical Pharmacy & Therapeutics*, *34*(3), 255–260. https://doi.org/10.1111/j.1365-2710.2008.01000.x
530. Rebafka, A. (2016). Medication Adherence After Renal Transplantation-a Review of the Literature. *Journal of Renal Care*, *42*(4), 239–256. https://doi.org/10.1111/jorc.12181
531. Rebafka, A., Bennett, C., Jones, J., Carrier, J., Kugler, C., & Edwards, D. (2018). Lung transplant recipients' experiences of and attitudes towards self-management: A qualitative systematic review protocol. *JBI Database of Systematic Reviews and Implementation Reports*, *16*(4), 831–837. https://doi.org/10.11124/JBISRIR-2017-003524

532. Reber, S., Morawa, E., Stößel, L., Jank, S., Vitinius, F., Eckardt, K.-U., & Erim, Y. (2016). [Prevalence and modifiable determinants of non-adherence in adult kidney transplant recipients in a German sample]. *Zeitschrift für Psychosomatische Medizin und Psychotherapie*, *62*(3), 270–283. https://doi.org/10.13109/zptm.2016.62.3.270
533. Redman, B. K. (2009). Patient Adherence or Patient Self-Management in Transplantation: An Ethical Analysis. *Progress in Transplantation*, *19*(1), 90–94. https://doi.org/10.1177/152692480901900113
534. Reynolds, L. R., & Tannock, L. R. (2008). Management of new-onset diabetes mellitus after transplantation. *Postgraduate Medicine*, *120*(2), 60–66. https://doi.org/10.3810/pgm.2008.07.1792
535. Rezende Anastácio, L., García Ferreira, L., Costa Liboredo, J., de Sena Ribeiro, H., Soares Lima, A., García Vilela, E., & Correia, M. I. (2012). Overweight, obesity and weight gain up to three years after liver transplantation. *Nutricion Hospitalaria*, *27*(4), 1351–1356. https://doi.org/10.3305/nh.2012.27.4.5768
536. Ribeiro, M. N. S., Santo, F., Simoes, B. D. S., Diniz, C. X., Bezerra, H. C. A., & Santos, L. D. (2021). Feelings, experiences and expectations of kidney transplant individuals and challenges for the nurse. *Revista Brasileira de Enfermagem*, *74*(1), e20200392-undefined. https://doi.org/10.1590/0034-7167-2020-0392
537. Robertson, G. (1999). Individuals' perception of their quality of life following a liver transplant: An exploratory study. *Journal of Advanced Nursing*, *30*(2), 497–505. https://doi.org/10.1046/j.1365-2648.1999.01105.x
538. Robinson, J., Friedewald, J., Gordon, E., Robinson, J. K., & Gordon, E. J. (2016). Perceptions of Risk of Developing Skin Cancer for Diverse Audiences: Enhancing Relevance of Sun Protection to Reduce the Risk. *Journal of Cancer Education*, *31*(1), 153–157. https://doi.org/10.1007/s13187-015-0885-1
539. Robinson, J. K., Alam, M., Ashourian, N., Khan, M., Kundu, R., Laumann, A. E., Schlosser, B. J., Yoo, S., & Gordon, E. J. (2010). Skin cancer prevention education for kidney transplant recipients: A systematic evaluation of Internet sites. *Progress in Transplantation*, *20*(4), 344–349. https://doi.org/10.7182/prtr.20.4.9877500752888660
540. Robinson, J. K., Friedewald, J. J., Desai, A., & Gordon, E. J. (2015). Response Across the Health-Literacy Spectrum of Kidney Transplant Recipients to a Sun-Protection Education Program Delivered on Tablet Computers: Randomized Controlled Trial. *JMIR cancer*, *1*(2), e8-undefined. https://dx.doi.org/10.2196/cancer.4787
541. Robinson, J. K., Guevara, Y., Gaber, R., Clayman, M. L., Kwasny, M. J., Friedewald, J. J., & Gordon, E. J. (2014). Efficacy of a sun protection workbook for kidney transplant recipients: A randomized controlled trial of a culturally sensitive educational intervention. *American Journal of Transplantation*, *14*(12), 2821–2829. https://doi.org/10.1111/ajt.12932
542. Robinson, J. K., & Rigel, D. S. (2004). Sun protection attitudes and behaviors of solid-organ transplant recipients. *Dermatologic Surgery*, *30*(4 Pt 2), 610–615. https://doi.org/10.1111/j.1524-4725.2004.30145.x
543. Robinson, J. K., Turrisi, R., Mallett, K. A., Stapleton, J., Boone, S. L., Kim, N., Riyat, N. V., & Gordon, E. J. (2011). Efficacy of an Educational Intervention With Kidney Transplant Recipients to Promote Skin Self-examination for Squamous Cell Carcinoma Detection. *Archives of Dermatology*, *147*(6), 689–695. https://doi.org/10.1001/archdermatol.2011.10
    - Robinson, J. (2010). *Warning Signs of Squamous Cell Carcinoma and Prevention of SCC by at Risk Organ Transplant Recipients* (Clinical trial registration NCT01127737). clinicaltrials.gov. https://clinicaltrials.gov/ct2/show/NCT01127737
544. Rodrigue, J. R., Nelson, D. R., Hanto, D. W., Reed, A. I., & Curry, M. P. (2013). Patient-reported immunosuppression nonadherence 6 to 24 months after liver transplant: Association with pretransplant psychosocial factors and perceptions of health status change. *Progress in Transplantation*, *23*(4), 319–328. https://doi.org/10.7182/pit2013501

545. Rodrigue, J. R., Nelson, D. R., Reed, A. I., Hanto, D. W., & Curry, M. (2010). Fatigue and sleep quality before and after liver transplantation. *Progress in Transplantation*, *20*(3), 221–233. https://doi.org/10.7182/prtr.20.3.x82q1832184j4733
546. Rodrigue, J. R., Reed, A. I., Nelson, D. R., Jamieson, I., Kaplan, B., & Howard, R. J. (2007). The financial burden of transplantation: A single-center survey of liver and kidney transplant recipients. *Transplantation*, *84*(3), 295–300. https://doi.org/10.1097/01.tp.0000269797.41202.79
547. Rosaasen, N., Mainra, R., Shoker, A., Wilson, J., Blackburn, D., & Mansell, H. (2017). Education Before Kidney Transplantation. *Progress in Transplantation*, *27*(1), 58–64. https://doi.org/10.1177/1526924816685862
548. Rosaasen, N., Taylor, J., Blackburn, D., Mainra, R., Shoker, A., & Mansell, H. (2017). Development and Validation of the Kidney Transplant Understanding Tool (K-TUT). *Transplantation Direct*, *3*(3), e132-undefined. https://doi.org/10.1097/TXD.0000000000000647
549. Rose, R. F., Moniem, K., Seukeran, D. C., Stables, G. I., & Newstead, C. G. (2005). Compliance of renal transplant recipients with advice about sun protection measures: Completing the audit cycle. *Transplantation Proceedings*, *37*(10), 4320–4322. https://doi.org/10.1016/j.transproceed.2005.10.010
550. Rosenberger, E. M., DeVito Dabbs, A. J., DiMartini, A. F., Landsittel, D. P., Pilewski, J. M., & Dew, M. A. (2017). Long-Term Follow-up of a Randomized Controlled Trial Evaluating a Mobile Health Intervention for Self-Management in Lung Transplant Recipients. *American Journal of Transplantation*, *17*(5), 1286–1293. https://doi.org/10.1111/ajt.14062
551. Rosenberger, J., Geckova, A. M., van Dijk, J. P., Nagyova, I., Roland, R., van den Heuvel, W. J. A., & Groothoff, J. W. (2005). Prevalence and characteristics of noncompliant behaviour and its risk factors in kidney transplant recipients. *Transplant International*, *18*(9), 1072–1078. https://doi.org/10.1111/j.1432-2277.2005.00183.x
552. Roso, C. C., & Henriqueta Luce Kruse, M. (2017). Life on Facebook: Self-care in renal transplantation patients. *Revista Gaúcha de Enfermagem*, *38*(2), 1–8. https://doi.org/10.1590/1983-1447.2017.02.67430
553. Rozenberg, D., Santa Mina, D., Nourouzpour, S., Camacho Perez, E., Stewart, B. L., Wickerson, L., Tsien, C., Selzner, N., Shore, J., Aversa, M., Woo, M., Holdsworth, S., Prevost, K., Park, J., Azhie, A., Huszti, E., McLeod, E., Dales, S., & Bhat, M. (2022). Feasibility of a Home-Based Exercise Program for Managing Posttransplant Metabolic Syndrome in Lung and Liver Transplant Recipients: Protocol for a Pilot Randomized Controlled Trial. *JMIR Research Protocols*, *11*(3), e35700. https://doi.org/10.2196/35700
    - University Health Network, Toronto. (2021). Feasibility of a Home-Based Exercise Program in Lung and Liver Transplant Recipients for Management of Post-Transplant Metabolic Syndrome: A Pilot Randomized Controlled Trial (Clinical trial registration NCT04965142). clinicaltrials.gov. https://clinicaltrials.gov/ct2/show/NCT04965142
554. Rudman, L. A., Gonzales, M. H., & Borgida, E. (1999). Mishandling the gift of life: Noncompliance in renal transplant patients. *Journal of Applied Social Psychology*, *29*(4), 834–851. https://doi.org/10.1111/j.1559-1816.1999.tb02028.x
555. Ruppar, T. M., & Russell, C. L. (2009). Medication adherence in successful kidney transplant recipients. *Progress in Transplantation*, *19*(2), 167–172. https://doi.org/10.1177/152692480901900211
556. Russell, C., Conn, V., Ashbaugh, C., Madsen, R., Wakefield, M., Webb, A., Coffey, D., & Peace, L. (2011). Taking immunosuppressive medications effectively (TIMELink): A pilot randomized controlled trial in adult kidney transplant recipients. *Clinical Transplantation*, *25*(6), 864–870. https://doi.org/10.1111/j.1399-0012.2010.01358.x
557. Russell, C. L. (2006). Culturally responsive interventions to enhance immunosuppressive medication adherence in older African American kidney transplant

recipients. *Progress in Transplantation*, *16*(3), 187–195; quiz 196. https://doi.org/10.1177/152692480601600302
558. Russell, C. L. (2010). A clinical nurse specialist-led intervention to enhance medication adherence using the plan-do-check-act cycle for continuous self-improvement. *Clinical Nurse Specialist*, *24*(2), 69–75. https://doi.org/10.1097/NUR.0b013e3181cf554d
559. Russell, C. L., Ashbaugh, C., Peace, L., Cetingok, M., Hamburger, K. Q., Owens, S., Coffey, D., Webb, A. W., Hathaway, D., Winsett, R. P., Madsen, R., & Wakefield, M. R. (2013). Time-in-a-bottle (TIAB): A longitudinal, correlational study of patterns, potential predictors, and outcomes of immunosuppressive medication adherence in adult kidney transplant recipients. *Clinical Transplantation*, *27*(5), E580-590. https://doi.org/10.1111/ctr.12203
560. Russell, C. L., Conn, V. S., Ashbaugh, C., Madsen, R., Hayes, K., & Ross Jr, G. (2007). Intrasubject medication adherence patterns. *Clinical Nursing Research*, *16*(2), 153–163. https://doi.org/10.1177/1054773806296429
561. Russell, C. L., Hathaway, D., Remy, L. M., Aholt, D., Clark, D., Miller, C., Ashbaugh, C., Wakefield, M., Ye, S., Staggs, V. S., Ellis, R. J., & Goggin, K. (2019). Improving medication adherence and outcomes in adult kidney transplant patients using a personal systems approach: SystemCHANGE^{TM} results of the MAGIC randomized clinical trial. *American Journal of Transplantation*, *20*(1), 125–136. https://doi.org/10.1111/ajt.15528
    - Russell, C. (2015). *SystemCHANGE: An Intervention for Medication Adherence in Transplant Recipients* (Clinical trial registration NCT02416479). clinicaltrials.gov. https://clinicaltrials.gov/ct2/show/NCT02416479
    - Russell, C. L., Andrews, A. M., O'Brien, T., Miller, C., Aholt, D., Hathaway, D., Remy, L. M., Clark, D., Goggin, K., Wessol, J. L., & Hardinger, K. (2018). Improvement of Immunosuppressive Medication Adherence Using a SystemCHANGE Intervention: Case Study of an Older Adult Kidney Transplant Recipient. *Nephrology Nursing Journal*, *45*(2), 171-223.
    - Russell, C. L., Moore, S., Hathaway, D., An-Lin, C., Guoqing, C., Goggin, K., Cheng, A.-L., & Chen, G. (2016). MAGIC Study: Aims, Design and Methods using SystemCHANGE^{TM} to Improve Immunosuppressive Medication Adherence in Adult Kidney Transplant Recipients. *BMC Nephrology*, *17*(84), 1–12. https://doi.org/10.1186/s12882-016-0285-8
562. Russell, C. L., Kilburn, E., Conn, V. S., Libbus, M. K., & Ashbaugh, C. (2003). Medication-taking beliefs of adult renal transplant recipients. *Clinical Nurse Specialist*, *17*(4), 200–208; quiz 209. https://doi.org/10.1097/00002800-200307000-00018
563. Ruzyczka, E. W., Milaniak, I., Przybylowski, P., Wierzbicki, K., Siwinska, J., Hubner, F. K., & Sadowski, J. (2011). Depression and quality of life in terms of personal resources in heart transplant recipients. *Transplantation Proceedings*, *43*(8), 3076–3081. https://doi.org/10.1016/j.transproceed.2011.07.012
564. Saadatpanah, S., Zare, N. V., Malekzadeh, J., Sadeghi, T., & Khorashadizadeh, F. (2018). Relationship between coping and spiritual health in renal transplant recipients. *Evidence Based Care Journal*. https://doi.org/10.22038/ebcj.2017.26137.1603
565. Sabati, N., Snyder, M., Edin-Stibbe, C., Lindgren, B., & Finkelstein, S. (2001). Facilitators and barriers to adherence with home monitoring using electronic spirometry. *AACN Clinical Issues*, *12*(2), 178–185. https://doi.org/10.1097/00044067-200105000-00002
566. Sabbatini, M., Ferreri, L., Pisani, A., Capuano, I., Morgillo, M., Memoli, A., Riccio, E., & Guida, B. (2019). Nutritional management in renal transplant recipients: A transplant team opportunity to improve graft survival. *Nutrition Metabolism and Cardiovascular Diseases*, *29*(4), 319–324. https://doi.org/10.1016/j.numecd.2019.01.002
567. Salehi, Z., Taghadosi, M., & Afazel, M. R. (2015). An investigation on the effect of continuous care model on depression, anxiety and stress among renal transplant patients. *Biomedical and Pharmacology Journal*, *8*(March Special Edition), 131-137. https://doi.org/10.13005/bpj/569

568. Salyer, J., Flattery, M., Joyner, P., Friend, J., & Elswick, R. K. (2007). Community-based weight management in long-term heart transplant recipients: A pilot study. *Progress in Transplantation*, *17*(4), 315–323. https://doi.org/10.1177/152692480701700410
569. Salyer, J., Flattery, M. P., Joyner, P. L., & Elswick, R. K. (2003). Lifestyle and quality of life in long-term cardiac transplant recipients. *Journal of Heart & Lung Transplantation*, *22*(3), 309–321. https://doi.org/10.1016/s1053-2498(02)00552-1
570. Salyer, J., Sneed, G., & Corley, M. C. (2001). Lifestyle and health status in long-term cardiac transplant recipients. *Heart & Lung*, *30*(6), 445–457. https://doi.org/10.1067/mhl.2001.119351
571. Sanchez, Z. V., Cashion, A. K., Cowan, P. A., Jacob, S. R., Wicks, M. N., & Velasquez-Mieyer, P. (2007). Perceived barriers and facilitators to physical activity in kidney transplant recipients. *Progress in Transplantation*, *17*(4), 324–331. https://doi.org/10.1177/152692480701700411
572. Sanders-Pinheiro, H., Colugnati, F. A. B., Marsicano, E. O., De Geest, S., & Medina, J. O. P. (2018). Prevalence and correlates of non-adherence to immunosuppressants and to health behaviours in patients after kidney transplantation in Brazil—The ADHERE BRAZIL multicentre study: A cross-sectional study protocol. *BMC Nephrology*, *19*(1), 41-undefined. https://doi.org/10.1186/s12882-018-0840-6
573. Sargent, S., & Wainwright, S. P. (2007). A qualitative study exploring patients perceived quality of life following an emergency liver transplant for acute liver failure. *Intensive & Critical Care Nursing*, *23*(5), 272–280. https://doi.org/10.1016/j.iccn.2007.03.005
574. Savadori, L., Lotto, L., & Rumiati, R. (2002). Life after heart transplantation: Risk perception in patients and their medical team. *European Psychologist*, *7*(4), 285–294. https://doi.org/10.1027//1016-9040.7.4.285
575. Savitch, S., Gilmore, R., & Dowler, D. L. (2003). An Investigation of the Psychological and Psychosocial Challenges Faced by Post-Transplant Organ Recipients. *Journal of Applied Rehabilitation Counseling*, *34*(3), 3–9. https://doi.org/10.1891/0047-2220.34.3.3
576. Saxena, D. (2020). *Can behavior therapy to enhance coping mechanism benefit Living Donor Liver Transplant recipients? - Comparison between two groups with and without behavior therapy* (Clinical trial registration CTRI/2020/09/028139). Clinical Trials Registry - India (CTRI). ctri.nic.in/Clinicaltrials/pmaindet2.php?trialid=47649&EncHid=&userName=Living Donor Liver Transplant
577. Schaevers, V., Schoonis, A., Frickx, G., Verleden, G., Jans, C., Rosseel, C., Meelberghs, M., Reinquin, I., Dobbels, F., Interdisciplinary Lung Transplantation, T., & the Information Technology, E. (2012). Implementing a standardized, evidence-based education program using the patient's electronic file for lung transplant recipients. *Progress in Transplantation*, *22*(3), 264–270. https://doi.org/10.7182/pit2012366
578. Schäfer-Keller, P., Dickenmann, M., Berry, D. L., Steiger, J., Bock, A., & De Geest, S. (2009). Computerized patient education in kidney transplantation: Testing the content validity and usability of the Organ Transplant Information System (OTIS). *Patient Education and Counseling*, *74*(1), 110–117. https://doi.org/10.1016/j.pec.2008.03.027
579. Schäfer-Keller, P., Lyon, S., Van-Gelder, F., & De Geest, S. (2006). A practical approach to promoting adherence to immunosuppressive medication after renal transplantation. *Current Opinion in Nephrology & Hypertension*, *15*, S1-6. https://doi.org/10.1097/01.mnh.0000238111.31598.a2
580. Scheel, J. F., Schieber, K., Reber, S., Stoessel, L., Waldmann, E., Jank, S., Eckardt, K.-U., Grundmann, F., Vitinius, F., de Zwaan, M., Bertram, A., & Erim, Y. (2018). Psychosocial variables associated with immunosuppressive medication non-adherence after renal transplantation. *Frontiers in Psychiatry*, *9*. https://doi.org/10.3389/fpsyt.2018.00023

581. Schipper, K., Abma, T. A., Koops, C., Bakker, I., Sanderman, R., & Schroevers, M. J. (2014). Sweet and sour after renal transplantation: A qualitative study about the positive and negative consequences of renal transplantation. *British Journal of Health Psychology*, *19*(3), 580–591. https://doi.org/10.1111/bjhp.12057
582. Schmalz, G., Kauffels, A., Kollmar, O., Slotta, J. E., Vasko, R., Müller, G. A., Haak, R., & Ziebolz, D. (2016). Oral behavior, dental, periodontal and microbiological findings in patients undergoing hemodialysis and after kidney transplantation. *BMC Oral Health*, *16*, 1–9. https://doi.org/10.1186/s12903-016-0274-0
583. Schmalz, G., Wendorff, H., Marcinkowski, A., Weinreich, G., Teschler, H., Haak, R., Sommerwerck, U., & Ziebolz, D. (2018). Oral health related quality of life depending on oral health and specific factors in patients after lung transplantation. *The Clinical Respiratory Journal*, *12*(2), 731–737. https://doi.org/10.1111/crj.12625
584. Schmid, A., Hils, S., Kramer-Zucker, A., Bogatyreva, L., Hauschke, D., De Geest, S., & Pisarski, P. (2017). Telemedically Supported Case Management of Living-Donor Renal Transplant Recipients to Optimize Routine Evidence-Based Aftercare: A Single-Center Randomized Controlled Trial. *American Journal of Transplantation*, *17*(6), 1594–1605. https://doi.org/10.1111/ajt.14138
    - Universitätsklinikum Freiburg. (2015). *Telemedizinisch unterstützte Nachsorge nierenlebend-transplantierter Patienten* (Clinical trial registration DRKS00007634). German Clinical Trials Register [DRKS]. https://www.drks.de/drks_web/navigate.do?navigationId=trial.HTML&TRIAL_ID=DRKS00007634
585. Schmid-Mohler, G., & Albiez, T. (2010). [Living safely with a transplanted kidney]. *Krankenpflege - Soins Infirmiers*, *103*(11), 22–24.
586. Schmid-Mohler, G., Albiez, T., Schafer-Keller, P., Fehr, T., Biotti, B., & Spirig, R. (2011). [In-patient education after renal transplantation]. *Pflege*, *24*(5), 317–328. https://doi.org/10.1024/1012-5302/a000144
587. Schmid-Mohler, G., Fehr, T., Witschi, P., Albiez, T., Biotti, B., & Spirig, R. (2013). [Development of an evidence-based self-management programme for patients in the first year after renal transplantation with a focus on prevention of weight gain, physical exercise and drug adherence]. *Pflege*, *26*(3), 191–205. https://doi.org/10.1024/1012-5302/a000291
588. Schmid-Mohler, G., Schäfer-Keller, P., Frei, A., Fehr, T., & Spirig, R. (2014). A mixed-method study to explore patients' perspective of self-management tasks in the early phase after kidney transplant. *Progress in Transplantation*, *24*(1), 8–18. https://doi.org/10.7182/pit2014728
589. Schmid-Mohler, G., Thut, M. P., Wuthrich, R. P., Denhaerynck, K., & De Geest, S. (2010). Non-adherence to immunosuppressive medication in renal transplant recipients within the scope of the Integrative Model of Behavioral Prediction: A cross-sectional study. *Clinical Transplantation*, *24*(2), 213–222. https://doi.org/10.1111/j.1399-0012.2009.01056.x
590. Schmid-Mohler, G., Zala, P., Graf, N., Witschi, P., Mueller, T. F., Peter Wüthrich, R., Huber, L., Fehr, T., & Spirig, R. (2019). Comparison of a Behavioral Versus an Educational Weight Management Intervention After Renal Transplantation: A Randomized Controlled Trial. *Transplantation Direct*, *5*(12), e507. https://doi.org/10.1097/TXD.0000000000000936
    - University of Zurich. (2014). Effectiveness of an Advanced Practice Nurse-led Self-management Program on Prevention of Weight Gain, Physical Activity and Medication Adherence in Patients in the First Year After Renal Transplantation (Clinical trial registration NCT02282124). clinicaltrials.gov. https://clinicaltrials.gov/ct2/show/NCT02282124
591. Schneider, S., Winsett, R. P., Reed, L., & Hathaway, D. K. (2001). Use of structured self-monitoring in transplant education. *Progress in Transplantation*, *11*(2), 133–136. https://doi.org/10.7182/prtr.11.2.l87374j127713203

592. Scholz, U., Klaghofer, R., Dux, R., Roellin, M., Boehler, A., Muellhaupt, B., Noll, G., Wüthrich, R. P., & Goetzmann, L. (2012). Predicting intentions and adherence behavior in the context of organ transplantation: Gender differences of provided social support. *Journal of Psychosomatic Research*, *72*(3), 214–219. https://doi.org/10.1016/j.jpsychores.2011.10.008
593. Schonfeld, S., Denhaerynck, K., Berben, L., Dobbels, F., Russell, C. L., Crespo-Leiro, M. G., De Geest, S., & Bright Study, T. (2020). Prevalence and Correlates of Cost-Related Medication Nonadherence to Immunosuppressive Drugs After Heart Transplantation: The International Multicenter Cross-sectional Bright Study. *Journal of Cardiovascular Nursing*, *35*(6), 519–529. https://dx.doi.org/10.1097/JCN.0000000000000683
594. Schulz, K.-H., Ewers, H., Rogiers, X., & Koch, U. (2007). Bedarf und Inanspruchnahme psychosozialer Betreuung nach Lebertransplantation. *PPmP: Psychotherapie Psychosomatik Medizinische Psychologie*, *57*(5), 221–230. https://doi.org/10.1055/s-2006-951954
595. Scott, A. M., Martin, S. C., Stone, A. M., & Brashers, D. E. (2011). Managing multiple goals in supportive interactions: Using a normative theoretical approach to explain social support as uncertainty management for organ transplant patients. *Health Communication*, *26*(5), 393–403. https://doi.org/10.1080/10410236.2011.552479
596. Scott, P. J. (2011). Occupational therapy services to enable liver patients to thrive following transplantation. *Occupational Therapy In Health Care*, *25*(4), 240–256. https://doi.org/10.3109/07380577.2011.600427
597. Scott, P. J., & Brown, V. L. (2012). Resumption of Valued Activities in the First Year Post Liver Transplant. *Occupational Therapy in Health Care*, *26*(1), 48–63. https://doi.org/10.3109/07380577.2011.643856
598. Seijo-Bestilleiro, R., Seoane-Pillado, T., Pertega-Diaz, S., González-Martín, C., Valdes-Cañedo, F., Balboa-Barreiro, V., Fernandez-Rivera, C., Alonso-Hernandez, A., Cao-Vilariño, M., Gil-Guillen, V., & Garcia-Rodriguez, M. T. (2020). Randomized clinical trial to determine the effectiveness of CO-oximetry and anti-smoking brief advice in a cohort of kidney transplant patients who smoke. *International Journal of Medical Sciences*, *17*(17), 2673–2684. https://doi.org/10.7150/ijms.49401
    - Pita-Fernandez, S., Seijo-Bestilleiro, R., Pertega-Diaz, S., Alonso-Hernandez, A., Fernandez-Rivera, C., Cao-Lopez, M., Seoane-Pillado, T., Lopez-Calvino, B., Gonzalez-Martin, C., & Valdes-Canedo, F. (2016). A randomized clinical trial to determine the effectiveness of CO-oximetry and anti-smoking brief advice in a cohort of kidney transplant patients who smoke: Study protocol for a randomized controlled trial. *Trials*, *17*. https://doi.org/10.1186/s13063-016-1311-7
599. Seiler, A., Klaghofer, R., Drabe, N., Martin-Soelch, C., Hinderling-Baertschi, V., Goetzmann, L., Boehler, A., Buechi, S., & Jenewein, J. (2016). Patients' Early Post-Operative Experiences with Lung Transplantation: A Longitudinal Qualitative Study. *The Patient: Patient-Centered Outcomes Research*, *9*(6), 547–557. https://doi.org/10.1007/s40271-016-0174-z
600. Senft, Y., Kirsch, M., Denhaerynck, K., Dobbels, F., Helmy, R., Russell, C. L., Berben, L., De Geest, S., & BRIGHT study team. (2018). Practice patterns to improve pre and post-transplant medication adherence in heart transplant centres: A secondary data analysis of the international BRIGHT study. *European Journal of Cardiovascular Nursing: Journal of the Working Group on Cardiovascular Nursing of the European Society of Cardiology*, 1474515117747577. https://doi.org/10.1177/1474515117747577
601. Sengpiel, J., Fuehner, T., Kugler, C., Avsar, M., Bodmann, I., Boemke, A., Simon, A., Welte, T., & Gottlieb, J. (2010). Use of telehealth technology for home spirometry after lung transplantation: A randomized controlled trial. *Progress in Transplantation*, *20*(4), 310–317. https://doi.org/10.7182/prtr.20.4.5522u13010202761
602. Serper, M., Barankay, I., Chadha, S., Shults, J., Jones, L. S., Olthoff, K. M., & Reese, P. P. (2020). A randomized, controlled, behavioral intervention to promote walking after

abdominal organ transplantation: Results from the LIFT study. *Transplant International*, *33*(6), 632–643. https://doi.org/10.1111/tri.13570
603. Serper, M., Patzer, R. E., Reese, P. P., Przytula, K., Koval, R., Ladner, D. P., Levitsky, J., Abecassis, M. M., & Wolf, M. S. (2015). Medication misuse, nonadherence, and clinical outcomes among liver transplant recipients. *Liver Transplantation*, *21*(1), 22–28. https://doi.org/10.1002/lt.24023
604. Sezgin, Y., & Sezgin, A. (2020). Periodontal status and dental behaviour of heart transplant recipients and healthy controls. *Acta odontologica Scandinavica*, *78*(1), 20–25. https://doi.org/10.1080/00016357.2019.1683889
605. Shah, H. H. (2020). *Does physiotherapy help to improve the functional performance among transplant recipients?* (Clinical trial registration CTRI/2020/08/027245). Clinical Trials Registry - India (CTRI). http://ctri.nic.in/Clinicaltrials/pmaindet2.php?trialid=45888&EncHid=&userName=Does%20physiotherapy%20help%20to%20improve
606. Shahmoradi, L., Abtahi, H., Amini, S., & Gholamzadeh, M. (2020). Systematic review of using medical informatics in lung transplantation studies. *International Journal of Medical Informatics*, *136*(104096). https://doi.org/10.1016/j.ijmedinf.2020.104096
607. Sharif, A., Moore, R., & Baboolal, K. (2008). Influence of lifestyle modification in renal transplant recipients with postprandial hyperglycemia. *Transplantation*, *85*(3), 353–358. https://doi.org/10.1097/TP.0b013e3181605ebf
608. Sheikhalipour, Z., Zamanzadeh, V., Borimnejad, L., Valizadeh, L., Shahbazi, M., Zomorrodi, A., & Nazari, M. (2018). Recipients' Experiences after Organ Transplantation. *International Journal of Organ Transplantation Medicine*, *9*(2), 88–96.
609. Sherry, D. C., Simmons, B., Wung, S. F., & Zerwic, J. J. (2003). Noncompliance in heart transplantation: A role for the advanced practice nurse. *Progress in Cardiovascular Nursing*, *18*(3), 141–146. https://doi.org/10.1111/j.0889-7204.2003.02003.x
610. Shi, Y.-X., Liu, C.-X., Liu, F., Zhang, H.-M., Yu, M.-M., Jin, Y.-H., Shang, S.-M., & Fu, Y.-X. (2020). Efficacy of Adherence-Enhancing Interventions for Immunosuppressive Therapy in Solid Organ Transplant Recipients: A Systematic Review and Meta-Analysis Based on Randomized Controlled Trials. *Frontiers in Pharmacology*, *11*. https://doi.org/10.3389/fphar.2020.578887
611. Shih, F. J., Wang, S. S., Hsiao, S. M., Tseng, P. H., & Chu, S. S. (2008). Comparison of the Psychospiritual Needs of Chinese Heart Transplant Recipients at Pre- and Postoperative Stages. *Transplantation Proceedings*, *40*(8), 2597–2599. https://doi.org/10.1016/j.transproceed.2008.08.032
612. Siegal, B., & Greenstein, S. (1999). Compliance and noncompliance in kidney transplant patients: Cues for transplant coordinators. *Journal of Transplant Coordination*, *9*(2), 104–108. https://doi.org/10.7182/prtr.1.9.2.a67514546814h767
613. Siegal, B. R., & Greenstein, S. M. (1997). Postrenal transplant compliance from the perspective of African-Americans, Hispanic-Americans, and Anglo-Americans. *Advances in Renal Replacement Therapy*, *4*(1), 46–54. https://doi.org/10.1016/s1073-4449(97)70016-5
614. Silva, A. N., Moratelli, L., Tavares, P. L., Marsicano, E. D., Pinhati, R. R., Colugnati, F. A. B., Lucchetti, G., & Sanders-Pinheiro, H. (2016). Self-efficacy beliefs, locus of control, religiosity and non-adherence to immunosuppressive medications in kidney transplant patients. *Nephrology*, *21*(11), 938–943. https://doi.org/10.1111/nep.12695
615. Singer, J. P., Chen, J., Katz, P. P., Blanc, P. D., Kagawa-Singer, M., & Stewart, A. L. (2015). Defining novel health-related quality of life domains in lung transplantation: A qualitative analysis. *Quality of Life Research*, *24*(6), 1521–1533. https://doi.org/10.1007/s11136-014-0875-5
616. Siwinska, J., Lesiak-Kalukin, M., Przybylowski, P., & Sadowski, J. (2011). Health behavior of patients after heart transplantation as an indicator of patient compliance. *Transplantation Proceedings*, *43*(8), 3086–3088. https://dx.doi.org/10.1016/j.transproceed.2011.08.051

617. Skiveren, J., Mortensen, E. L., & Haedersdal, M. (2010). Sun protective behaviour in renal transplant recipients. A qualitative study based on individual interviews and the Health Belief Model. *Journal of Dermatological Treatment*, *21*(6), 331–336. https://dx.doi.org/10.3109/09546630903410166
618. Snell, L., & Randolph, S. (2004). Home care management of intestinal transplant recipients. *Progress in Transplantation*, *14*(4), 299–310. https://doi.org/10.7182/prtr.14.4.771lugl7l6215647
619. Soltannezhad, F., Farsi, Z., & Jabari Moroei, M. (2013). The effect of educating health promotion strategies on self-care self-efficacy in patients undergoing kidney transplantation: A double blind randomized trial. *Nursing & Midwifery Studies*, *2*(4), 64–70. https://doi.org/10.5812/nms.11874
    - Mirbagher, N. (2020). *The effect of educating health promotion strategies model on self-care self-efficacy* (Clinical trial registration IRCT20111210008348N48). Iranian Registry of Clinical trials (IRCT). https://www.irct.ir/trial/48928
620. Somerville, K. T., & Phillips, K. (2003). The MedPlanner: An Internet-based dynamic medication education tool. *Advances in Pharmacy*, *1*(2), 112–120.
621. Sorensen, A., Norsen, L., Chen, L., Palermo, E., & Martens, J. (2018). A Multimodal Skin Cancer Prevention Program for Heart Transplant Patients. *Progress in Transplantation*, *28*(3), 263–266. https://doi.org/10.1177/1526924818781561
622. Spillman, L. N., Melville-Claxton, A., Gatiss, G. A., Fernandez, N., & Madden, A. M. (2021). Diet and physical activity after liver transplant: A qualitative study of barriers and facilitators to following advice. *Journal of Human Nutrition and Dietetics*. https://doi.org/10.1111/jhn.12874
623. Stanfill, A., Bloodworth, R., & Cashion, A. (2012). Lessons learned: Experiences of gaining weight by kidney transplant recipients. *Progress in Transplantation*, *22*(1), 71–78. https://doi.org/10.7182/pit2012986
624. Steinberg, T. G., Diercks, M. J., & Millspaugh, J. (1996). An evaluation of the effectiveness of a videotape for discharge teaching of organ transplant recipients. *Journal of Transplant Coordination*, *6*(2), 59–63. https://doi.org/10.7182/prtr.1.6.2.e720443244wxv3p1
625. Stephenson, M. D., & Bradshaw, W. (2020). Kidney transplantation: Interventions to improve medication adherence. *Renal Society of Australasia Journal*, *16*(1), 8–12. https://doi.org/10.33235/rsaj.16.1.8-12
626. Stiavetti, E., Ghinolfi, D., Pasetti, P., Bocchi, F., & Filipponi, F. (2013). Analysis of patients' needs after liver transplantation in Tuscany: A prevalence study. *Transplantation Proceedings*, *45*(3), 1276–1278. https://doi.org/10.1016/j.transproceed.2013.02.023
627. Stiefel, P., Malehsa, D., Bara, C., Strueber, M., Haverich, A., & Kugler, C. (2013). Symptom experiences in patients after heart transplantation. *Journal of Health Psychology*, *18*(5), 680–692. https://doi.org/10.1177/1359105312454909
628. Stilley, C. S., DiMartini, A. F., de Vera, M. E., Flynn, W. B., King, J., Sereika, S., Tarter, R. E., Dew, M. A., & Rathnamala, G. (2010). Individual and environmental correlates and predictors of early adherence and outcomes after liver transplantation. *Progress in Transplantation*, *20*(1), 58–66; quiz 67. https://doi.org/10.7182/prtr.20.1.c903845857104k83
629. Stilley, C. S., Flynn, W. B., Sereika, S. M., Stimer, E. D., DiMartini, A. F., & deVera, M. E. (2012). Pathways of psychosocial factors, stress, and health outcomes after liver transplantation. *Clinical Transplantation*, *26*(2), 216–222. https://doi.org/10.1111/j.1399-0012.2011.01467.x
630. Suhling, H., Rademacher, J., Zinowsky, I., Fuge, J., Greer, M., Warnecke, G., Smits, J. M., Bertram, A., Haverich, A., Welte, T., & Gottlieb, J. (2014). Conventional vs. Tablet computer-based patient education following lung transplantation a randomized controlled trial. *PLoS ONE*, *9*(3), e90828. https://doi.org/10.1371/journal.pone.0090828
631. Sutton, T. D., & Murphy, S. P. (1989). Stressors and patterns of coping in renal transplant patients. *Nursing Research*, *38*(1), 46–49.

632. Suzuki, R., Nakamiya, Y., Watanabe, M., Ando, E., Tanichi, M., Koga, M., Kohno, K., Usui, J., Yamagata, K., Ohkohchi, N., Toda, H., Saito, T., Yoshino, A., Takahara, S., Yamauchi, K., & Yuzawa, K. (2019). Relationship Between Stress Coping Mechanisms and Depression in Kidney Transplant Recipients. *Transplantation Proceedings*, *51*(3), 761–767. https://dx.doi.org/10.1016/j.transproceed.2018.12.034
633. Svensson, M., Jardine, A., Fellstrom, B., & Holdaas, H. (2012). Prevention of cardiovascular disease after renal transplantation. *Current Opinion in Organ Transplantation*, *17*(4), 393–400. https://dx.doi.org/10.1097/MOT.0b013e3283560a3b
634. Szepietowski, J. C., Reich, A., Nowicka, D., Weglowska, J., & Szepietowski, T. (2005). Sun protection in renal transplant recipients: Urgent need for education. *Dermatology*, *211*(2), 93–97. https://doi.org/10.1159/000086435
635. Taber, D. J., Gebregziabher, M., Posadas, A., Schaffner, C., Egede, L. E., & Baliga, P. K. (2018). Pharmacist-led, technology-assisted study to improve medication safety, cardiovascular risk factor control, and racial disparities in kidney transplant recipients. *Journal of the American College of Clinical Pharmacy*, *1*(2), 81–88. https://doi.org/10.1002/jac5.1024
    - Cole, A. J., Johnson, R. W., Egede, L. E., Baliga, P. K., & Taber, D. J. (2018). Improving Medication Safety and Cardiovascular Risk Factor Control to Mitigate Disparities in African-American Kidney Transplant Recipients: Design and Methods. *Contemporary Clinical Trials Communications*, *9*, 1–6. https://doi.org/10.1016/j.conctc.2017.11.008
    - Medical University of South Carolina. (2016). *Improving Medication Safety and Cardiovascular Risk Factor Control in Kidney Transplant Recipients* (Clinical trial registration NCT02763943). clinicaltrials.gov. https://clinicaltrials.gov/ct2/show/NCT02763943
636. Tabler, J. B., & Frierson, R. L. (1990). Sexual concerns after heart transplantation. *Journal of Heart Transplantation*, *9*(4), 397–403.
637. Taher, M., Toossi, M. N., Jafarian, A., Rasti, A., & Nayeri, N. D. (2021). Patients' Experiences of Life Challenges After Liver Transplantation: A Qualitative Study. *Journal of Patient Experience*, *8*, 2374373521996956. https://doi.org/10.1177/2374373521996956
638. Taj, S. M., Baghaffar, H., Alnajjar, D. K., Almashabi, N. K., & Ismail, S. (2021). Prevalence of Non-Adherence to Immunosuppressive Medications in Kidney Transplant Recipients: Barriers and Predictors. *Annals of Transplantation*, *26*, e928356. https://doi.org/10.12659/AOT.928356
639. Takahashi, A., Hu, S. L., & Bostom, A. (2018). Physical Activity in Kidney Transplant Recipients: A Review. *American Journal of Kidney Diseases*, *72*(3), 433–443. https://doi.org/10.1053/j.ajkd.2017.12.005
640. Tang, J., James, L., Howell, M., Tong, A., & Wong, G. (2020). eHealth Interventions for Solid Organ Transplant Recipients: A Systematic Review and Meta-analysis of Randomized Controlled Trials. *Transplantation*, *104*(8), E224–E235. https://doi.org/10.1097/TP.0000000000003294
641. Tang, J., Kerklaan, J., Wong, G., Howell, M., Scholes-Robertson, N., Guha, C., Kelly, A., & Tong, A. (2021). Perspectives of solid organ transplant recipients on medicine-taking: Systematic review of qualitative studies. *American Journal of Transplantation*, *21*(10), 3369–3387. https://doi.org/10.1111/ajt.16613
642. Tavadia, S., Dawn, G., Payne, C., Ramrakha-Jones, V., Murday, A., & Holmes, S. (2006). Skin-cancer awareness in Scottish cardiac transplant recipients. *Clinical and Experimental Dermatology*, *31*(3), 354–357. https://doi.org/10.1111/j.1365-2230.2006.02098.x
643. Teng, S., Zhang, S., Zhang, W., Lin, X., Shang, Y., Peng, X., & Liu, H. (2015). Symptom Experience Associated With Immunosuppressive Medications in Chinese Kidney Transplant Recipients. *Journal of Nursing Scholarship*, *47*(5), 425–434. https://doi.org/10.1111/jnu.12157

644. Thomas, D. J. (1993). Risking infection: An issue of control for liver transplant recipients. *AACN Clinical Issues in Critical Care Nursing*, *4*(3), 471–474. https://doi.org/10.4037/15597768-1993-3002
645. Thomsen, D., & Jensen, B. (2009). Patients' experiences of everyday life after lung transplantation. *Journal of Clinical Nursing*, *18*(24), 3472–3479. https://doi.org/10.1111/j.1365-2702.2009.02828.x
646. Tielen, M., van Exel, N. J., van Buren, M. C., Maasdam, L., & Weimar, W. (2011). Attitudes towards medication non-adherence in elderly kidney transplant patients: A Q methodology study. *Nephrology Dialysis Transplantation*, *26*(5), 1723–1728. https://doi.org/10.1093/ndt/gfq642
647. Tigli, A., Ayvazoglu Soy, E. H., Aytar, A., Moray, G., & Haberal, M. (2019). Relationship Between Exercise Perception With Physical Activity Level, Body Awareness, and Illness Cognition in Renal Transplant Patients: A Pilot Study. *Experimental and Clinical Transplantation*, *17*(Suppl 1), 270–276. https://doi.org/10.6002/ect.MESOT2018.P123
648. Tohidinezhad, F., Aliakbarian, M., Abu-Hanna, A., & Eslami, S. (2019). Development and Psychometric Testing of Liver Transplant Therapeutic Adherence Questionnaire in a Triphasic Mixed-Method Study. *Progress in Transplantation*, *29*(2), 122–128. https://doi.org/10.1177/1526924819835824
649. Tong, A., Howell, M., Wong, G., Webster, A. C., Howard, K., & Craig, J. C. (2011). The perspectives of kidney transplant recipients on medicine taking: A systematic review of qualitative studies. *Nephrology Dialysis Transplantation*, *26*(1), 344–354. https://dx.doi.org/10.1093/ndt/gfq376
650. Totti, V., Campione, T., Mosconi, G., Tame, M., Tonioli, M., Gregorini, M., Scarpioni, R., Storari, A., Mignani, R., Sella, G., Bellis, L., Cardillo, M., & Sangiorgi, G. (2020). Observational Retrospective Study on Patient Lifestyle in the Pretransplantation and Post-transplantation Period in the Emilia-Romagna Region. *Transplantation Proceedings*, *52*(5), 1552–1555. https://doi.org/10.1016/j.transproceed.2020.03.015
651. Totti, V., Fernhall, B., Di Michele, R., Todeschini, P., La Manna, G., Cappuccilli, M., Angelini, M. L., De Fabritiis, M., Merni, F., Benedetti, E., Roi, G. S., Costa, A. N., & Mosconi, G. (2020). Longitudinal Analysis of Cardiovascular Risk Factors in Active and Sedentary Kidney Transplant Recipients. *Medicina*, *56*(4), 183. https://doi.org/10.3390/medicina56040183
652. Traboulsi, D., Potok, O. V., Ruzycki, S. M., Surmanowicz, P., Hardin, J., Khokhar, B., Rabi, D. M., Hazlewood, G., & Mydlarski, P. R. (2019). Skin cancer knowledge and photoprotective practices of organ transplant recipients. *Clinical Transplantation*, *33*(5), e13524. https://dx.doi.org/10.1111/ctr.13524
653. Traiger, G. L., & Bui, L. L. (1997). A self-medication administration program for transplant recipients. *Critical Care Nurse*, *17*(1), 71–79. https://doi.org/10.4037/ccn1997.17.1.71
654. Trevizan, F. B., Miyazaki, M., Silva, Y. L. W., & Roque, C. M. W. (2017). Quality of Life, Depression, Anxiety and Coping Strategies after Heart Transplantation. *Brazilian Journal of Cardiovascular Surgery*, *32*(3), 162–170. https://doi.org/10.21470/1678-9741-2017-0029
655. Trinh, N., Novice, K., Lekakh, O., Means, A., & Tung, R. (2014). Use of a brief educational video administered by a portable video device to improve skin cancer knowledge in the outpatient transplant population. *Dermatologic Surgery*, *40*(11), 1233–1239. https://dx.doi.org/10.1097/DSS.0000000000000148
656. Troosters, T. (2021). *Enhancing Physical Activity in Patients After Lung Transplantation: Effectiveness and Feasibility of a Semi-automated Tele Coaching Program* (Clinical trial registration NCT04122768). clinicaltrials.gov. https://clinicaltrials.gov/ct2/show/NCT04122768
657. Tsapepas, D. S., Salerno, D., Jandovitz, N., Hammad, S., Jordan, P., Mohan, S., Hardy, M., Kotchoubey, H., Vawdrey, D., & Fleischut, P. M. (2018). Using technology to enhance medication regimen education after solid organ transplantation. *American Journal of Health-System Pharmacy*. http://dx.doi.org/10.2146/ajhp170799

658. Tschida, S., Aslam, S., Khan, T. T., Sahli, B., Shrank, W. H., & Lal, L. S. (2013). Managing specialty medication services through a specialty pharmacy program: The case of oral renal transplant immunosuppressant medications. *Journal of Managed Care Pharmacy: JMCP*, *19*(1), 26–41. https://doi.org/10.18553/jmcp.2013.19.1.26
659. Tucker, E. L., Smith, A. R., Daskin, M. S., Schapiro, H., Cottrell, S. M., Gendron, E. S., Hill-Callahan, P., Leichtman, A. B., Merion, R. M., Gill, S. J., & Maass, K. L. (2019). Life and expectations post-kidney transplant: A qualitative analysis of patient responses. *BMC Nephrology*, *20*(1), 175-undefined. https://doi.org/10.1186/s12882-019-1368-0
660. Tunçer Vural, A., Karataş Toğral, A., Kirnap, M., Tülin Güleç, A., & Haberal, M. (2018). Skin Cancer Risk Awareness and Sun-Protective Behavior Among Solid-Organ Transplant Recipients. *Experimental and Clinical Transplantation*, *16 Suppl 1*(Suppl 1), 203–207. https://doi.org/10.6002/ect.TOND-TDTD2017.P65
661. Turner, K., Burns, T., & Tranter, S. (2018). An evaluation of the nursing care of renal transplant recipients: A qualitative study. *Renal Society of Australasia Journal*, *14*(1), 21–25.
662. Turton-Weeks, S. M., Barone, G. W., Gurley, B. J., Ketel, B. L., Lightfoot, M. L., & Abul-Ezz, S. R. (2001). St John’s wort: A hidden risk for transplant patients. *Progress in Transplantation*, *11*(2), 116–120. https://doi.org/10.7182/prtr.11.2.k761052k1r8364h5
663. Ullrich, G., Jansch, H., Schmidt, S., Struber, M., & Niedermeyer, J. (2005). Consulting the „experts“: A pilot study on perceptions of professional support among lung transplant recipients and accompanying relatives. *Progress in Transplantation*, *15*(2), 115–122. https://doi.org/10.1177/152692480501500203.
664. University of Pittsburgh. (2009). *Effect of Dietary and Life Style Modification on Post Liver Transplant Obesity* (Clinical trial registration NCT00878592). clinicaltrials.gov. https://clinicaltrials.gov/ct2/show/NCT00878592
665. Urstad, K. H., Andersen, M. H., Oyen, O., Moum, T., & Wahl, A. K. (2011). Patients’ level of knowledge measured five days after kidney transplantation. *Clinical Transplantation*, *25*(4), 646–652. https://doi.org/10.1111/j.1399-0012.2010.01355.x
666. Urstad, K. H., Oyen, O., Andersen, M. H., Moum, T., & Wahl, A. K. (2012). The effect of an educational intervention for renal recipients: A randomized controlled trial. *Clinical Transplantation*, *26*(3), E246-53. https://dx.doi.org/10.1111/j.1399-0012.2012.01666.x
    - Oslo Metropolitan University. (2014). *Effect of Patient Education on Kidney Recipients Knowledge, Coping and Quality of Life. A Randomized Controlled Trial.* (Clinical trial registration NCT01184937). clinicaltrials.gov. https://clinicaltrials.gov/ct2/show/NCT01184937
667. Urstad, K. H., Wahl, A. K., Andersen, M. H., Oyen, O., & Fagermoen, M. S. (2012). Renal recipients’ educational experiences in the early post-operative phase—A qualitative study. *Scandinavian Journal of Caring Sciences*, *26*(4), 635–642. https://dx.doi.org/10.1111/j.1471-6712.2012.00972.x
668. Urstad, K. H., Wahl, A. K., Moum, T., Engebretsen, E., & Andersen, M. H. (2021). Renal recipients’ knowledge and self-efficacy during first year after implementing an evidence based educational intervention as routine care at the transplantation clinic. *BMC Nephrology*, *22*(1), 265-undefined. https://dx.doi.org/10.1186/s12882-021-02468-x
669. Valizadeh Zare, N., Mohammadi, E., Zarea, K., Elahi, N., & Manzari, Z. (2018). The meaning of coping for kidney transplant recipients: A phenomenological study. *Journal of Research in Nursing*, *23*(7), 584–595. https://dx.doi.org/10.1177/1744987118785949
670. van Adrichem, E. J., de Zande, S. C. V., Dekker, R., Verschuuren, E. A. M., Dijkstra, P. U., & van der Schans, C. P. (2016). Perceived Barriers to and Facilitators of Physical Activity in Recipients of Solid Organ Transplantation, a Qualitative Study. *PLoS One*, *11*(9). https://doi.org/10.1371/journal.pone.0162725
671. van Adrichem, E. J., Krijnen, W. P., Dekker, R., Ranchor, A. V., Dijkstra, P. U., & van der Schans, C. P. (2017). Multidimensional structure of a questionnaire to assess barriers to and motivators of physical activity in recipients of solid organ

transplantation. *Disability & Rehabilitation*, *39*(22), 2330–2338. https://doi.org/10.1080/09638288.2016.1224274
672. van der Mei, S. F., van Son, W. J., van Sonderen, E. L., de Jong, P. E., Groothoff, J. W., & van den Heuvel, W. J. (2007). Factors determining social participation in the first year after kidney transplantation: A prospective study. *Transplantation*, *84*(6), 729–737. https://doi.org/10.1097/01.tp.0000281409.35702.53
673. van Lint, C. L., van der Boog, P. J., Wang, W., Brinkman, W. P., Rovekamp, T. J., Neerincx, M. A., Rabelink, T. J., & van Dijk, S. (2015). Patient experiences with self-monitoring renal function after renal transplantation: Results from a single-center prospective pilot study. *Patient Preference and Adherence*, *9*, 1721–1731. https://doi.org/10.2147/PPA.S92108
674. van Lint, C., Rabelink, T. J., van der Boog, P. J. M., Wenxin, W., Brinkman, W.-P., Neerincx, M. A., van Dijk, S., Rövekamp, T. J. M., & Wang, W. (2017). Self-Monitoring Kidney Function Post Transplantation: Reliability of Patient-Reported Data. *Journal of Medical Internet Research*, *19*(9), 18–18. https://doi.org/10.2196/jmir.7542
675. van Zanten, R., van Dijk, M., van Rosmalen, J., Beck, D., Zietse, R., Van Hecke, A., van Staa, A., Massey, E. K., Beck, D., van Dijk, M., Goedendorp, M., van den Hoogen, M., Ista, E., Maasdam, L., Manintveld, O., Massey, E. K., van Rosmalen, J., de Weerd, A., van Zanten, R., … On behalf of the aanZET study group. (2022). Nurse-led self-management support after organ transplantation—Protocol of a multicentre, stepped-wedge randomized controlled trial. *Trials*, *23*(1), 14. https://doi.org/10.1186/s13063-021-05896-0
    - Erasmus Medical Center, & van Zanten, R. (2020). *aanZET study—A positive approach to self-management after transplantation* (Clinical trial registration NL8469). Netherlands Trial Registry (NTR). https://trialsearch.who.int/Trial2.aspx?TrialID=NL8469
676. Vanhoof, J. M. M., Vandenberghe, B., Geerts, D., Philippaerts, P., De Mazière, P., DeVito Dabbs, A., De Geest, S., Dobbels, F., & PICASSO-Tx Consortium. (2018). Technology Experience of Solid Organ Transplant Patients and Their Overall Willingness to Use Interactive Health Technology. *Journal of Nursing Scholarship*, *50*(2), 151–162. https://doi.org/10.1111/jnu.12362
677. Vanhoof, J. M. M., Vandenberghe, B., Geerts, D., Philippaerts, P., De Mazière, P., DeVito Dabbs, A., De Geest, S., Dobbels, F., & the PICASSO-Tx consortium. (2018). Shedding light on an unknown reality in solid organ transplant patients' self-management: A contextual inquiry study. *Clinical Transplantation*, *32*(8), e13314. https://doi.org/10.1111/ctr.13314
678. Vankova, B., Mala-Ladova, K., Kubena, A. A., Maly, J., & Sulkova, S. D. (2018). Immunosuppressive therapy related adherence, beliefs and self-management in kidney transplant outpatients. *Patient Preference and Adherence*, *12*, 2605–2613. https://doi.org/10.2147/PPA.S184166
679. Vidnes, T. (2020). *Testing the Effect of a New Health Communication Intervention for Renal Transplant Recipients (KnowMAP).* (Clinical trial registration NCT04296955). clinicaltrials.gov. https://clinicaltrials.gov/ct2/show/NCT04296955
680. Villeneuve, C., Rousseau, A., Rerolle, J. P., Couzi, L., Kamar, N., Essig, M., Etienne, I., Westeel, P. F., Buchler, M., Esposito, L., Thierry, A., Marquet, P., & Monchaud, C. (2020). Adherence profiles in kidney transplant patients: Causes and consequences. *Patient Education and Counseling*, *103*(1), 189–198. https://doi.org/10.1016/j.pec.2019.08.002
681. Vintro, A. Q., Krasnoff, J. B., & Painter, P. (2002). Roles of nutrition and physical activity in musculoskeletal complications before and after liver transplantation. *AACN Clinical Issues*, *13*(2), 333–347. https://doi.org/10.1097/00044067-200205000-00016
682. Wachholz, L. F., Knihs, N. D. S., Martins, S. R., Magalhaes, A. L. P., Brehmer, L. C. F., & Martins, M. D. S. (2020). Hospital discharge of liver transplantation patient: An integrative review. *Escola Anna Nery*, *24*, e20190346. http://dx.doi.org/10.1590/2177-9465-EAN-2019-0346

683. Wainwright, S. P. (1995). The transformational experience of liver transplantation. *Journal of Advanced Nursing*, *22*(6), 1068–1076. https://doi.org/10.1111/j.1365-2648.1995.tb03107.x
684. Wainwright, S. P., & Gould, D. (1997). Non-adherence with medications in organ transplant patients: A literature review. *Journal of Advanced Nursing*, *26*(5), 968–977. https://doi.org/10.1046/j.1365-2648.1997.00451.x
685. Wang, D. W., Sills, L. L., MacDonald, S. B., Maianski, Z., & Alwayn, I. (2014). Active video gaming in patients with renal transplant: A pilot study. *Transplantation Research*, *3*, 15. https://doi.org/10.1186/2047-1440-3-15
686. Wang, H., Du, C., Liu, H., Zhang, S., Wu, S., Fu, Y., & Zhao, J. (2020). Exploration of symptom experience in kidney transplant recipients based on symptoms experience model. *Quality of Life Research*, *29*(5), 1281–1290. https://dx.doi.org/10.1007/s11136-019-02404-5
687. Wang, W., van Lint, C. L., Brinkman, W. P., Rovekamp, T. J. M., van Dijk, S., van der Boog, P., & Neerincx, M. A. (2019). Guided or factual computer support for kidney patients with different experience levels and medical health situations: Preferences and usage. *Health and Tehnology*, *9*, 329–342. https://doi.org/10.1007/s12553-019-00295-7
688. Wang, W., van Lint, C. L., Brinkman, W.-P., Rövekamp, T. J. M., van Dijk, S., van der Boog, P. J. M., & Neerincx, M. A. (2017). Renal transplant patient acceptance of a self-management support system. *BMC Medical Informatics and Decision Making*, *17*(1), 58. https://doi.org/10.1186/s12911-017-0456-y
689. Wasilewski, G. J., Milaniak, I., Janik, Ł., Sadowski, J., & Przybyłowski, P. (2014). Adherence to antihypertensive therapy among heart transplant recipients. *Kardiochirurgia I Torakochirurgia Polska = Polish Journal of Cardio-Thoracic Surgery*, *11*(3), 343–348. https://doi.org/10.5114/kitp.2014.45689
690. Wedd, J., Basu, M., Curtis, L. M., Smith, K., Lo, D. J., Serper, M., Wolf, M. S., Parker, R., & Patzer, R. E. (2019). Racial, Ethnic, and Socioeconomic Disparities in Web-Based Patient Portal Usage Among Kidney and Liver Transplant Recipients: Cross-Sectional Study. *Journal of Medical Internet Research*, *21*(4), e11864-undefined. https://dx.doi.org/10.2196/11864
691. Wei, H., Guan, Z., Zhao, J., Zhang, W., Shi, H., Wang, W., Wang, J., Xiao, X., Niu, Y., & Shi, B. (2016). Physical Symptoms and Associated Factors in Chinese Renal Transplant Recipients. *Transplantation Proceedings*, *48*(8), 2644–2649. https://dx.doi.org/10.1016/j.transproceed.2016.06.052
692. Weng, F. L., Chandwani, S., Kurtyka, K. M., Zacker, C., Chisholm-Burns, M. A., & Demissie, K. (2013). Prevalence and correlates of medication non-adherence among kidney transplant recipients more than 6 months post-transplant: A cross-sectional study. *BMC Nephrology*, *14*, 261-undefined. https://dx.doi.org/10.1186/1471-2369-14-261
693. Weng, L. C., Huang, H. L., Wang, Y. W., Lee, W. C., Chen, K. H., & Yang, T. Y. (2014). The effect of self-efficacy, depression and symptom distress on employment status and leisure activities of liver transplant recipients. *Journal of Advanced Nursing*, *70*(7), 1573–1583. https://dx.doi.org/10.1111/jan.12315
694. Weng, L. C., Yang, Y. C., Huang, H. L., Chiang, Y. J., & Tsai, Y. H. (2017). Factors that determine self-reported immunosuppressant adherence in kidney transplant recipients: A correlational study. *Journal of Advanced Nursing*, *73*(1), 228–239. https://doi.org/10.1111/jan.13106
695. Weng, L.-C., Dai, Y.-T., Huang, H.-L., & Chiang, Y.-J. (2010). Self-efficacy, self-care behaviours and quality of life of kidney transplant recipients. *Journal of Advanced Nursing*, *66*(4), 828–838. https://doi.org/10.1111/j.1365-2648.2009.05243.x
696. White, C., & Gallagher, P. (2010). Effect of patient coping preferences on quality of life following renal transplantation. *Journal of Advanced Nursing*, *66*(11), 2550–2559. https://doi.org/10.1111/j.1365-2648.2010.05410.x

697. White, M. J., Ketefian, S., Starr, A. J., & Voepel-Lewis, T. (1990). Stress, coping, and quality of life in adult kidney transplant recipients. *ANNA Journal*, *17*(6), 421–424, 431; discussion 425.
698. White-Williams, C., Grady, K. L., Fazeli, P., Myers, S., Moneyham, L., Meneses, K., & Rybarczyk, B. (2014). The partial mediation effect of satisfaction with social support and coping effectiveness on health-related quality of life and perceived stress long-term after heart transplantation. *Nursing: Research & Reviews*, *4*, 129–134. https://doi.org/10.2147/NRR.S71231
699. Wiederhold, D., Langer, G., & Landenberger, M. (2009). ["What can I still do, what may I still do?"—Ambivalent lived experiences and instruction need of patients in the early period after renal transplantation]. *Pflege*, *22*(5), 329–339. https://dx.doi.org/10.1024/1012-5302.22.5.329
    - Wiederhold, D., Langer, G., & Landenberger, M. (2011). Ambivalent Lived Experiences and Instruction Need of Patients in the Early Period After Kidney Transplantation: A Phenomenological Study. *Nephrology Nursing Journal*, *38*(5), 417–423.
700. Wietlisbach, M., Benden, C., Koutsokera, A., Jahn, K., Soccal, P. M., & Radtke, T. (2020). Perceptions towards physical activity in adult lung transplant recipients with cystic fibrosis. *PLoS One*, *15*(2). https://doi.org/10.1371/journal.pone.0229296
701. Wijbenga, N., Hoek, R. A. S., Mathot, B. J., Seghers, L., van Weezel, J. J., den Ouden, J., Wijsenbeek, M. S., Aerts, J., Hellemons, M. E., & Moor, C. C. (2020). Evaluation of a Home Monitoring Application for Follow Up after Lung Transplantation-A Pilot Study. *Journal of Personalized Medicine*, *10*(4), 240. https://doi.org/10.3390/jpm10040240
702. Wilcox, J., Waite, C., Tomlinson, L., Driscoll, J., Karim, A., Day, E., & Sharif, A. (2016). Comparing glycaemic benefits of Active Versus passive lifestyle Intervention in kidney Allograft Recipients (CAVIAR): Study protocol for a randomised controlled trial. *Trials*, *17*, 417-undefined. https://doi.org/10.1186/s13063-016-1543-6
703. Wilkinson, A., Davidson, J., Dotta, F., Home, P. D., Keown, P., Kiberd, B., Jardine, A., Levitt, N., Marchetti, P., Markell, M., Naicker, S., O'Connell, P., Schnitzler, M., Standl, E., Torregosa, J. V., Uchida, K., Valantine, H., Villamil, F., Vincenti, F., & Wissing, M. (2005). Guidelines for the treatment and management of new-onset diabetes after transplantation. *Clinical Transplantation*, *19*(3), 291–298. https://doi.org/10.1111/j.1399-0012.2005.00359.x
704. Williams, A., Crawford, K., Manias, E., Ellis, C., Mullins, K., Howe, K., Kennedy, E., Maney, O., Mark, T., Gregory, D., Van Hardeveld, E., Yip, D., & Low, J. K. (2015). Examining the preparation and ongoing support of adults to take their medications as prescribed in kidney transplantation. *Journal of Evaluation in Clinical Practice*, *21*(2), 180–186. https://doi.org/10.1111/jep.12270
705. Williams, A. F., Manias, E., Gaskin, C. J., & Crawford, K. (2014). Medicine non-adherence in kidney transplantation. *Journal of Renal Care*, *40*(2), 107–116. https://doi.org/10.1111/jorc.12063
706. Williams, A., Low, J. K., Manias, E., & Crawford, K. (2016). The transplant team's support of kidney transplant recipients to take their prescribed medications: A collective responsibility. *Journal of Clinical Nursing*, *25*(15–16), 2251–2261. https://doi.org/10.1111/jocn.13267
707. Williams, N. C., Tong, A., Howard, K., Chapman, J. R., Craig, J. C., & Wong, G. (2012). Knowledge, beliefs and attitudes of kidney transplant recipients regarding their risk of cancer. *Nephrology*, *17*(3), 300–306. https://doi.org/10.1111/j.1440-1797.2011.01549.x
708. Wiltshire, G., Clarke, N. J., Phoenix, C., & Bescoby, C. (2021). Organ Transplant Recipients' Experiences of Physical Activity: Health, Self-Care, and Transliminality. *Qualitative Health Research*, *31*(2), 385–398. https://doi.org/10.1177/1049732320967915
709. Wright, L. (2000). Mentorship programs for transplant patients. *Progress in Transplantation*, *10*(4), 267–272. https://doi.org/10.7182/prtr.10.4.6w26x244566347pv

710. Wu, S. Z., Jiang, P., DeCaro, J. E., & Bordeaux, J. S. (2016). A qualitative systematic review of the efficacy of sun protection education in organ transplant recipients. *Journal of the American Academy of Dermatology*, *75*(6), 1238-1244.e5. https://doi.org/10.1016/j.jaad.2016.06.031
711. Wu, Y. C., Tung, H. H., Lai, F. C., Wei, J., Liang, S. Y., & Wu, S. F. (2017). Lifestyle changes in heart transplant recipients. *International Journal of Nursing Practice*, *23*(1). https://doi.org/10.1111/ijn.12504
712. Xia, M., Yan, J., Liu, S., & Liu, J. (2019). Beliefs of Immunosuppressive Medication Among Chinese Renal Transplant Recipients, as Assessed in a Cross-Sectional Study With the Basel Assessment of Adherence to Immunosuppressive Medications Scale. *Transplantation Proceedings*, *51*(3), 742–748. https://doi.org/10.1016/j.transproceed.2018.10.029
713. Xie, J., Liu, J., Liu, M., Yan, J., Ding, S., & Ma, K. (2019). Self-management and Related Psychosocial Variables Among Renal Transplant Patients. *Transplantation Proceedings*, *51*(3), 734–741. https://doi.org/10.1016/j.transproceed.2019.01.033
714. Xing, L., Chen, Q.-Y., Li, J.-N., Hu, Z.-Q., Zhang, Y., & Tao, R. (2015). Self-management and self-efficacy status in liver recipients. *Hepatobiliary & Pancreatic Diseases International*, *14*(3), 253–262.
715. Xu, X. F., Feng, Y. T., Tian, Y. F., & Wang, H. Y. (2018). Pharmaceutical Care in Kidney Transplant Recipients: Behavioral and Physiologic Outcomes at 12 Months. *Transplantation Proceedings*, *50*(8), 2451–2456. https://doi.org/10.1016/j.transproceed.2018.04.049
716. Yan, L., Lin, J., Zhao, C., & Xiong, C. J. (2021). The effects of collaborative care model (CCM) guided nursing practice on the self-management and self-efficacy of kidney transplant recipients. *International Journal of Clinical and Experimental Medicine*, *14*(1), 356-364.
717. Yang, F. C., Wang, S. S., Chen, H. M., Chen, C. H., Pang, S. C., & Chen, C. M. (2020). Adaptation Process of Male Kidney Transplant Recipients During the Difficult Postoperative Recovery Stage at Home. *Transplantation Proceedings*, *52*(10), 3221–3225. https://doi.org/10.1016/j.transproceed.2020.06.020
718. Yang, F.-C., Chen, H.-M., Huang, C.-M., Hsieh, P.-L., Wang, S.-S., & Chen, C.-M. (2020). The Difficulties and Needs of Organ Transplant Recipients during Postoperative Care at Home: A Systematic Review. *International Journal of Environmental Research and Public Health*, *17*(16), 5798. https://doi.org/10.3390/ijerph17165798
719. Yang, F.-C., Chen, H.-M., Pong, S.-C., Chen, C.-H., Wang, S.-S., & Chen, C.-M. (2020). Difficulties and Coping Strategies of Kidney-transplant Recipients During Their Dark Postoperative Recovery Stage After Returning Home. *Transplantation Proceedings*, *52*(10), 3226–3230. https://doi.org/10.1016/j.transproceed.2020.05.011
720. Yektatalab, S., Beyrami, L., & Momennasab, M. (2019). The Effect of Coping Strategies Training on Coping and Health-Related Quality of Life in Patients after Liver Transplantation. *Focus on Medical Sciences Journal*, *5*(3).
721. Yoo, H. J., & Suh, E. E. (2021a). Effects of a smartphone-based self-care health diary for heart transplant recipients: A mixed methods study. *Applied Nursing Research*, *58*, 151408. https://doi.org/10.1016/j.apnr.2021.151408
722. Yoo, H. J., & Suh, E. E. (2021b). Lived Experiences of Korean Young Adults After Heart Transplantation: A Phenomenological Approach. *Asian Nursing Research*, *15*(2), 89–95. https://doi.org/10.1016/j.anr.2020.10.001
723. Yoon, H. J., Guo, H., Hertz, M., & Finkelstein, S. (2008). Adherence to home-monitoring and its impact on survival in post-lung transplantation patients. *AMIA Annual Symposium Proceedings*, 835–838.
724. Yoshikawa, Y., Uchida, J., Akazawa, C., & Suganuma, N. (2018). Associations between physical and psychosocial factors and health-related quality of life in women who gave birth after a kidney transplant. *International Journal of Women's Health*, *10*, 299–307. https://doi.org/10.2147/IJWH.S152750

725. Yoshikawa, Y., Uchida, J., Kosoku, A., Akazawa, C., & Suganuma, N. (2019). Childbirth and Care Difficulties of Female Kidney Transplantation Recipients. *Transplantation Proceedings*, *51*(5), 1415–1419. https://doi.org/10.1016/j.transproceed.2019.03.013
726. Yoshimura, Y., Umeshita, K., Kubo, S., & Yoshikawa, Y. (2016). Anxieties and coping methods of liver transplant recipients regarding pregnancy and delivery. *Journal of Advanced Nursing*, *72*(8), 1875–1885. https://doi.org/10.1111/jan.12957
727. Yurttas, A., & Nar, N. (2018). The feelings and concerns of patients with kidney transplantation in turkey: A qualitative study. *International Journal of Caring Sciences*, *11*(3), 1467–1474.
728. Zaldonis, J., Alrawashdeh, M., Atman, K. S., Fatigati, A., Dabbs, A. D., & Bermudez, C. A. (2015). Predictors and influence of goal orientation on self-management and health-related quality of life after lung transplant. *Progress in Transplantation*, *25*(3), 230–242. https://doi.org/10.7182/pit2015189
729. Zanetti-Yabur, A., Rizzo, A., Hayde, N., Watkins, A. C., Rocca, J. P., & Graham, J. A. (2017). Exploring the usage of a mobile phone application in transplanted patients to encourage medication compliance and education. *American Journal of Surgery*, *214*(4), 743–747. https://doi.org/10.1016/j.amjsurg.2017.01.026
730. Zare Moayedi, M., Aslani, A., Fakhrahmad, M., & Ezzatzadegan J, S. (2018). Developing an Android-Based Patient Decision Aid Based on Ottawa Standards for Patients After Kidney Transplant and Its Usability Evaluation. *Studies in Health Technology & Informatics*, *249*, 61–68. https://doi.org/10.3233/978-1-61499-868-6-61
731. Zare, N. V., Mohammadi, E., Zare, K., & Ehahi, N. (2017). Design and psychometric evaluation of coping scale in recipients of kidney transplant. *Evidence Based Care Journal*, *7*(3), 45–53. https://doi.org/10.22038/ebcj.2017.14130.1577
732. Zarifian, A. (2006). Symptom occurrence, symptom distress, and quality of life in renal transplant recipients. *Nephrology Nursing Journal*, *33*(6), 609–618.
733. Zawadzka, B., Zawadzka, S., Bedkowska-Prokop, A., Ignacak, E., & Sulowicz, W. (2016). Psychological Predictors of Cooperation in the Chronic Treatment of Kidney Transplantation Patients. *Transplantation Proceedings*, *48*(5), 1644–1649. https://doi.org/10.1016/j.transproceed.2016.03.001
734. Zehrer, C. L. (1994). Patient perceptions of benefits and concerns following pancreas transplantation. *Diabetes Educator*, *20*(3), 216–220. https://doi.org/10.1177/014572179402000307
735. Zelle, D. M., Corpeleijn, E., Klaassen, G., Schutte, E., Navis, G., & Bakker, S. J. (2016). Fear of Movement and Low Self-Efficacy Are Important Barriers in Physical Activity after Renal Transplantation. *PLoS One*, *11*(2), e0147609-undefined. https://doi.org/10.1371/journal.pone.0147609
736. Zelle, D. M., Kok, T., Dontje, M. L., Danchell, E. I., Navis, G., van Son, W. J., Bakker, S. J., & Corpeleijn, E. (2013). The role of diet and physical activity in post-transplant weight gain after renal transplantation. *Clinical Transplantation*, *27*(4), E484-90. https://doi.org/10.1111/ctr.12149
737. Zhang, M., Zhou, H., Nelson, R. S., Han, Y., Wang, Y., Xiang, H., Cai, J., Zhang, J., & Yuan, Y. (2019). Prevalence and risk factors of immunosuppressant nonadherence in heart transplant recipients: A single-center cross-sectional study. *Patient preference and adherence*, *13*, 2185–2193. https://doi.org/10.2147/PPA.S223837
738. Zhang, W., Zeng, L., Li, J., Deng, X., Jin, M., Chen, Z., Fang, J., & Yu, H. (2020). Construction and reliability and validity tests of the dietary self-management ability scale for kidney transplant recipients. *Annals of Palliative Medicine*, *9*(2), 352–358. https://doi.org/10.21037/apm.2020.03.07
739. Zhao, L., Yan, J., Yang, G. L., & Liu, Y. (2017). A Study on Adherence to Follow-up, Quality of Life, and Associated Factors Among Renal Transplant Recipients in China. *Transplantation Proceedings*, *49*(6), 1285–1290. https://doi.org/10.1016/j.transproceed.2017.03.086

740. Ziegelmann, J. P., Griva, K., Hankins, M., Harrison, M., Davenport, A., Thompson, D., & Newman, S. P. (2002). The Transplant Effects Questionnaire (TxEQ): The development of a questionnaire for assessing the multidimensional outcome of organ transplantation—Example of end stage renal disease (ESRD). *British Journal of Health Psychology*, *7*(Part 4), 393–408. https://doi.org/10.1348/135910702320645381
741. Zimmermann, T., Pabst, S., Bertram, A., Schiffer, M., & de Zwaan, M. (2016). Differences in emotional responses in living and deceased donor kidney transplant patients. *Clinical Kidney Journal*, *9*(3), 503–509. https://doi.org/10.1093/ckj/sfw012
742. Zumbrunnen, R. (1989). Coping with heart transplantation: A challenge for liaison psychiatry. *Psychotherapy & Psychosomatics*, *52*(1–3), 66–73. https://doi.org/10.1159/000288301
