# Supplementary material for: Aspects of Self-Management After Solid Organ Transplantation—A Scoping Review
Source: Nurs Rep. 2025 Aug 19;15(8):304. doi: 10.3390/nursrep15080304 (PMC12389569; doi:10.3390/nursrep15080304)
Supplement: Supplementary file 1 [file nursrep-15-00304-s001.zip › Supplementary File S4.pdf]

Supplementary File S4: Coding frame detailing all category levels

Titles (Main category; category; sub-category)	Number of documents with codes for corresponding categories
1. Managing the medical-therapeutic regimen	n=685
1.1. Managing key elements of the therapy	n=575
1.1.1. Managing medication	n=445
1.1.2. Self-monitoring indicators of health***	n=225
1.1.3. Managing symptoms and side effects	n=190
1.1.4. Keeping regular appointments	n=184
1.1.5. Understanding transplantation and its therapy***	n=57
1.1.6. Building a partnership with healthcare providers	n=67
1.1.7. Caring for surgical wound/drains*	n=10
1.2. Managing one's health related lifestyle	n=448
1.2.1. Eating healthily and appropriately	n=251
1.2.2. Exercising/Physical activity	n=250
1.2.3. Preventing and controlling infections	n=121
1.2.4. Refraining from harmful use of substances	n=96
1.2.5. Preventing skin cancer ***	n=93
1.2.6. Managing body weight	n=78
2. Managing biographical work*	n=201
2.1. Experiencing and managing emotions***	n=162
2.2. Managing the self-concept***	n=96
2.2.1. Integrating the new organ**/***	n=41
2.2.2. Managing reduced energy and physical capacity**	n=33
2.2.3. Dealing with changes in appearance**	n=22
3. Managing (new) life roles	n=226
3.1. Managing changes in social fabric***	n=168
3.1.1. Seeking support from others*	n=68
3.1.2. Maintaining and adapting relationships and social roles	n=56
3.1.3. Managing intimate relationships	n=42
3.2. Adjusting to (a new) normality or reality	n=82
3.3. Performing everyday activities§	n=81
4. Generic self-management skills, strategies and processes*	n=240
4.1. Self-management skills	n=133
4.1.1. Taking action§	n=80
4.1.2. Health literacy*	n=77
4.2. Strategies and processes in the context of self-management*	n=159
4.2.1. Acceptance*	n=62
4.2.2. Self-efficacy and resilience*	n=42
4.2.3. Hope and optimism*	n=31
4.2.4. Setting goals and priorities and develop plans*	n=27
4.2.5. Spirituality *	n=19
4.2.6. Adopting a new mindset *	n=19

Legend: * created inductively; ** moved to another main category/category in alignment with the initial category system; *** renamed in alignment with the initial category system; § subsumed categories of the initial category system
